# The complexity of examining laboratory-based biological markers associated with mortality in hospitalized patients during early phase of the COVID-19 pandemic: A systematic review and evidence map

**DOI:** 10.1371/journal.pone.0273578

**Published:** 2022-09-09

**Authors:** Lauren E. Griffith, Muhammad Usman Ali, Alessandra Andreacchi, Mark Loeb, Meghan Kenny, Divya Joshi, Vishal Mokashi, Ahmed Irshad, Angela K. Ulrich, Nicole E. Basta, Parminder Raina, Laura Anderson, Cynthia Balion

**Affiliations:** 1 Department of Health Research Methods, Evidence and Impact, McMaster University, Hamilton, Ontario, Canada; 2 Labarge Centre for Mobility in Aging, McMaster University, Hamilton, Ontario, Canada; 3 McMaster Institute for Research on Aging, McMaster University, Hamilton, Ontario, Canada; 4 Department of Pathology and Molecular Medicine, McMaster University, Hamilton, Ontario, Canada; 5 School of Life Sciences, McMaster University, Hamilton, Ontario, Canada; 6 Faculty of Health Sciences, McMaster University, Hamilton, Ontario, Canada; 7 Center for Infectious Disease Research and Policy, University of Minnesota, Minneapolis, MN, United States of America; 8 Division of Environmental Health Sciences, School of Public Health, University of Minnesota, Minneapolis, MN, United States of America; 9 Department of Epidemiology, Biostatistics, and Occupational Health, McGill University, Montreal, Quebec, Canada; University of Maryland School of Medicine, UNITED STATES

## Abstract

**Importance:**

The measurement of laboratory biomarkers plays a critical role in managing patients with COVID-19. However, to date most systematic reviews examining the association between laboratory biomarkers and mortality in hospitalized patients early in the pandemic focused on small sets of biomarkers, did not account for multiple studies including patients within the same institutions during overlapping timeframes, and did not include a significant number of studies conducted in countries other than China.

**Objective:**

To provide a comprehensive summary and an evidence map examining the relationship between a wide range of laboratory biomarkers and mortality among patients hospitalized with COVID-19 during the early phase of the pandemic in multiple countries.

**Evidence review:**

MEDLINE, EMBASE, and Web of Science were searched from Dec 2019 to March 9, 2021. A total of 14,049 studies were identified and screened independently by two raters; data was extracted by a single rater and verified by a second. Quality was assessed using the Joanna Briggs Institute (JBI) Case Series Critical Appraisal tool. To allow comparison across biomarkers, standardized mean differences (SMD) were used to quantify the relationship between laboratory biomarkers and hospital mortality. Meta-regression was conducted to account for clustering within institutions and countries.

**Results:**

Our systematic review included 94 case-series studies from 30 countries. Across all biomarkers, the largest and most precise SMDs were observed for *cardiac* (troponin (1.03 (95% CI 0.86 to 1.21)), and BNP/NT-proBNP (0.93 (0.52 to 1.34)), *inflammatory* (IL-6 (0.97 (0.67 to 1.28) and Neutrophil-to-lymphocyte ratio (0.94 (0.59 to 1.29)), and *renal* biomarkers (blood urea nitrogen (1.01 (0.79 to 1.23)) and estimated glomerular filtration rate (-0.96 (-1.42 to -0.50)). There was heterogeneity for most biomarkers across countries with studies conducted in China generally having larger effect sizes.

**Conclusions and relevance:**

The results of this study provide an early pandemic summary of the relationship between biomarkers and mortality in hospitalized patients. We found our estimated ESs were generally attenuated compared to previous systematic reviews which predominantly included studies conducted in China. Despite using sophisticated methodology to examine studies across countries, heterogeneity in reporting of case-series studies early in the pandemic limits clinical interpretability.

## Introduction

The World Health Organization characterized Coronavirus disease 2019 (COVID-19) as a pandemic in March 2020 [1]. The pandemic has been devastating and despite advances in prevention and treatment, mortality rates remain high. People with COVID-19 require hospitalization and intensive care admission which has negatively impacted health-care resource availability [2]. In fact, it is estimated that 349 million people (4% of the global population) are at high risk of severe COVID-19 that would require hospitalization if infected [3]. As such, there was an influx of case-report studies early in the pandemic examining prognostic factors, such as laboratory biomarkers, that could guide the stratification of COVID-19 patients based on their risk of severe disease or death. Laboratory testing and the measurement of appropriate biomarkers play a critical role in managing patients with COVID-19, allowing for disease diagnosis, monitoring progression, prognostication, and prediction of treatment response [4].

Summarizing studies in the early pandemic provides critical baseline information for comparison over the course of the pandemic with changes in vaccination rates, treatments, and the virus itself. Many early systematic reviews of laboratory biomarkers and COVID-19 outcomes focused on disease severity which is challenging to interpret as definitions of “severe” and “non-severe” disease differ extensively across studies and have changed over time. Fewer systematic reviews examined objective outcomes such as mortality. Of these reviews, most included only single biomarkers (e.g., D-Dimer) or specific classes of biomarker (e.g., cardiovascular biomarkers). These reviews provided a limited view of the relationship between biomarkers and mortality. Of the few reviews that included meta-analyses of several biomarkers [5–10], most predominantly included studies conducted in China [5–9] and some had methodological concerns because they failed to account for multiple studies including patients from the same institutions during overlapping timeframes [6, 10] which is a potential source of bias when summarizing this evidence [11]. The predominance of studies conducted in China did not allowed researchers to assess the heterogeneity of results across countries and raised the question as to whether or not these findings were broadly generalizable. The objectives of our systematic review were: 1) to provide a comprehensive summary and an evidence map examining the association between a wide range of laboratory biomarkers and mortality among patients hospitalized with COVID-19 early in the pandemic in multiple countries and continents, and 2) to evaluate if the magnitude of effect observed in the relationship between individual biomarkers and mortality differed by country.

## Methods

The systematic review protocol was registered on PROSPERO (CRD42020200682). Given the unexpectedly large number of studies that emerged on this topic after completion of the study protocol, we limited this review to mortality outcomes only even though our initial registration included additional outcomes. The present study is reported following the Preferred Reporting Items for Systematic Reviews (PRISMA; S1 Appendix) and Meta-analyses of Observational Studies (MOOSE; S2 Appendix) guidelines [12].

### Eligibility criteria

We included all published studies and did not restrict on study designs as long as there was a control group (i.e., comparing non-survivors to survivor controls). The target population was adult patients admitted to the hospital for COVID-19. Studies were included if they reported numerical hospital-based laboratory biomarker values and an estimate of precision at hospital admission (or earliest thereafter) for patients who survived and those who died in hospital. We excluded studies that presented data only on specific subgroups of patients (e.g., cancer patients, pregnant women). Studies were excluded if they did not provide continuous biomarker values (i.e., categorical summary of biomarkers using cut points were excluded) or were published in a language other than English.

### Search strategy

The original search for COVID-19 literature was conducted from December 2019-March 2020 in OVID MEDLINE In-Process, EMBASE, and Web of Science and an updated search extended the period to March 9, 2021 to maximize the inclusion of studies published during the early phase of the pandemic (late 2019 to early 2021) [13]. Additional studies were identified by checking abstracts and reference lists of included articles and systematic reviews; we did not contact study authors. The search strategy is detailed in Table S1 in S3 Appendix.

The search results were checked for duplicates and then screened for relevance using DistillerSR software [14] in two stages: 1) title and abstract and 2) full-text. The screening forms for both stages were pre-piloted and included criteria pertaining to the target population, study design, biomarkers, and health outcomes. Screening was done in duplicate by two independent raters with training in epidemiology and systematic review methodology. Discrepancies were resolved by discussion or a third reviewer if consensus was not possible.

The original search predominantly identified studies conducted in China. As well, we found multiple studies that likely included patients from the same institutions based on overlapping timeframes. The purpose of the updated search was to better reflect the possible differential effect on outcome based on diversity in the population (i.e. patient populations outside of China) and further enhance the generalizability of results. To make the task more feasible, we retained the inclusion and exclusion criteria from the original review but included additional criteria to select studies with the most precise estimates for each country. The additional criteria considered were: sample size, date of enrollment, site, population, and number of biomarkers reported. For each country a maximum of 4 studies that had the largest sample sizes (with non-overlapping patient populations) and the most biomarkers were included. Table S2 in S3 Appendix includes all eligible studies and those that were chosen for inclusion.

### Quality assessments and data extraction

Quality assessment was conducted using the Joanna Briggs Institute (JBI) Case Series Critical Appraisal tool [15]. Quality assessment and data extraction was done by one rater and verified by a second rater. The extracted information included publication details, study characteristics, population characteristics, hospital admission start/end date, method of SARS CoV-2 diagnosis, biomarkers measured and their baseline level in patients who survived and patients who died.

### Data presentation and analysis

Descriptive statistics were used to summarize studies relating hospital-based laboratory biomarkers to mortality in patients with COVID-19 including study characteristics and the number and type of biomarkers included. Table S3 in S3 Appendix describes how biomarkers were organized by clinical category (e.g., anemia, cardiac, coagulation, hepatic, inflammation, metabolic, pulmonary, renal).

For quantitative synthesis, we used baseline biomarker data (means, standard deviations) stratified by mortality outcome (died vs. survived). In situations where median and interquartile range were reported, we transformed to mean and standard deviation using optimal methods and converted estimates were checked for possible skewness away from normality [16–18]. We used a random effects multi-level meta-regression approach to account for dependency among effect sizes (ES) (i.e., the correlation among effect estimates due to overlap of patient population across studies conducted within same country, site (i.e., hospital), and time period of recruitment). In such cases, we nested the correlated ESs by introducing a random effect to our grouping variables (country and site within country) and the variance in observed ESs was decomposed into sampling variance, within country/site variance and between country/site variance to account for intra-cluster (or intraclass) correlation in the true effect [19, 20]. We assumed that these grouping variables are nested, in the sense that several ESs on level 2 (represented by each study) together make up a larger cluster on level 3 (represented by each country). For each biomarker we estimated an overall effect across all studies and a country-specific effect. The statistical heterogeneity I^2^ statistic was also estimated in the context of multi-level meta-analytical approach. I^2^_level 2_ was estimated to account for the amount of within-cluster heterogeneity, I^2^_level 3_ was estimated to account for between-cluster heterogeneity and I^2^_total_ was estimated to represent the heterogeneity not attributable to sample error and is the sum of values at level 2 and 3. I^2^_total_ was reported for the overall pooled effect estimates [19, 20].

Standardized mean differences (SMDs) were used as summary estimates so ESs could be compared across biomarkers based on magnitude. SMDs were calculated using the inverse variance method in a random effects model using bias corrected Hedges adjusted g formula [21]. SMDs (Hedges g) were interpreted based on magnitude according to Cohen’s *d* recommended thresholds (0–0.19 trivial effect, 0.2–0.49 = small effect, 0.5–0.79 = medium effect, 0.8–1.59 = large effect, and >1.6 = very large effect) [22].

Meta-analyzed SMDs for laboratory biomarkers were grouped by clinical category and by country in which the study was undertaken. To aid interpretation, an evidence map was constructed to reflect the most robust and generalizable findings. This figure included only statistically significant SMDs that represented data from at least 4 countries. Biomarkers were sorted by summary SMD magnitude. Country-specific SMDs were also calculated for each biomarker and compared qualitatively using a heat map in which the darkness of the cell reflects the magnitude of the SMD. All analyses were performed using R software (metafor and dmetar packages) [23, 24]. Evidence maps are presented utilizing methods described by Katz [25] and Miake-Lye [26].

## Results

**Fig 1:**
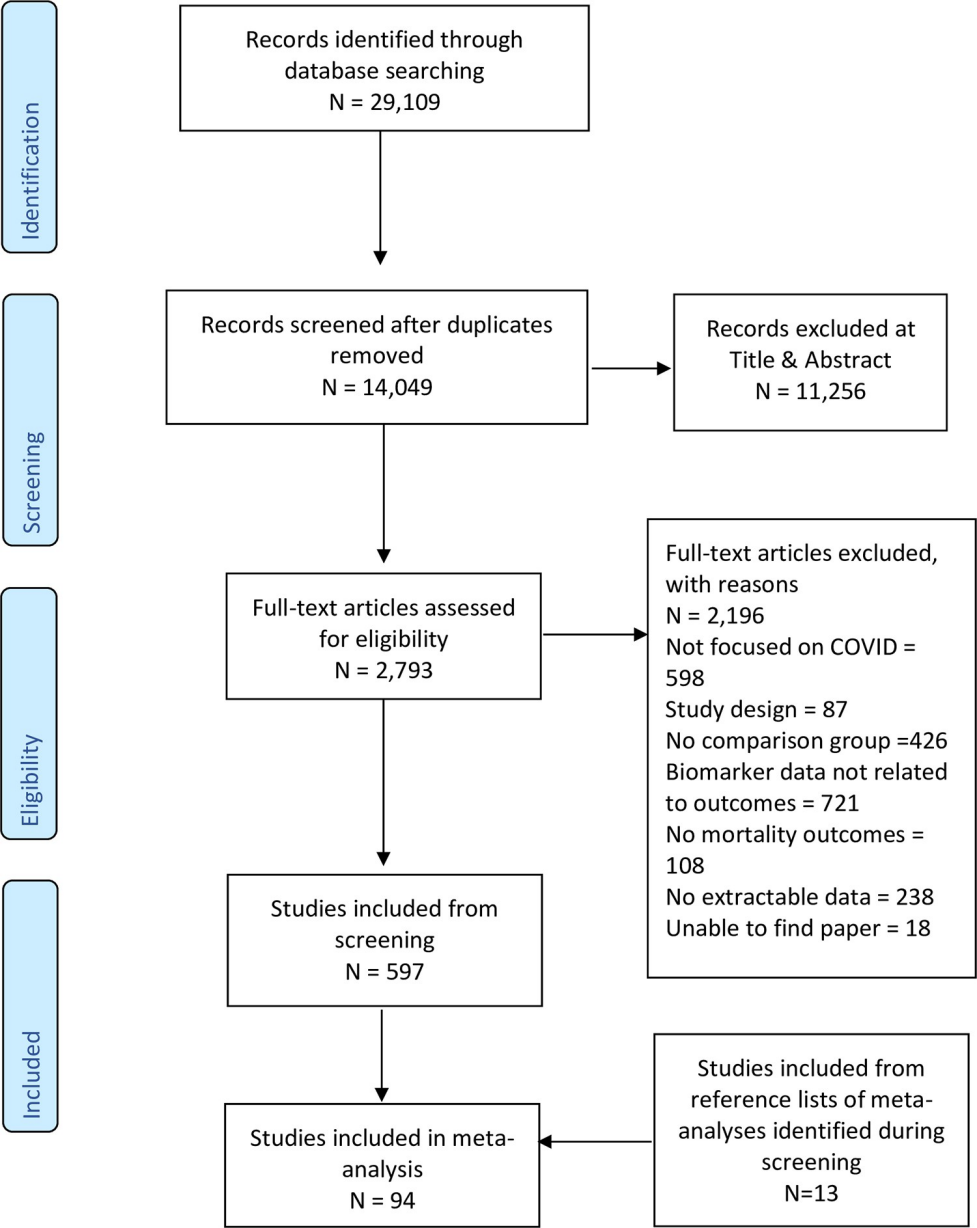
PRISMA flow diagram for studies evaluating laboratory-based biomarkers in relation to mortality.

Two reviewers independently screened a total of 14,049 records after duplicate removal, identifying 2,793 publications for full-text screening. In total, 94 publications were included in this review as described in the PRISMA Flow Diagram (Fig 1).

The included studies measured biomarkers in hospitalized patients with a laboratory-confirmed diagnosis of COVID-19 with admission dates starting between Dec 24, 2019 and June 1, 2020 (Table 1). Of the 94 included studies, 50 (67.6%) were conducted in Asia, 27 (36.5%) in Europe, 7 (9.5%) in North America, 4 (4.4%) in Africa, 3 (3.2%) in South America, and 3 (2.1%) included patients from multiple countries. Most of the studies conducted in China began in the early months of the pandemic (January and February, 2020) and studies in other countries predominantly starting in mid-February and March, 2020 (Fig S52 in S3 Appendix). The majority included patients from a single hospital 73 (77.6%) and of these 10 studies specifically looked at patients from the ICU. The sample size ranged from 19 to 4,244 patients with a median of 197 patients.

**Table 1:** Descriptive characteristics of included studies comparing hospitalized SARS CoV-2 survivors and non-survivors (n = 94).

Characteristics of Included Studies	*n* (%)
**Continent / Country of Participants**	
Africa	
Algeria	1 (1.1)
Morocco	1 (1.1)
South Africa	1 (1.1)
Sudan	1 (1.1)
Asia	
China	21 (22.3)
India	4 (4.3)
Indonesia	2 (2.1)
Iran	4 (4.3)
Israel	2 (2.1)
Korea/South Korea	4 (4.3)
Kuwait	2 (2.1)
Pakistan	4 (4.3)
Saudi Arabia	3 (3.2)
Turkey	4 (4.3)
Europe	
Belgium	2 (2.1)
France	3 (3.2)
Germany	2 (2.1)
Greece	2 (2.1)
Italy	4 (4.3)
Norway	1 (1.1)
Poland	2 (2.1)
Spain	4 (4.3)
Sweden	1 (1.1)
Switzerland	1 (1.1)
Ukraine	1 (1.1)
United Kingdom	4 (4.3)
North America	
Mexico	3 (3.2)
United States	4 (4.3)
South America	
Brazil	3 (3.2)
More than 1 country	3 (2.1)
**Study Setting**	
Hospital	63 (67.0)
>1 Hospital	18 (19.1)
ICU	10 (10.6)
ICUs from >1 Hospital	3 (3.2)
**Range of Publication Dates** (*n* = 93)	2020-02-19 to 2021-02-25
**Range of Admission Start Dates** (*n* = 83)	2019-12-24 to 2020-06-01
**Range of Admission End Dates** (*n* = 86)	2020-01-26 to 2020-10-25
**Method of SARS CoV-2 Diagnosis**	
PCR	72 (76.6)
PCR AND/OR clinical diagnosis (e.g. symptoms/chest CT/chest x-ray/exposure to a positive case)	12 (12.8)
PCR AND/OR anti-SARS-CoV-2 IgM and/or IgG antibody assay	1 (1.1)
PCR AND clinical diagnosis (e.g. symptoms/chest CT/chest x-ray/exposure to a positive case) AND anti-SARS-CoV-2 IgM and/or IgG antibody assay for RT-PCR-negative patients	2 (2.1)
PCR OR anti-SARS-CoV-2 IgM and/or IgG antibody assay	1 (1.1)
Unclear	4 (4.3)
Not Reported	2 (2.1)
**Sample size** (*n* = 94)	
Mean (SD)	577.8 (875.5)
Median (IQR) [range]	196.5 (103.8–467.3) [19–4244]
**Average Age of Participants** (*n* = 92)	
Mean (SD)	60.9 (9.4)
Median (IQR) [Range]	61.4 (54.5–66.2) [40.0–89.3]
**Proportion of Male Participants** (*n* = 94)	
Mean (SD)	60.1 (11.5)
Median (IQR) [Range]	59.1 (52.6–69.0) [32.8–87.2]

All studies were case series regardless of how they were defined by the authors, as they enrolled and followed hospitalized COVID-19 patients comparing those who survived versus those who died. Therefore, we appraised all studies using JBI for case series (Table S14 in S3 Appendix). Over 90% of studies provided clear criteria for inclusion, diagnosed COVID-19 in a standard and reliable way, clearly reported their results and demographic information for their sample, and used appropriate statistical analysis, 71.3% reported demographic information for the hospital site, and less than 50% included consecutive patients and/or all eligible patients over a specific time frame.

### Laboratory biomarkers (Tables S4-S13 in S3 Appendix)

The most commonly included biomarkers were lymphocytes, C-reactive protein (CRP), and creatinine which were included in 76.6%, 73.4% and 70.2% of the studies, respectively. Over half of the studies also include hemoglobin, platelets, D-dimer, alanine aminotransferase (ALT), lactate dehydrogenase (LDH), aspartate aminotransferase (AST), white blood cells, and neutrophils. Almost all studies included at least one inflammatory biomarker (91.5%); 84.0% included at least one coagulation biomarker, 78.7% included at least one hepatic biomarker and 76.6% included at least one renal biomarker.

### Overall association with mortality

**Fig 2:**
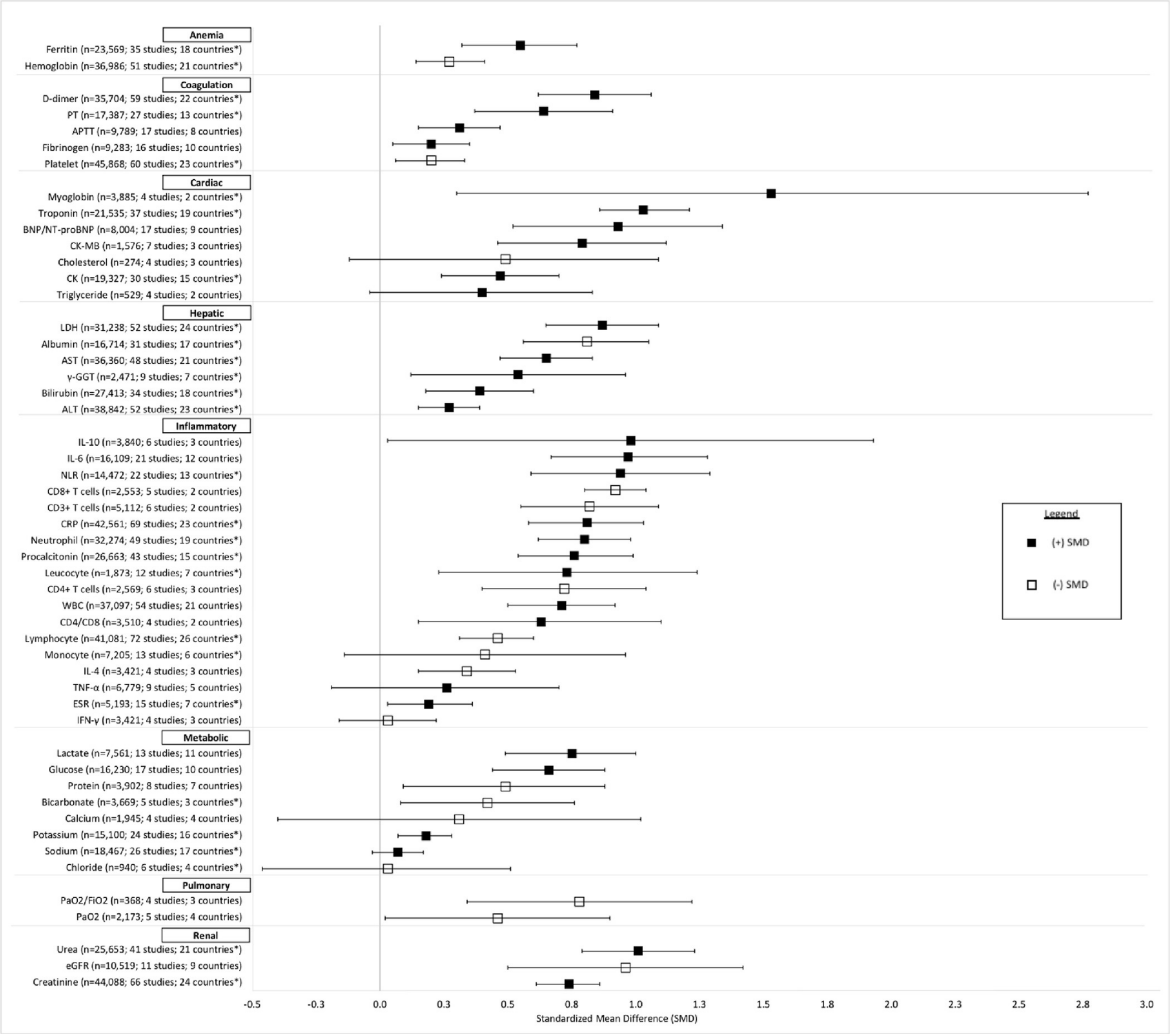
Forest plot of Standardized Mean Difference (SMD) for all laboratory biomarkers sorted by clinical category. The SMD reflects the difference in the average biomarker level in patients who died compared to those who survived in standard deviation units. SMDs represented by black squares are positive (the average biomarker level in those who died are higher than the level in those who survived) and SMDs represented by white squares are negative (the average biomarker level in those who died are lower than the level in those who survived).

**Fig 3:**
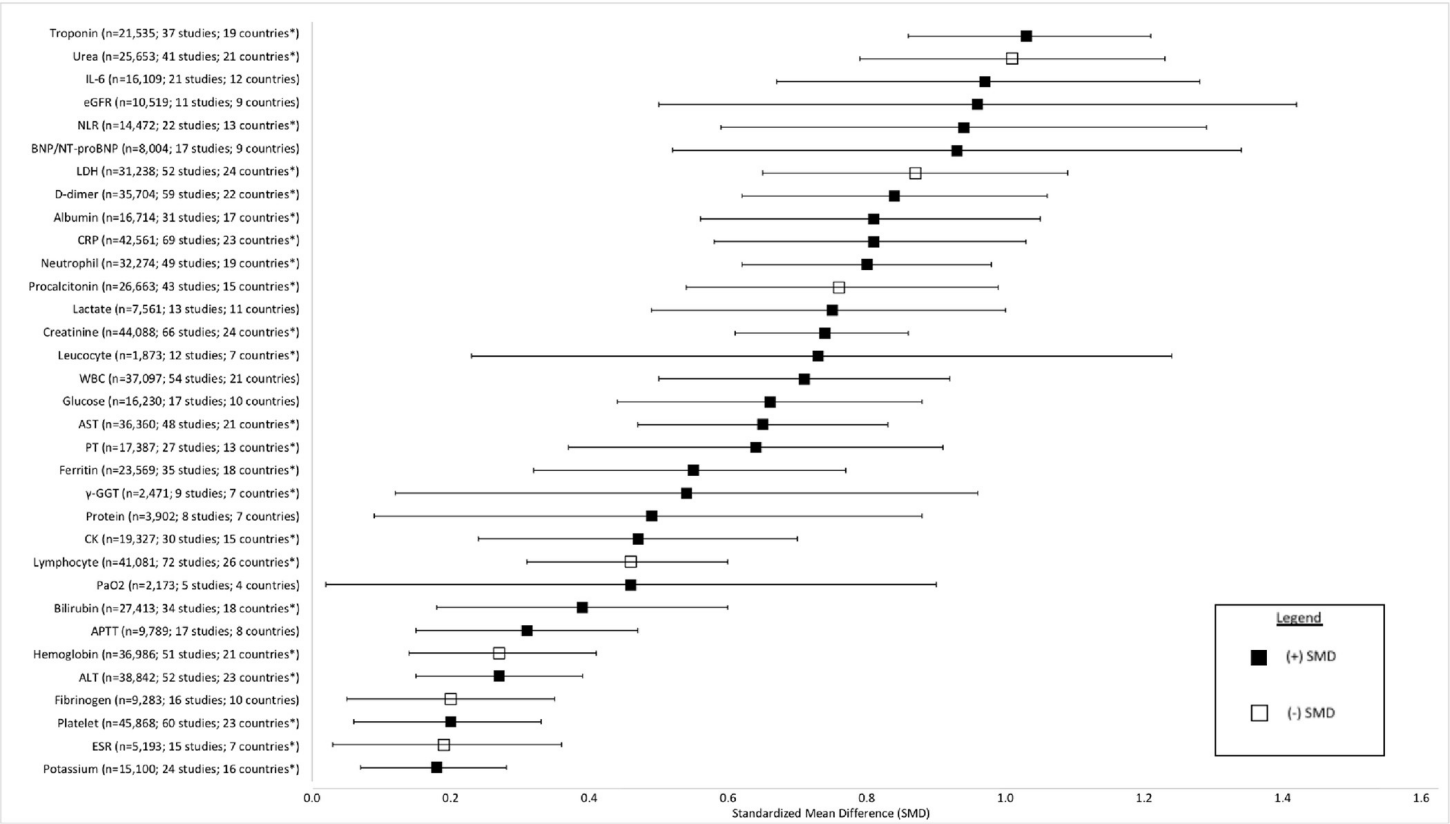
Summary of meta-analyzed standardized mean differences (SMD) for laboratory biomarkers. Biomarkers are sorted by SMD magnitude and included if they are statistically significant and represent data from at least 4 countries. The SMD reflects the difference in the average biomarker level in patients who died compared to those who survived in standard deviation units. SMDs represented by black squares are positive (the average biomarker level in those who died are higher than the level in those who survived) and SMDs represented by white squares are negative (the average biomarker level in those who died are lower than the level in those who survived).

Fig 2 summarized all biomarker SMDs organized by clinical category (Individual biomarker results are in Figs S1-S51 in S3 Appendix). Among cardiac biomarkers, the largest SMDs were observed for myoglobin, troponin and BNP/NT-proBNP with SMDs of 1.53 (95% CI 0.30 to 2.77), 1.03 (95% CI 0.86 to 1.21) and 0.93 (95% CI 0.52 to 1.34) respectively. Among inflammatory biomarkers, the largest SMDs were observed for interleukins (0.97 (95% CI 0.67 to 1.28) for IL-6 and 0.98 (95% CI 0.03 to 1.93) for IL-10 and Neutrophil-to-lymphocyte ratio (NLR) 0.94 (95% CI 0.59 to 1.29). Across renal biomarkers, the largest SMDs were for blood urea nitrogen and estimated glomerular filtration rate (eGFR) (1.01(95% CI 0.79 to 1.23)) and (-0.96 (95% CI -1.42 to -0.50) respectively. Lactate dehydrogenase (0.87 (95% CI 0.65 to 1.09)) and albumin (-0.81 (95% CI -1.06 to -0.57)) had the largest SMDs among hepatic biomarkers and D-dimer (0.84 (95% CI 0.62 to 1.06) for coagulation biomarkers. No other categories of biomarkers had large effect sizes (i.e., >0.80). Fig 3 summarizes the most robust evidence including biomarkers that were statistically significant and included studies from at least 4 countries. The largest effect sizes overall were associated with cardiac (Troponin and BNP/NT-proBNP), renal (Urea and eGFR), and inflammatory (IL-6 and NLR) biomarkers.

### Country-specific estimates

Fig 4A and 4B presents a heat map of country-specific biomarker SMDs in which each row represents a biomarker and each column represents a country. The color of the cell reflects the absolute value of the ES from light peach being trivial (ES<0.2) to dark orange being very large (ES≥1.6); statistical significance (p<0.05) is indicated by an asterisk in the center of the cell. There were some differences in the magnitude of SMDs among countries (see also Figs S1-S51 in S3 Appendix). For example, studies conducted in China tended to have higher SMDs whereas studies form the UK, US, and multinational studies tended to have lower ESs for the same biomarkers. All countries that evaluated troponin and BNP had significant SMD except for Pakistan.

**Fig 4:**
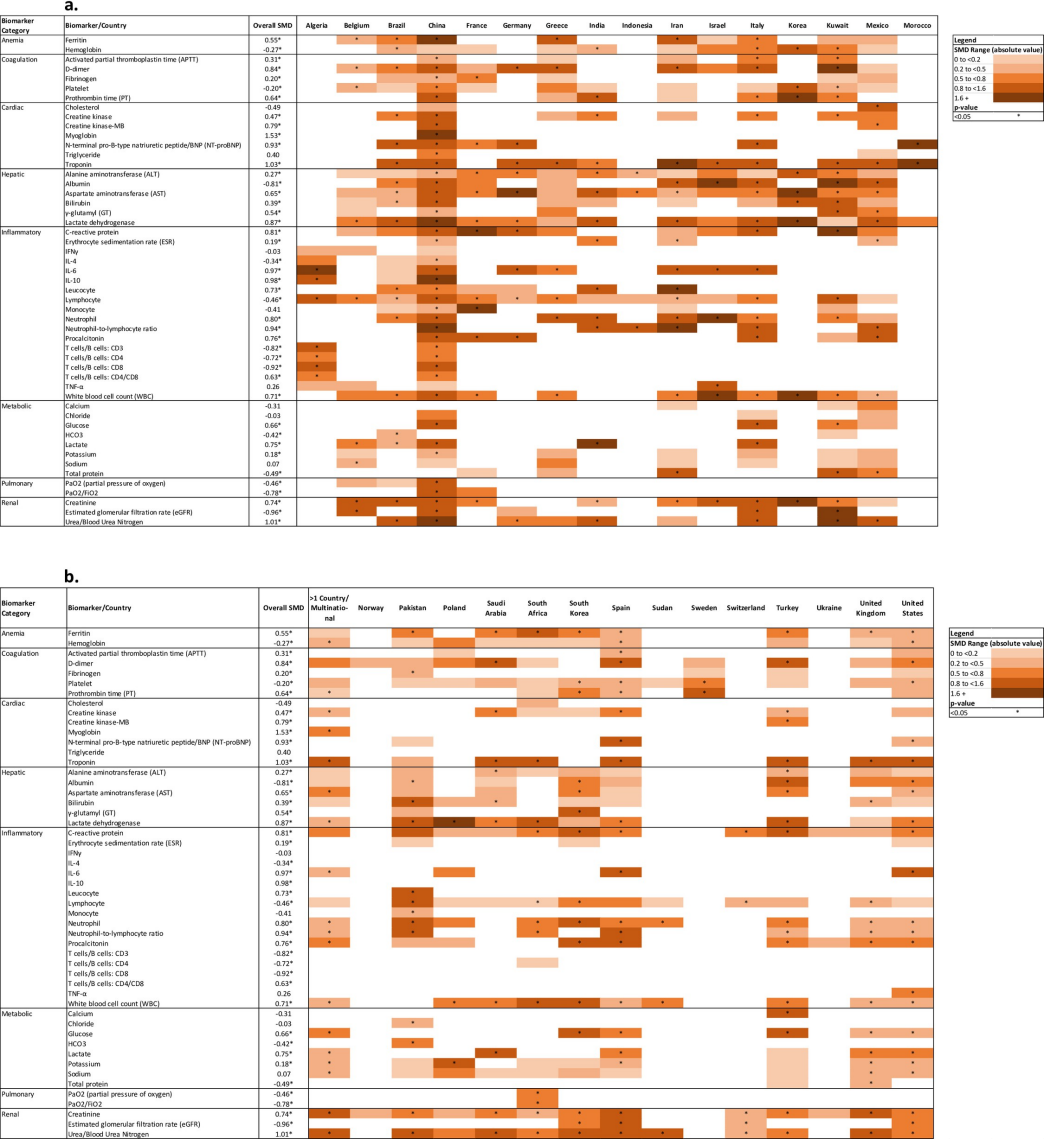
Heat map of alphabetically ordered country-specific standardized mean differences (SMD) by biomarker category. The SMD reflects the difference in the average biomarker level in patients who died compared to those who survived in standard deviation units. The darkness of the cell reflects the magnitude of the absolute value of the SMD with the lightest shade indicating a trivial SMD (0 to 0.19) and the darkest shade indicating a very large SMD (>1.5). Fig 4A includes results for Algeria-Morocco and Fig 4B includes results for muti-national studies-United States.

## Discussion

In this methodologically rigorous systematic review, we provide a comprehensive analysis of biomarker levels in hospitalized patients that died of COVID-19 compared to those who survived early in the pandemic. We found there was a number of cardiac, inflammatory, renal, hepatic, and coagulation biomarkers measured at or soon after hospital admission that were associated with mortality. As most of the early systematic reviews included studies predominantly from China, we purposely included studies from multiple countries and continents to increase the generalizability of the results and to examine how results may differ.

We found a wide range of biomarkers were reported across studies in relation to mortality and over 75% of studies included at least one inflammatory, coagulation, hepatic or renal biomarker. Over half of the inflammatory, cardiac, and hepatic biomarkers examined had a medium or large ES, with the largest ES associated with IL-10, IL-6, and NLR (inflammatory) and myoglobin, troponin and BNP/NT-proBNP (cardiac), and LDH (hepatic). Of the three renal biomarkers, urea and eGRF had large ESs; most of the ESs for the coagulation biomarkers were small with only D-dimer having a large ES. It is also noteworthy that we found heterogeneity associated with almost all meta-analyses and only three of the significant ESs had an I^2^<50%. However, these findings are not entirely unexpected as total I^2^ from multi-level meta-analysis is a sum of heterogeneity variances for both within country and across countries studies estimates as opposed to just across studies variance in a conventional 2-stage meta-analysis. These results reflect the difference in the magnitude of the ESs found among countries.

Compared to previous systematic reviews, we generally found similar results in terms of which biomarkers were associated with the largest ESs (Troponin, Urea, IL-6, eGFR, NLR, BNP/NT-proBNP, etc.), however in many cases our estimated ESs were attenuated compared to earlier systematic reviews [6–10]. For example, the ES Urea and IL-6 reported by Mesas et al. were 2.43, and 2.31, compared to our estimates of 1.01 and 0.97. This may be due to the preponderance of studies conducted in China in the earlier reviews. Although it was not uniformly the case (e.g., Troponin), our heat map suggests the magnitude of ESs for many biomarkers tended to be higher in studies conducted in China. It is not clear whether this could be related to the outcomes of COVID-19 being more severe early in the pandemic. It is also possible that the methodologic quality of the early-pandemic studies was lower than those conducted later. Compared to studies conducted in China, early pandemic studies conducted in other countries more often reported clear inclusion criteria (94.5% vs. 81.6%; P = 0.049) and clearly report demographics of the participants (98.6% vs. 85.7%; P = 0.01) (Table S15 in S3 Appendix).

Our systematic review is unique in that we included studies from 30 countries and used statistical methods to account for the bias that can be introduced when including non-independent studies. We calculated a summary SMD overall and by country to account for differences in condition severity and/or health care services quality/availability. We also focused on an outcome, mortality, that was measured consistently across studies compared to severity which was operationalized differently across studies and included a comprehensive list of laboratory based-biomarkers to depict a true landscape of hospital-based COVID-19 research as opposed to select few biomarkers or biomarker categories. Our review also has some weaknesses. For feasibility reasons we did not include all published studies in our search date range, but rather we chose studies in a way to maximize precision and regional diversity to enhance generalizability. While it is possible the larger studies were associated with lower quality, generally larger sample sizes increase the precision and robustness of results [27]. While we identified an immense amount of literature, including studies conducted in over 30 countries, we also restricted our search to studies published in English. Most importantly, there was variation in hospital admission and enrollment criteria across studies published early in the pandemic and we were not able to select or stratify on whether participants were treated with medication, supplemental oxygen, or mechanical ventilation, were admitted to a general hospital unit or designated COVID hospital, or the severity of their condition which could impact biomarker values. Studies were not always described sufficiently to know if all laboratory values were collected at admission; we selected the earliest or first laboratory value if more than one was given. Finally, we were unable to determine the time between symptom onset and measurement of laboratory values or the time between measurement of laboratory biomarkers and death or discharge.

This review provides a snapshot of the relationship between laboratory biomarkers and mortality during the early phase of the COVID-19 pandemic. We observed strong relationship between several biomarkers and mortality, however the effect sizes tended to be attenuated compared to earlier reviews. Further meta-regression analysis revealed larger effect sizes were generally observed for studies conducted in China. While we tried to overcome many deficiencies in previous reviews by utilizing a rigorous methodological approach to quantitative synthesis, the inconsistency and heterogeneity in reporting important clinical characteristics limits our ability to make clinical inferences with certainty along with direct clinical utility of our findings. Such important clinical characteristics include variation in hospital admission, enrollment criteria, medication, use of supplemental oxygen or mechanical ventilation, the severity of the disease at baseline, and the time between symptom onset and measurement of laboratory values. Although reporting guidelines for case-report studies exist [28], the importance of publishing evidence early in the pandemic may have led to reporting inconsistencies. As the pandemic continues, consistent reporting of key clinical characteristics as well as vaccination status, treatments, and COVID-19 variants will improve the clinical utility of systematic review findings.

## Supporting information

S1 Appendix PRISMA checklist. (PDF)

S2 Appendix MOOSE checklist. (PDF)

S3 Appendix (DOCX)
