# Supplementary material for: The complexity of examining laboratory-based biological markers associated with mortality in hospitalized patients during early phase of the COVID-19 pandemic: A systematic review and evidence map
Source: PLoS One. 2022 Sep 9;17(9):e0273578. doi: 10.1371/journal.pone.0273578 (PMC9462773; doi:10.1371/journal.pone.0273578)
Supplement: S2 Appendix — (PDF) [file pone.0273578.s002.pdf]

MOOSE Checklist for Meta-analyses of Observational Studies

Item No	Recommendation	Reported on Page No
Reporting of background should include		
1	Problem definition	5
2	Hypothesis statement	6 (objectives)
3	Description of study outcome(s)	5; 8
4	Type of exposure or intervention used	5; 8
5	Type of study designs used	6
6	Study population	5; 6
Reporting of search strategy should include		
7	Qualifications of searchers (eg, librarians and investigators)	7
8	Search strategy, including time period included in the synthesis and key words	6-7; Supp Table S1
9	Effort to include all available studies, including contact with authors	7
10	Databases and registries searched	6-7
11	Search software used, name and version, including special features used (eg, explosion)	7
12	Use of hand searching (eg, reference lists of obtained articles)	7
13	List of citations located and those excluded, including justification	Fig 1; Supp Table S2
14	Method of addressing articles published in languages other than English	NA
15	Method of handling abstracts and unpublished studies	6
16	Description of any contact with authors	7
Reporting of methods should include		
17	Description of relevance or appropriateness of studies assembled for assessing the hypothesis to be tested	7-8
18	Rationale for the selection and coding of data (eg, sound clinical principles or convenience)	7-8
19	Documentation of how data were classified and coded (eg, multiple raters, blinding and interrater reliability)	7
20	Assessment of confounding (eg, comparability of cases and controls in studies where appropriate)	8-9
21	Assessment of study quality, including blinding of quality assessors, stratification or regression on possible predictors of study results	7-8
22	Assessment of heterogeneity	8-9
23	Description of statistical methods (eg, complete description of fixed or random effects models, justification of whether the chosen models account for predictors of study results, dose-response models, or cumulative meta-analysis) in sufficient detail to be replicated	8-9
24	Provision of appropriate tables and graphics	Table1; Fig 1-4 (Supp Tables + Figs)
Reporting of results should include		
25	Graphic summarizing individual study estimates and overall estimate	Fig 2 and 3
26	Table giving descriptive information for each study included	Supp Table S13 and

		S14; Supp Fig S1-S51
27	Results of sensitivity testing (eg, subgroup analysis)	13; 15
28	Indication of statistical uncertainty of findings	12-13; Fig 2 and 3

Item No	Recommendation	Reported on Page No
Reporting of discussion should include		
29	Quantitative assessment of bias (eg, publication bias)	12; 15
30	Justification for exclusion (eg, exclusion of non-English language citations)	15
31	Assessment of quality of included studies	12; 15
Reporting of conclusions should include		
32	Consideration of alternative explanations for observed results	15-16
33	Generalization of the conclusions (ie, appropriate for the data presented and within the domain of the literature review)	13-14; 16
34	Guidelines for future research	16
35	Disclosure of funding source	17

From: Stroup DF, Berlin JA, Morton SC, et al, for the Meta-analysis Of Observational Studies in Epidemiology (MOOSE) Group. Meta-analysis of Observational Studies in Epidemiology. A Proposal for Reporting. *JAMA*. 2000;283(15):2008-2012. doi: 10.1001/jama.283.15.2008.
