# Supplementary material for: The complexity of examining laboratory-based biological markers associated with mortality in hospitalized patients during early phase of the COVID-19 pandemic: A systematic review and evidence map
Source: PLoS One. 2022 Sep 9;17(9):e0273578. doi: 10.1371/journal.pone.0273578 (PMC9462773; doi:10.1371/journal.pone.0273578)
Supplement: S3 Appendix — (DOCX) [file pone.0273578.s003.docx]

**S3 Appendix. Supplementary Materials**

**Table S1**. Search strategy for each database, including Medline, EMBASE, and Web of Science.

| **Search Strategy** | **Search Terms** |
| --- | --- |
| **Medline and EMBASE** | |
| 1. | (COVID-19 or COVID or coronavirus or nCoV or 2019-n-CoV or 2019 novel coronavirus or novel coronavirus or severe coronavirus disease).mp. |
| 2. | (Death or mortality).mp. [mp=title, abstract, original title, name of substance word, subject heading word, floating sub-heading word, keyword heading word, organism supplementary concept word, protocol supplementary concept word, rare disease supplementary concept word, unique identifier, synonyms] |
| 3. | (Lab* or hematol* or haematol* or Biological marker or biomarker or molecular marker or biochemical marker or cytokine* or chemokine* or polymerase chain reaction or nucleic acid amplification test or Albumin or Lactate dehydrogenase or alanine aminotransferase or aspartate aminotransferase or total bilirubin or creatine or cardiac troponin or D-dimer or prothrombin or procalcitonin or c-reactive protein or ferritin).mp. |
| 4. | (IFN-G or IL1-B or metabolites or genetic marker or genetic polymorphism or genetic variant, or single nucleotide polymorphism or SNP or DNA marker* or proteomics marker* or interleukin or angiotensin-converting enzyme 2 or ACE2).mp. |
| 5. | 3. or 4. |
| 6. | 1. and 2. and 5. |
| 7. | Limit 6. to English language |
| 8. | Limit 7. to dt=20201201-20210309 |
| 9. | Limit 8. to humans |
| **Web of Science** | |
| 1. | TS=(COVID-19 or COVID or nCoV or SARS-2-CoV or 2019-n-CoV or 2019 novel coronavirus or novel coronavirus or severe coronavirus disease) |
| 2. | TS=(Death or mortality) |
| 3. | TS=(Lab* or hematol* or haematol* or Biological marker or biomarker or molecular marker or biochemical marker or cytokine* or chemokine* or polymerase chain reaction or nucleic acid amplification test or Albumin or Lactate dehydrogenase or alanine aminotransferase or aspartate aminotransferase or total bilirubin or creatine or cardiac troponin or D-dimer or prothrombin or procalcitonin or c-reactive protein or ferritin) |
| 4. | TS=(IFN-G or IL1-B or metabolites or genetic marker or genetic polymorphism or genetic variant, or single nucleotide polymorphism or SNP or DNA marker* or proteomics marker* or interleukin or angiotensin-converting enzyme 2 or ACE2) |
| 5. | 4. or 3. |
| 6. | 5. and 2. and 1. |
| 7. | (5. and 2. and 1.) and LANGUAGE: (English) |

**Table S2**. Characteristics of studies chosen and not chosen for inclusion by country

| **Status** | **Sample Size** | **Number of biomarkers** | **Country** | **Start Enrollment** | **End Enrollment** | **Site** | **Country** | **General Population?** | **Extractible Data** | **Number of biomarkers** |
| --- | --- | --- | --- | --- | --- | --- | --- | --- | --- | --- |
| **Chosen** | 57 | 11 | Algiers | 22-Mar-20 | 29-Apr-20 | Issaad Hassani University Hospital in Algiers | Algiers | yes | yes | 11 |
| **Chosen** | 319 | 16 | Belgium | 03-Feb-20 | 15-Apr-20 | Jessa Hospital in Hasselt | Belgium | yes | yes | 16 |
| **Chosen** | 81 | 3 | Belgium | 12-Mar-20 | 30-Apr-20 | General Hospital in Belgium | Belgium | yes | yes | 3 |
| **Chosen** | 2054 | 13 | Brazil | 01-Mar | 19-Sep | 25 Brazilian hosptials | Brazil | yes | yes | 13 |
| **Chosen** | 506 | 13 | Brazil | 14-Mar-20 | 16-May-20 | Hospital das Clı´nicas da Faculdade de Medicina da Universidade de São Paulo | Brazil | yes | yes | 13 |
| **Chosen** | 56 | 9 | Brazil | 01-Jun-20 | 31-Jul-20 | Hospital in the South of Brazil, Curitiba, Parana | Brazil | yes | yes | 9 |
| **Chosen** | 201 | 9 | France | 17-Mar-20 | 18-Apr-20 | French geriatric hospital department | France | yes | yes | 9 |
| **Chosen** | 52 | 16 | France | 14-Mar-20 | 09-Apr-20 | Lariboisière-Fernand Widal Hospital, Assistance Publique– Hôpitaux de Paris, Université de Paris | France | yes | yes | 16 |
| **Chosen** | 50 | 7 | France | 01-Mar-20 | 30-Apr-20 | Clinique Ambroise Paré (Neuilly, France) | France | yes | yes | 7 |
| **Chosen** | 125 | 5 | Germany | 24-Feb-20 | 30-Jul-20 | University Hospital in Aachen | Germany | yes | yes | 5 |
| **Chosen** | 123 | 13 | Germany | 20-Feb | 20-Mar | Univ Hosptial Tubingen | Germany | yes | yes | 13 |
| **Chosen** | 97 | 9 | Greece | not reported | not reported | Athens Greece | Greece | yes | yes | 9 |
| **Chosen** | 38 | 22 | Greece | 22-Mar-20 | 25-Oct-20 | Evangelismos Hospital, Athens | Greece | yes | yes | 22 |
| **Chosen** | 235 | 9 | India | 11-May-20 | 28-Jun-20 | All India Institute of Medical Sciences, New Delhi | India | yes | yes | 9 |
| **Chosen** | 182 | 10 | India | 31-Mar-20 | 30-Jun-20 | LHMC, Delhi | India | yes | yes | 10 |
| **Chosen** | 108 | 14 | India | not reported | not reported | SMS Medical College, Jaipur, Rajasthan, India? (n**ot reported)** | India | yes | yes | 14 |
| **Chosen** | 108 | 7 | India | 20-Mar-20 | 08-May-20 | Safdarjung Hospital, New Delhi? (not reported) | India | yes | yes | 7 |
| Not chosen | 100 | 5 | India | 20-Jul | 20-Aug | Chigateri General Hospital, Davangere | India | yes | Yes | 5 |
| **Chosen** | 126 | 3 | Indonesia | 01-Mar-20 | 30-Jun-20 | Kandou Hospital | Indonesia | yes | yes | 3 |
| **Chosen** | 111 | 2 | Indonesia | 13-Mar-20 | 15-May-20 | Airlangga University Teaching Hospital | Indonesia | yes | yes | 2 |
| **Chosen** | 4,244 | 5 | International | 25-Feb-20 | 04-May-20 | 138 hospitals in France, Belgium, and Switzerland | International | yes | yes | 5 |
| **Chosen** | 3,062 | 12 | International | 01-Feb-20 | 15-May-20 | 33 different hospitals, spanning across three countries in southern Europe as well as the US | International | yes | yes | 12 |
| Not chosen | 639 | 0 | International | not reported | 22-Apr-20 | RISC-19-ICU registry (54 ICU in 10 countries) | International | yes | yes | 0 |
| **Chosen** | 1083 | 21 | Iran | 16-Feb-20 | 08-Apr-20 | Shohadaye Tajrish Hospital, Tehran | Iran | yes | yes | 21 |
| **Chosen** | 1061 | 24 | Iran | 20-Feb-20 | 02-Apr-20 | Daneshvari Hospital, Tehran | Iran | Yes | yes | 24 |
| **Chosen** | 459 | 19 | Iran | 30-Jan-20 | 05-Apr-20 | Shahid Modarres Hospital, Tehran | Iran | yes | yes | 19 |
| **Chosen** | 233 | 17 | Iran | 29-Feb-20 | 24-May-20 | Buali Hospital, Zahedan | Iran | yes | yes | 19 |
| Not chosen | 560 | 6 | Iran | 01-Mar-20 | 30-Apr-20 | Firoozgar Hospital, Tehran | Iran | yes | yes | 6 |
| Not chosen | 557 | 5 | Iran | 01-Mar-20 | 01-Apr-20 | 3 central hospitals affi liated to Babol University of Medical Sciences | Iran | yes | yes | 3 |
| Not chosen | 262 | 12 | Iran | 20-Feb-20 | 10-Mar-20 | tertiary care in Iran | Iran | yes | yes | 12 |
| Not chosen | 250 | 7 | Iran | 21-Apr-20 | 21-Aug-20 | Razi university hospital, Rasht | Iran | yes | yes | 7 |
| Not chosen | 225 | 13 | Iran | 20-Feb-20 | 20-Apr-20 | Shariati hospital in Tehran | Iran | yes | yes | 13 |
| Not chosen | 127 | 3 | Iran | 19-Feb-20 | 02-Mar-20 | Masih Daneshvari Hospital, Tehran | Iran | yes | yes | 3 |
| Not chosen | 100 | 10 | Iran | 20-Feb-20 | 20-Apr-20 | Shahid Labbafinejad Hospital | Iran | yes | yes | 10 |
| Not chosen | 100 | 9 | Iran | 25-Feb-20 | 12-Mar-20 | Babol University of Medical Science | Iran | yes | yes | 9 |
| Not chosen | 90 | 6 | Iran | 10-Feb-20 | 30-Mar-20 | Firoozgar Hospital, Tehran | Iran | yes | yes | 6 |
| Not chosen | 74 | 5 | Iran | 07-Mar-20 | May 12, 2020 | Farabi Hospital of Kermanshah Province | Iran | yes | yes | 5 |
| Not chosen | 71 | 5 | Iran | 18-Mar-20 | 24-May-20 | Khorshid hospital, Isfahan | Iran | yes | yes | 5 |
| Not chosen | 70 | 2 | Iran | 29-Feb-20 | 25-Apr-20 | Rasoul Akram Hospital, Tehran | Iran | yes | yes | 2 |
| Not chosen | 56 | 12 | Iran | 09-May-20 | 09-Jun-20 | Urmia University of Medical Sciences | Iran | yes | yes | 12 |
| Not chosen | 53 | 21 | Iran | 01-Mar-20 | 10-Apr-20 | Shahid Labbafi Nejad Hospital, Tehran | Iran | yes | yes | 21 |
| **Chosen** | 71 | 4 | Israel | not reported | not reported | Sheba Medical Center? (not reported) | Israel | yes | yes | 4 |
| **Chosen** | 54 | 13 | Israel | 27-Feb-20 | 30-Jul-20 | Sheba Medical Centre | Israel | yes | yes | 13 |
| **Chosen** | 426 | 12 | Italy | 21-Feb-20 | 31-May-20 | Azienda Ospedaliera - University of Padova Emergency Department (ED) | Italy | yes | yes | 13 |
| **Chosen** | 410 | 24 | Italy | 25-Feb-20 | 24-Mar-20 | San Raffaele Hospital, Milan | Italy | yes | yes | 24 |
| **Chosen** | 175 | 17 | Italy | 01-Mar-20 | 31-Mar-20 | Desio Hospital, Lombardy | Italy | yes | yes | 17 |
| **Chosen** | 144 | 24 | Italy | 01-Mar-20 | 30-Mar-20 | Valcamonica Hospital (Esine, Brescia, Lombardy, Italy) | Italy | yes | yes | 22 |
| Not chosen | 761 | 7 | Italy | 01-Mar-20 | 20-Apr-20 | 7 hospitals in northern Italy | Italy | yes | yes | 7 |
| Not chosen | 577 | 2 | Italy | 01-Feb-20 | 30-Apr-20 | (Humanitas Clinical and Research Hospital, Rozzano, Milan, Italy) | Italy | yes | yes | 6 |
| Not chosen | 523 | 1 | Italy | 01-Mar-20 | 14-Apr-20 | Humanitas Clinical and Research Hospital (Rozzano, Milan) | Italy | yes | yes | 1 |
| Not chosen | 409 | 9 | Italy | 21-Feb-20 | 31-Mar-20 | ‘Luigi Sacco’’ academic hospital in Milan | Italy | yes | yes | 5 |
| Not chosen | 407 | 12 | Italy | 01-Mar-20 | 28-Apr-20 | 3 hospitals in Northern Italy (“Maggiore della Carità” University Hospital in Novara, “Santi Antonio e Biagio e Cesare Arrigo” Hospital in Alessandria and “Sant’Andrea” Hospital in Vercelli) | Italy | yes | yes | 12 |
| Not chosen | 405 | 6 | Italy | 25-Feb-20 | 31-Mar-20 | Fondazione IRCCS Policlinico San Matteo (Pavia) | Italy | yes | yes | 6 |
| Not chosen | 373 | 12 | Italy | 22-Feb-20 | 15-May-20 | Policlinico di Monza | Italy | yes | yes | 12 |
| Not chosen | 319 | 9 | Italy | 20-Mar | 20-Apr | 5 italian covid centres | Italy | yes | yes | 9 |
| Not chosen | 275 | 3 | Italy | 25-Feb-20 | 25-Mar-20 | San Martino Policlinico Hospital | Italy | yes | yes | 3 |
| Not chosen | 212 | 4 | Italy | 27-Feb-20 | 17-Mar-20 | San Raffaele Hospital, Milan | Italy | yes | yes | 4 |
| Not chosen | 210 | 13 | Italy | 01-Mar-20 | 15-Apr-20 | ED of an urban teaching hospital, central Italy | Italy | yes | yes | 13 |
| Not chosen | 162 | 8 | Italy | 14-Mar-20 | 20-May-20 | General Hospital, Sestri Levante | Italy | yes | yes | 10 |
| Not chosen | 119 | 7 | Italy | not reported | not reported | University Hospital Sassari? (not reported) | Italy | yes | yes | 7 |
| Not chosen | 115 | 7 | Italy | 13-Mar-20 | 13-Apr-20 | Dolo Hospital, Venice | Italy | yes | yes | 7 |
| Not chosen | 109 | 6 | Italy | 30-Mar-20 | 15-May-20 | Sant’Orsola-Malpighi University Hospital in Bologna | Italy | yes | yes | 6 |
| Not chosen | 109 | 7 | Italy | 26-Feb-20 | 31-Mar-20 | Azienda Ospedaliera | Italy | yes | yes | 7 |
| Not chosen | 105 | 8 | Italy | 15-Mar-20 | 15-May-20 | University Hospital of Sassari and the Pneumology Unit of the Santissima Trinità Hospital of Cagliari, Sardinia | Italy | yes | yes | 8 |
| Not chosen | 78 | 6 | Italy | 11-Mar-20 | 27-Apr-20 | Policlinico University Hospital, Bari, Italy | Italy | yes | Yes | 6 |
| Not chosen | 69 | 11 | Italy | 01-Mar-20 | 31-Mar-20 | Urban teaching hospital in central Italy (not reported) | Italy | yes | yes | 11 |
| Not chosen | 57 | 7 | Italy | 01-Mar-20 | 30-Jun-20 | “Città della Salute e della Scienza” University Hospital (Turin, Northern Italy) | Italy | yes | yes | 7 |
| Not chosen | 49 | 9 | Italy | ? |  | an italian hospital? | Italy | yes | yes | 9 |
| Not chosen | 26 | 8 | Italy | 12-Mar-20 | 12-Apr-20 | Santa Maria delle Grazie Hospital, Naples | Italy | yes | Yes | 8 |
| **Chosen** | 2,254 | 11 | Korea | 18-Feb-20 | 02-Mar-20 | 10 hospitals in Daegu | Korea | yes | yes | 11 |
| **Chosen** | 1096 | 20 | Kuwait | 24-Feb-20 | 20-Apr-20 | Jaber Al-Ahman Al-Sabah Hospital | Kuwait | yes | yes | 20 |
| **Chosen** | 103 | 19 | Kuwait | 01-Mar-20 | 30-Apr-20 | ICU at Jaber Al-Ahmad Hospital | Kuwait | yes | yes | 19 |
| **Chosen** | 200 | 16 | Mexico | 28-Mar-20 | 30-Jun-20 | Hospital Regional de Alta Especialidad de la Peninsula de Yucatan | Mexico | yes | yes | 16 |
| **Chosen** | 164 | 10 | Mexico | 01-Apr-20 | 30-Apr-20 | 10 hospitals located in Mexico | Mexico | yes | yes | 10 |
| **Chosen** | 54 | 20 | Mexico | 23-Mar-20 | 26-Jun-20 | General Hospital of Mexico | Mexico | yes | yes | 20 |
| **Chosen** | 73 | 3 | Morocco | 21-Mar-20 | 24-Apr-20 | Cheikh Khalifa Hospital | Morocco | yes | yes | 3 |
| **Chosen** | 45 | 10 | Nigeria | 10-Apr-20 | 10-Jun-20 | Federal Medical Center, Katsina | Nigeria | yes | yes | 10 |
| **Chosen** | 36 | 2 | Norway | 10-Mar-20 | 04-May-20 | Akershus University Hospital | Norway | yes | yes | 2 |
| **Chosen** | 364 | 23 | Pakistan | ? | ? | Dow University Hospital, Karachi | Pakistan | yes | yes | 23 |
| **Chosen** | 191 | 17 | Pakistan | 27-Feb-20 | 30-Jun-20 | Dow University Hospital | Pakistan | yes | yes | 17 |
| **Chosen** | 183 | 19 | Pakistan | ? |  | Dow University Hospital | Pakistan | yes | yes | 19 |
| **Chosen** | 100 | 28 | Pakistan | 20-Mar | 20-Apr | Dow University Hospital, Karachi | Pakistan | yes | yes | 28 |
| Not chosen | 373 | 16 | Pakistan | ? |  | Dow University Hospital, Karachi | Pakistan | yes | yes | 16 |
| Not chosen | 299 | 9 | Pakistan | 24-Apr-20 | 24-Aug-20 | Shaheed Mohtarma Benazir Bhutto Institute | Pakistan | yes | yes | 9 |
| Not chosen | 238 | 8 | Pakistan | 01-Mar-20 | 30-Jun-20 | Combined Military Hospital, Peshawar Cantt | Pakistan | yes | yes | 7 |
| **Chosen** | 53 | 15 | Poland | 10-Mar-20 | 10-Jun-20 | Provincial Hospital in Tychy | Poland | yes | yes | 15 |
| **Chosen** | 50 | 8 | Poland | 12-Mar-20 | 05-Apr-20 | Jozef Strus Hospital, Poznan | Poland | yes | yes | 8 |
| **Chosen** | 80 | 20 | S Africa | 24-Mar-20 | 11-May-20 | Tygerberg Hospital, Cape Town | S Africa | yes | yes | 20 |
| **Chosen** | 768 | 14 | Saudi Arabia | 23-Mar-20 | 15-Jun-20 | Hospitalized in King Saud Medical City, Riyadh | Saudi Arabia | yes | yes | 14 |
| **Chosen** | 352 | 12 | Saudi Arabia | 20-Mar-20 | 31-May-20 | King Saud Medical City(?), Riyadh | Saudi Arabia | yes | yes | 12 |
| **Chosen** | 171 | 11 | Saudi Arabia | 01-Apr-20 | 30-Apr-20 | King Saud Univ Med centre | Saudi Arabia | yes | yes | 11 |
| **Chosen** | 289 | 21 | South Korea | 20-Feb-20 | 14-Apr-20 | Tertiary hospitals within the Daegu and Gyeongsangbuk-do area | South Korea | yes | yes | 21 |
| **Chosen** | 108 | 7 | South Korea | 18-Feb-20 | 04-Mar-20 | 4 hospitals in Daegu/Gyeongsangbuk-do Province | South Korea | yes | yes | 7 |
| **Chosen** | 106 |  | South Korea | 01-Feb-20 | Apr 10 2020 | Kyungpook National University Chilgok Hospital and Kyungpook National University Hospital in Daegu | South Korea | yes | yes | 3 |
| **Chosen** | 4,035 | 23 | Spain | beginning of pandemic | 17-Mar-20 | COVID-19@Spain is a retrospective nationwide cohort study of patients admitted to Spanish hospitals with laboratory-confirmed COVID-19 infection | Spain | yes | yes | 23 |
| **Chosen** | 2,873 | 22 | Spain | 01-Mar-20 | 30-Apr-20 | BIOCOVID-Spain (32 hospitals in 9 communities) | Spain | yes | yes | 22 |
| **Chosen** | 1968 | 15 | Spain | 02-Mar-20 | 31-May-20 | Infanta Leonor University Hosptial | Spain | yes | yes | 15 |
| **Chosen** | 1,208 | 16 | Spain | 01-Mar-20 | 24-Mar-20 | Gregorio Marañón University General Hospita | Spain | yes | yes | 15 |
| Not chosen | 2,022 | 7 | Spain | 22-Feb-20 | 11-May-20 | 63 ICUs across Spain | Spain | yes | yes | 7 |
| Not chosen | 1,549 | 12 | Spain | 01-Mar-20 | 28-May-20 | Infanta Leonor University Hospital, Madrid | Spain | yes | yes | 13 |
| Not chosen | 701 | 5 | Spain | 06-Mar-20 | 26-Mar-20 | Gregorio Marañón’ General Universitary Hospital | Spain | yes | yes | 5 |
| Not chosen | 609 | 12 | Spain | 01-Mar-20 | 30-Apr-20 | Hospital Universitario de Guadalajara | Spain | yes | yes | 12 |
| Not chosen | 607 | 9 | Spain | 10-Mar-20 | 15-Apr-20 | Hospital Universitario Quironsalud Madrid | Spain | yes | yes | 9 |
| Not chosen | 501 | 14 | Spain | 10-Mar-20 | 12-Apr-20 | Hospital Universitario 12 de Octubre, a large tertiary hospital in Madrid | Spain | yes | yes | 14 |
| Not chosen | 501 | 15 | Spain | 10-Mar-20 | 12-Apr-20 | a Madrid Hospital | Spain | yes | yes | 15 |
| Not chosen | 404 | 11 | Spain | 01-Mar-20 | 21-Apr-20 | Cantoblanco Hospitals, Madrid | Spain | yes | yes | 11 |
| Not chosen | 388 | 10 | Spain | 01-Apr-20 | 31-Jul-20 | Ankara City Hospital | Spain | yes | yes | 10 |
| Not chosen | 238 | 16 | Spain | 12-Mar-20 | 02-May-20 | 3 regional hospitals in Catalonia (hospitals of the Consorci Sanitari de l’Alt Penedès i Garraf) | Spain | yes | yes | 16 |
| Not chosen | 218 | 26 | Spain | 12-Mar-20 | 20-May-20 | Hospital San Juan de Alicante | Spain | yes | yes | 26 |
| Not chosen | 196 | 10 | Spain | 16-Mar-20 | 15-May-20 | University Hosp, Tarragona | Spain | yes | yes | 10 |
| Not chosen | 129 | 2 | Spain | 01-Feb-20 | 31-Mar-20 | Hospital del Mar, Barcelona | Spain | yes | yes | 2 |
| Not chosen | 115 | 4 | Spain | 01-Mar-20 | 31-Mar-20 | Hospital Universitario HM Puerta del Sur, Madrid | Spain | yes | yes | 4 |
| Not chosen | 101 | 8 | Spain | 26-Feb-20 | 29-Apr-20 | Costa del Sol, Hospital in Marbella | Spain | yes | yes | 8 |
| Not chosen | 99 | 20 | Spain | 01-Mar-20 | 30-Apr-20 | Santa Lucia University Hospital and Clinico Universitario Hospital, Spain | Spain | yes | yes | 20 |
| Not chosen | 72 | 28 | Spain | not reported | not reported | 8 Intensive Care Units from 6 hospitals of Canary Islands | Spain | yes | yes | 28 |
| Not chosen | 50 | 6 | Spain | 01-Apr-20 | 30-Apr-20 | Hospital General Universitario de Ciudad Rea | Spain | yes | yes | 6 |
| **Chosen** | 88 | 7 | Sudan | 20-Apr | 20-Jul | Gadarif Hospital | Sudan | yes | yes | 7 |
| **Chosen** | 102 | 7 | Sweden | 09-Apr-20 | 08-Jun-20 | Danderyd Hosptial Stockholm | Sweden | yes | yes | 7 (plus several types of thrombin measures) |
| **Chosen** | 235 | 5 | Switzerland | 13-Mar-20 | 14-Apr-20 | The University Hospitals are composed of 8 hospitals in the region, with 1800 beds | Switzerland | yes | yes | 5 |
| **Chosen** | 2,169 | 17 | Turkey | 01-Mar-20 | 30-Jun-20 | Istanbul Training and Research Hospital | Turkey | yes | yes | 17 |
| **Chosen** | 607 | 17 | Turkey | 20-Mar-20 | 20-Apr-20 |  | Turkey | yes | yes | 17 |
| **Chosen** | 336 | 20 | Turkey | 15-Mar-20 | 01-May-20 | Istanbul University | Turkey | yes | yes | 20 |
| **Chosen** | 136 | 20 | Turkey | 12-Mar-20 | 21-Apr-20 | Sisli Hamidiye Etfal Training and Research Hospital | Turkey | yes | yes | 18 |
| Not chosen | 681 | 9 | Turkey | 02-Apr-20 | 01-May-20 | Gaziosmanpasa Research and Training Hospital, University of Health Sciences, Istanbul | Turkey | yes | yes | 9 |
| Not chosen | 455 | 17 | Turkey | 10-Mar-19 | 10-Jun-20 | Turkey | Turkey | yes | yes | 17 |
| Not chosen | 397 | 7 | Turkey | 23-Mar-20 | 18-May-20 | Gulhane Training and Research Hospital, Ankara | Turkey | yes | yes | 7 |
| Not chosen | 350 | 12 | Turkey | 01-Apr-20 | 01-Jul-20 | Manisa-Merkezefendi State Hospital | Turkey | yes | yes | 10 |
| Not chosen | 349 | 5 | Turkey | 15-Mar-20 | 15-Apr-20 | "multicentre" | Turkey | yes | yes | 5 |
| Not chosen | 301 | 14 | Turkey | 11-Mar-20 | 20-Apr-20 | Istanbul Hospital | Turkey | yes | yes | 14 |
| Not chosen | 227 | 17 | Turkey | 11-Mar-20 | May 15 2020 | Not reported | Turkey | yes | yes | 19 |
| Not chosen | 215 | 7 | Turkey | 01-Apr-20 | 15-Apr-20 | Sakarya University Training and Research Hospital | Turkey | yes | yes | 5 |
| Not chosen | 177 | 16 | Turkey | 15-Mar-20 | 30-Apr-20 | Marmara University hospital, Istanbul | Turkey | yes | yes | 16 |
| Not chosen | 169 | 7 | Turkey | 01-Mar-20 | 30-Apr-20 | Sakarya University Training and Research Hospital | Turkey | yes | yes | 5 |
| Not chosen | 168 | 7 | Turkey | 16-Apr-20 | 16-Jun-20 | a hosptial in Istanbul | Turkey | yes | yes | 7 |
| Not chosen | 149 | 7 | Turkey | 01-Apr-20 | 20-May-20 | Health Sciences University, Bagcilar Training and Research Hospital, Istanbul, Turkey | Turkey | yes | yes | 9 |
| Not chosen | 103 | 15 | Turkey | 19-Mar-20 | 13-Apr-20 | ICU of Sakarya University Education and Research Hospital | Turkey | yes | yes | 15 |
| Not chosen | 50 | 16 | Turkey | 01-Apr-20 | 20-May-20 | Van Education and Research Hospital | Turkey | yes | yes | 16 |
| **Chosen** | 470 | 3 | UK | 12-Mar-20 | 19-May-20 | Royal Oldham Hospital, Greater Manchester | UK | yes | yes | 3 |
| **Chosen** | 450 | 9 | UK | first 450 admitted |  | Barnet Hospital, London | UK | yes | yes | 9 |
| **Chosen** | 391 | 21 | UK | 17-Mar-20 | 30-Jun-20 | University hospital of Wales | UK | yes | yes | 21 |
| **Chosen** | 321 | 3 | UK | 24-Jan-20 | 13-Apr-20 | University Hospital, University Hospitals Coventry and Warwickshire NHS Trust | UK | yes | yes | 3 |
| **Chosen** | 103 | 3 | Ukraine | 02-Feb-20 | 15-Sep-20 | Kiev City Hospital, Ukraine | Ukraine | yes | yes | 3 |
| **Chosen** | 6,493 | 18 | US | 13-Mar-20 | 17-Apr-20 | Mount Sinai Health System (8 hospitals and/or over 400 ambulatory practices in the New York City metropolitan area) | US | yes | yes | 18 |
| **Chosen** | 2,354 | 16 | US | 01-Mar-20 | Apr 16, 2020 | Montefore Medical Center/Albert Einstein College of Medicine | US | yes | yes | 16 |
| **Chosen** | 1,042 | 25 | US | not specified | 19-May-20 | Five hospitals in the Mass General Brigham health care system (Massachusetts General Hospital, MGH; Brigham and Women's Hospital, BWH; Newton Wellesley Hospital, NWH; Brigham and Women's Faulkner Hospital, BWFH; and North Shore Medical Center, NSMC) | US | yes | yes | 25 |
| **Chosen** | 408 | 26 | US | 01-Mar-20 | 09-Apr-20 | Interfaith Medical Center, Brooklyn, NY | US | yes | yes | 26 |
| Not chosen | 5766 | 10 | US | 07-Feb-20 | 04-May-20 | Stony Brook Hospital, NY | US | yes | yes | 10 |
| Not chosen | 3,273 | 12 | US | beginning of pandemic | 15-Apr-20 | Mount Sinai Health System | US | yes | yes | 12 |
| Not chosen | 2215 | 6 | US | 04-Mar-20 | 04-Apr-20 | ICUs of 65 hospitals | US | yes | yes | 10 |
| Not chosen | 1641 | 5 | US | 04-Mar-20 | 28-Apr-20 | 4 hospitals in the Boston, Massachusetts area (Massachusetts General Hospital, Brigham and Women’s Hospital, North Shore Medical Center, and NewtonWellesley Hospital) | US | yes | yes | 8 |
| Not chosen | 1,461 | 17 | US | 01-Mar-20 | 31-Mar-20 | Beaumont Health, an eight-hospital acute care regional health system caring for 2.2 million people across the communities within the Metro Detroit catchment area | US | yes | yes | 13 |
| Not chosen | 1,078 | 21 | US | 27-Feb-20 | 02-Apr-20 | Mount Sinai Health System | US | yes | yes | 18 |
| Not chosen | 887 | 12 | US | 01-Mar-20 | 03-Apr-20 | Columbia University Irving Medical Center and New York Presbyterian Allen Hospital | US | yes | yes | 16 |
| Not chosen | 832 | 21 | US | 04-Mar-20 | 24-Apr-20 | 4 hospitals in Maryland and Washington DC | US | yes | yes | 17 |
| Not chosen | 593 |  | US | 09-Mar-20 | 20-Apr-20 | Stony Brook University Hospital, Long Island NY | US | yes | yes | 10 |
| Not chosen | 551 | 4 | US | 01-Mar-20 | 18-Mar-20 | St Luke's Hospital, PA | US | yes | yes | 4 |
| Not chosen | 487 | 8 | US | 01-Jan-20 | 31-May-20 | NJ | US | yes | yes | 8 |
| Not chosen | 403 | 15 | US | 01-Jan-20 | 30-Apr-20 | Robert Wood Johnson Univ Hospital | US | yes | yes | 15 |
| Not chosen | 375 | 7 | US | 09-Mar-20 | 09-Apr-20 | The Bronxcare Health System hospital, Bronx NY | US | yes | yes | 7 |
| Not chosen | 319 | 12 | US | 01-Mar-20 | 01-Apr-20 | New York Inner City Hospital | US | yes | yes | 12 |
| Not chosen | 313 | 18 | US | 01-Mar-20 | 25-May-20 | St. Francis Hospital, Chicago Illinois | US | yes | yes | 18 |
| Not chosen | 268 | 4 | US | 06-Mar-20 | 05-May-20 | 6 cohorts COVID ICUs across three Emory Healthcare acute-care hospitals in Atlanta, GA | US | yes | yes | 4 |
| Not chosen | 257 | 9 | US | 20-Mar | 20-Jul | JFK medical centre, FL | US | yes | yes | 9 |
| Not chosen | 242 | 6 | US | 01-Mar-20 | 24-Apr-20 | Einstein Medical Center Philadelphia | US | yes | yes | 6 |
| Not chosen | 242 | 6 | US | 01-Mar-20 | 24-Apr-20 | a hospital in Philadelphia | US | yes | yes | 6 |
| Not chosen | 235 | 6 | US | 19-Mar-20 | 25-Apr-20 | Inner City Hospital NYC | US | yes | yes | 6 |
| Not chosen | 217 | 2 | US | 06-Mar-20 | 17-Apr-20 | Emory Healthcare acute-care hospitals in Atlanta | US | yes | yes | 2 |
| Not chosen | 202 | 9 | US | 15-Mar-20 | 15-Jul-20 | 2 Boston academic centres | US | yes | yes | 9 |
| Not chosen | 200 | 26 | US | 01-Mar-20 | 30-May-20 | An academic inner-city hospital in Wasington DC | US | yes | yes | 26 |
| Not chosen | 143 | 4 | US | 15-Mar-20 | 30-Apr-20 | Cedars-Sinai Medical Center, Los Angeles, California | US | yes | yes | 4 |
| Not chosen | 140 | 9 | US | 01-Mar-20 | 31-Mar-20 | Indiana: Large, academic, Midwestern institution which serves as a referral center for the State of Indiana | US | yes | yes | 9 |
| Not chosen | 80 | 6 | US | 01-Feb-20 | 31-May-20 | 4 hospitals in El Paso, Texas | US | yes | yes | 6 |
| Not chosen | 70 | 26 | US | 02-Mar-20 | 23-Apr-20 | State University of New York (SUNY), Downstate Medical Center, Brooklyn, NY | US | yes | yes | 26 |
| Not chosen | 41 | 44 | US | 20-Mar-20 | 17-Apr-20 | Albany Medical Centre | US | yes | yes | 44 |
| Not chosen | 33 | 1 | US | not reported | not reported | University of Miami Hospital | US | yes | yes | 1 |
| Not chosen | 22 | 23 | US | 28-Mar-20 | 27-Jun-20 | University of Alabama Brigham Young | US | yes | yes | 23 |

**Table S3**. List of all biomarkers extracted from studies according to clinical category.

| Clinical Category | Biomarker |
| --- | --- |
| Anemia | Erythrocytes |
|  | Ferritin |
|  | Hemoglobin |
|  | Transferrin |
| Cardiac | α-hyroxybutyric dehydrogenase (HBDH) |
|  | Cholesterol |
|  | Creatine kinase |
|  | High density lipoprotein (HDL) |
|  | Low density lipoprotein (LDL) |
|  | Myoglobin |
|  | N-terminal pro-B-type natriuretic peptide/BNP (NT-proBNP) |
|  | Triglyceride |
|  | Troponin |
| Coagulation | Activated partial thromboplastin time (APTT) |
|  | D-dimer |
|  | Fibrin-degradation products (FDP) |
|  | Fibrinogen |
|  | Platelet |
|  | Prothrombin time |
|  | Thromobocytocrit (aka plateletcrit) |
| Hepatic | Alanine aminotransferase (ALT) |
|  | Albumin |
|  | Aspartate aminotransferase (AST) |
|  | Bilirubin |
|  | Cholinesterase |
|  | γ-glutamyl (γ-GT) |
|  | Lactate dehydrogenase (LDH) |
| Inflammation | Amyloid A |
|  | Basophil |
|  | Complement protein: C3 |
|  | Complement protein: C4 |
|  | C-reactive protein |
|  | Eosinophil |
|  | Eotaxin |
|  | Erythrocyte sedimentation rate (ESR) |
|  | FGF Basic |
|  | G-CSF |
|  | GM-CSF |
|  | IFN-γ |
|  | IFN-γ+ CD4+ T cells /Th % |
|  | IFN-γ+ CD8+ T cells /Ts % |
|  | IFN-γ+ NK cells /NK % |
|  | IL-10 |
|  | IL-12 (p70) |
|  | IL-13 |
|  | IL-15 |
|  | IL-17 |
|  | IL-1b |
|  | IL-1ra |
|  | IL-1β |
|  | IL-2 |
|  | IL-2R |
|  | IL-4 |
|  | IL-5 |
|  | IL-6 |
|  | IL-7 |
|  | IL-8 |
|  | IL-9 |
|  | Immunoglobulin A |
|  | Immunoglobulin G |
|  | Immunoglobulin M |
|  | IP10 |
|  | Leucocyte |
|  | Lymphocyte |
|  | Macrophage inflammatory protein - 1A |
|  | Macrophage inflammatory protein - 1B |
|  | MCP-1 |
|  | Monocyte |
|  | Neutrophil |
|  | Neutrophil-to-lymphocyte ratio (NLR) |
|  | PDGF |
|  | Platelet to Leucocyte Ratio (PLR) |
|  | Preaalbumin (transythretin) |
|  | Procalcitonin |
|  | RANTES |
|  | T cells/B cells: Activated T cells (CD3+HLA-DR+) /ul |
|  | T cells/B cells: Activated Ts cells (CD3+CD8+HLA-DR+)/Ts % |
|  | T cells/B cells: CD28+ Th cells (CD3+CD4+CD28+)/Th % |
|  | T cells/B cells: CD28+ Ts cells (CD3+CD8+CD28+)/Ts % |
|  | T cells/B cells: CD3 |
|  | T cells/B cells: CD4 |
|  | T cells/B cells: CD4/CD8 |
|  | T cells/B cells: CD8 |
|  | T cells/B cells: Induced regulatory T cells (CD45RO+CD3+CD4+CD25+CD127low+) |
|  | T cells/B cells: Memory Th cells (CD3+CD4+CD45RO+)/Th |
|  | T cells/B cells: Naïve regulatory T cells (CD45RA+CD3+CD4+CD25+CD127low+) |
|  | T cells/B cells: Naïve Th cells (CD3+CD4+CD45RA+)/Th |
|  | T cells/B cells: Regulatory T cells (CD3+CD4+CD25+CD127low+) |
|  | T cells/B cells: T cells (CD3+CD19-) |
|  | T cells/B cells: T cells+B cells+NK cells |
|  | T cells/B cells: Th cells (CD3+CD4+) |
|  | T cells/B cells: Th/Ts |
|  | T cells/B cells: Total T cell count |
|  | T cells/B cells: Ts cells (CD3+CD8+) |
|  | TNF-α |
|  | VEGF |
|  | Globulin |
|  | White blood cell count (WBC) |
| Metabolic | Calcium |
|  | Chloride |
|  | Glucose |
|  | Bicarbonate (HCO3) |
|  | Lactate |
|  | Lactic acid |
|  | Potassium |
|  | Sodium |
|  | Total protein |
| Pulmonary | Oxyhemoglobin saturation |
|  | Partial pressure of oxygen (PaO2) |
|  | PaO2/FiO2 |
|  | SpO2 |
| Renal | Creatinine |
|  | Cystatin C |
|  | Estimated glomerular filtration rate (eGFR) |
|  | Urea/blood urea nitrogen (BUN) |

**Table S4.** Proportion of anemia biomarkers reported across included studies comparing survivors and non-survivors (n=93).

| **Anemia Biomarkers (4 biomarkers)** | **Frequency** | **% of Studies** |
| --- | --- | --- |
| Hemoglobin | 51 | 54.3 |
| Ferritin | 35 | 37.2 |
| Erythrocytes | 0 | 0 |
| Transferrin | 0 | 0 |

**Table S5.** Proportion of cardiac biomarkers reported across included studies comparing survivors and non-survivors (n=93).

| **Cardiac Biomarkers (10 biomarkers)** | **Frequency** | **% of Studies** |
| --- | --- | --- |
| Troponin | 37 | 39.4 |
| Creatine kinase | 30 | 31.9 |
| N-terminal pro-B-type natriuretic peptide (NT-proBNP)/ BNP | 17 | 18.1 |
| Creatine kinase myocardial band (CK-MB) | 7 | 7.4 |
| Cholesterol | 4 | 4.3 |
| Triglyceride | 4 | 4.3 |
| Myoglobin | 4 | 4.3 |
| HDL | 1 | 1.1 |
| LDL | 1 | 1.1 |
| α-hydroxybutyric dehydrogenase (HBDH) | 1 | 2.4 |

**Table S6.** Proportion of coagulation biomarkers reported across included studies comparing survivors and non-survivors (n=93).

| **Coagulation Biomarkers (7 biomarkers)** | **Frequency** | **% of Studies** |
| --- | --- | --- |
| Platelet | 60 | 63.8 |
| D-dimer | 59 | 52.8 |
| Prothrombin time (PT) | 27 | 28.7 |
| Activated partial thromboplastin time (APTT) | 17 | 18.1 |
| Fibrinogen | 16 | 63.8 |
| Fibrin-degradation products (FDP) | 0 | 0 |
| Thromobocytocrit (aka plateletcrit) | 0 | 0 |

**Table S7.** Proportion of hepatic biomarkers reported across included studies comparing survivors and non-survivors (n=93).

| **Hepatic Biomarkers (7 biomarkers)** | **Frequency** | **% of Studies** |
| --- | --- | --- |
| Alanine aminotransferase (ALT) | 52 | 55.3 |
| Lactate dehydrogenase (LDH) | 52 | 55.3 |
| Aspartate aminotransferase (AST) | 48 | 51.1 |
| Bilirubin | 34 | 36.2 |
| Albumin | 31 | 33.0 |
| γ-glutamyl (GT) | 9 | 9.6 |
| Cholinesterase | 0 | 0 |

**Table S8.** Proportion of inflammatory biomarkers reported across included studies comparing survivors and non-survivors (n=93).

| **Inflammatory Biomarkers (71 biomarkers)** | **Frequency** | **% of Studies** |
| --- | --- | --- |
| lymphocyte | 72 | 76.6 |
| C-reactive protein (and hypersensitive/high sensitivity CRP) | 69 | 73.4 |
| white blood cell | 54 | 57.4 |
| neutrophil | 49 | 52.1 |
| procalcitonin | 43 | 47.5 |
| neutrophil-to-lymphocyte ratio (NLR) | 22 | 23.4 |
| IL-6 | 21 | 22.3 |
| Erythrocyte sedimentation rate (ESR) | 15 | 16.0 |
| Monocyte | 13 | 13.8 |
| Leukocyte | 12 | 12.8 |
| TNF-α | 9 | 9.6 |
| IL-10 | 6 | 6.4 |
| T cells/B cells: CD3 | 6 | 6.4 |
| T cells/B cells: CD4 | 6 | 6.4 |
| T cells/B cells: CD8 | 5 | 5.3 |
| T cells/B cells: CD4/CD8 | 4 | 4.3 |
| IFNγ | 4 | 4.3 |
| IL-8 | 4 | 4.3 |
| IL-4 | 4 | 4.3 |
| Eosinophil | 3 | 3.2 |
| IL-1b | 3 | 3.2 |
| Amyloid A | 2 | 2.1 |
| Basophil | 2 | 2.1 |
| Globulin | 2 | 2.1 |
| IL-2 | 2 | 2.1 |
| IL-2R | 2 | 2.1 |
| T cells/B cells: Th cells (CD3+CD4+) | 2 | 2.1 |
| T cells/B cells: Ts cells (CD3+CD8+) | 2 | 2.1 |
| Compliment protein 3 | 1 | 1.1 |
| Compliment protein 4 | 1 | 1.1 |
| IFN-γ+ CD4+ T cells /Th % | 1 | 1.1 |
| IL-17 | 1 | 1.1 |
| Immunoglobin-A | 1 | 1.1 |
| Immunoglobin-G | 1 | 1.1 |
| Immunoglobin-M | 1 | 1.1 |
| Eotaxin | 0 | 0 |
| FGF basic | 0 | 0 |
| G-CSF | 0 | 0 |
| GM-CSF | 0 | 0 |
| IFN-γ+ CD8+ T cells /Ts % | 0 | 0 |
| IFN-γ+ NK cells /NK % | 0 | 0 |
| IL-12 (p70) | 0 | 0 |
| IL-13 | 0 | 0 |
| IL-15 | 0 | 0 |
| IL-1ra | 0 | 0 |
| IL-1β | 0 | 0 |
| IL-5 | 0 | 0 |
| IL-7 | 0 | 0 |
| IL-9 | 0 | 0 |
| IP-10 | 0 | 0 |
| Macrophage inflammatory protein - 1A (MCP1A) | 0 | 0 |
| Macrophage inflammatory protein - 1B (MCP1B) | 0 | 0 |
| MCP-1 | 0 | 0 |
| PDGF (Platelet-derived growth factor) | 0 | 0 |
| Platelet-to-leucocyte ratio (PLR) | 0 | 0 |
| Prealbumin (transythretin) | 0 | 0 |
| RANTES | 0 | 0 |
| T cells/B cells: Activated T cells (CD3+HLA-DR+) /ul | 0 | 0 |
| T cells/B cells: Activated Ts cells (CD3+CD8+HLA-DR+)/Ts % | 0 | 0 |
| T cells/B cells: CD28+ Th cells (CD3+CD4+CD28+)/Th % | 0 | 0 |
| T cells/B cells: CD28+ Ts cells (CD3+CD8+CD28+)/Ts % | 0 | 0 |
| T cells/B cells: Induced regulatory T cells (CD45RO+CD3+CD4+CD25+CD127low+) | 0 | 0 |
| T cells/B cells: Memory Th cells (CD3+CD4+CD45RO+)/Th | 0 | 0 |
| T cells/B cells: Regulatory T cells (CD3+CD4+CD25+CD127low+) | 0 | 0 |
| T cells/B cells: Naïve regulatory T cells (CD45RA+CD3+CD4+CD25+CD127low+) | 0 | 0 |
| T cells/B cells: Naïve Th cells (CD3+CD4+CD45RA+)/Th | 0 | 0 |
| T cells/B cells: T cells (CD3+CD19-) | 0 | 0 |
| T cells/B cells: T cells+B cells+NK cells | 0 | 0 |
| T cells/B cells: Th/Ts | 0 | 0 |
| T cells/B cells: Total T cell count | 0 | 0 |
| VEGF (Vascular endothelial growth factor) | 0 | 0 |

**Table S9.** Proportion of metabolic biomarkers reported across included studies comparing survivors and non-survivors (n=93).

| **Metabolic Biomarkers (8 biomarkers)** | **Frequency** | **% of Studies** |
| --- | --- | --- |
| Sodium | 26 | 27.7 |
| Potassium | 24 | 25.5 |
| Glucose | 17 | 18.1 |
| Lactate | 13 | 13.8 |
| Total protein | 8 | 8.5 |
| Chloride | 6 | 6.4 |
| Bicarbonate/ HCO_3_^-^ | 5 | 5.3 |
| Calcium | 4 | 4.3 |
| Lactic acid | 1 | 1.1 |

**Table S10.** Proportion of pulmonary biomarkers reported across included studies comparing survivors and non-survivors (n=93).

| **Pulmonary Biomarkers (4 biomarkers)** | **Frequency** | **% of Studies** |
| --- | --- | --- |
| PaO2 (partial pressure oxygen) | 5 | 5.3 |
| PaO2/FiO2 | 4 | 4.3 |
| SpO2 (percutaneous oxygen saturation) | 3 | 3.2 |
| Oxyhemoglobin saturation | 2 | 2.1 |

**Table S11.** Proportion of renal biomarkers reported across included studies comparing survivors and non-survivors (n=93).

| **Renal Biomarkers (4 biomarkers)** | **Frequency** | **% of Studies** |
| --- | --- | --- |
| Creatinine | 66 | 70.2 |
| Urea | 41 | 43.6 |
| Estimated glomerular filtration rate (eGFR) | 11 | 11.7 |
| Cystatin C | 1 | 1.1 |

**Table S12.** Summary of biomarkers reported across included studies by category of biomarker (n=94).

| **Biomarker Category (# of biomarkers considered)** |  |
| --- | --- |
| **Anemia** (4 biomarkers) |  |
| Mean (SD) | 0.9 (0.8) |
| Median (IQR) [range] | 1 (0-2) [0-2] |
| Studies reporting any anemia biomarkers, N (%) | 59 (62.8) |
| **Cardiac** (10 biomarkers) |  |
| Mean (SD) | 1.1 (1.4) |
| Median (IQR) [range] | 1 (0-2) [2-6] |
| Studies reporting any cardiac biomarkers, N (%) | 48 (51.1) |
| **Coagulation** (7 biomarkers) |  |
| Mean (SD) | 1.9 (1.4) |
| Median (IQR) [range] | 2 (1-3) [0-5] |
| Studies reporting any coagulation biomarkers, N (%) | 79 (84.0) |
| **Hepatic** (7 biomarkers) |  |
| Mean (SD) | 2.4 (1.9) |
| Median (IQR) [range] | 2 (1-4) [0-6] |
| Studies reporting any hepatic biomarkers, N (%) | 74 (78.7) |
| **Inflammatory** (71 biomarkers) |  |
| Mean (SD) | 4.7 (3.6) |
| Median (IQR) [range] | 4 (2-6.75) [0-17] |
| Studies reporting any inflammatory biomarkers, N (%) | 86 (91.5) |
| **Metabolic** (9 biomarkers) |  |
| Mean (SD) | 1.1 (1.5) |
| Median (IQR) [range] | 0 (0-2) [0-6] |
| Studies reporting any metabolic biomarkers, N (%) | 45 (47.9) |
| **Pulmonary** (4 biomarkers) |  |
| Mean (SD) | 0.15 (0.44) |
| Median (IQR) [range] | 0 (0-0) [0-2] |
| Studies reporting any pulmonary biomarkers, N (%) | 11 (11.7) |
| **Renal** (4 biomarkers) |  |
| Mean (SD) | 1.3 (0.9) |
| Median (IQR) [range] | 1 (1-2) [0-3] |
| Studies reporting any renal biomarkers, N (%) | 72 (76.6) |

**Table S13. Characteristic of Included Studies**

| Author, year | Setting | Study Sample | Study Start/End Date | Method of Diagnosis |
| --- | --- | --- | --- | --- |
| Abdeladim, 2020^1^ | Morocco | 38.4% male  Mean age: 54.58 years  (N=73) | 21/03/2020 - 24/04/2020 | Real-time fluorescence polymerase chain reaction of COVID-19 virus RNA nucleic acid test |
| Abohamr, 2020^2^ | Saudi Arabia | 76.6% male  Mean age 46.36 years  (N=768) | 23/03/2020 - 15/06/2020 | PCR test from nasopharyngeal swab sample |
| Alamdari, 2020^3^ | Iran | 69.7% male  Mean age: 61.79 years  (N=459) | 30/01/2020 - 05/04/2020 | RT-PCR |
| Alharthy, 2020^4^ | Saudi Arabia | 78.9% male  Mean age: 47 years  (N=171) | 01/04/2020 - 30/04/2020 | RT-PCR assays on throat swab samples |
| Alharthy, 2020^5^ | Saudi Arabia | 87.2% male  Mean age: 50.63 years  (N=352) | 20/03/2020 - 31/05/2020 | RT-PCR assays performed on nasopharyngeal swabs |
| Almazeedi, 2020^6^ | Kuwait | 81% male  Median age: 41 years  Ethnicity: 48.1% South East Asian, 27.1% Kuwaitis, 6.6% Egyptians  (N=1096) | 24/02/2020 - 20/04/2020 | RT-PCR assay of specimens obtained via nasopharyngeal swabs |
| Altschul, 2020^7^ | US | 48.6% male  Mean age: 67.64 years  Ethnicity: 42.2% Black, 35.0% Hispanic, 11.9% White, 2.2% South East Asian  (N=2354) | 01/03/2020 - 16/04/2020 | RT-PCR assay testing |
| Arifputra, 2020^8^ | Indonesia | 47.6% male  Mean age: 48.81 years  (N=126) | 01/03/2020 - 30/06/2020 | RT-PCR |
| Asghar, 2021^9^ | Pakistan | 68.8% male  Mean age: 52.75 years  (N=183) | NR | Nasopharyngeal or oropharyngeal swab for RT-PCR |
| Asghar, 2020^10^ | Pakistan | 67% male  Mean age: 52.65 years  (N=191) | 27/02/2020 - 30/06/2020 | Nasopharyngeal swab (PCR), Oropharyngeal swab (PCR) |
| Asghar, 2020^11^ | Pakistan | 67.6% male  Mean age: 52.6 years  (N=364) | NR | NR |
| Asghar, 2020^12^ | Pakistan | 69% male  Mean age 52.58 years  (N=100) | 01/03/2020 - 30/04/2020 | nasopharyngeal or oropharyngeal swab for PCR |
| Ayed, 2020^13^ | Kuwait | 85.4% male  Median age: 53 years  (N=103) | 01/03/2020 - 30/04/2020 | RT-PCR assay of nasopharyngeal swab specimens |
| Balfanz, 2020^14^ | Germany | 70% male  Mean age: 66 years  (N=125) | 24/02/2020 - 30/07/2020 | SARs-CoV-2 respiratory samples |
| Bannaga, 2020^15^ | UK | 58.9% male  Median age: 73 years  Ethnicity: 77.0% White, 23.0% Other  (N=321) | 24/01/2020 - 13/04/2020 | positive result for SARS-CoV-2 on RT-PCR assay of either nasal and pharyngeal  swab or sputum specimens |
| Barman, 2020^16^ | Turkey | 55% male  Mean age: 63.4 years  (N=607) | 20/03/2020 - 20/04/2020 | RT-PCR |
| Belaid, 2021^17^ | Algeria | 70.18% male  Mean age: 59.72 years  (N=57) | 22/03/2020 - 29/04/2020 | Nucleic acid in nasopharyngeal swab samples using a real-time RT-PCR test |
| Berenguer, 2020^18^ | Spain | 61.0% male  Median age: 70 years  Ethnicity: 93% White, 4.7% Hispanic  (N=4035) | 17/03/2020 - 17/04/2020 | RT-PCR assay |
| Bertsimas, 2020^19^ | International | 67.2% male  Mean age: 68 years  (N=3032) | 01/02/2020 - 15/05/2020 | polymerase chain  reaction testing of nasopharyngeal samples |
| Bonetti, 2020^20^ | Italy | 66.7% male  Mean age: survivors 62.1 years; non-survivors 78.0 years  (N=144) | 01/03/2020 - 30/03/2020 | Displaying suggestive findings at chest computed tomography (CT; the classic ground glass pattern of interstitial pneumonia for a minimum of 35%–40% of lung parenchyma) and RT-PCR for SARS-CoV-2 |
| Brandao Neto, 2021^21^ | Brazil | 47.3% male  Mean age: 60.1 years  (N=506) | 14/03/2020 - 16/05/2020 | RT-PCR on nasopharyngeal swabs or tracheal aspirate specimens |
| Brill, 2020^22^ | UK | 60% male  Median age: 72 years  Ethnicity: 59.0% White, 11.0% South East Asian, 7.0% Black, 17.0% other, 5.0% NR  (N=450) | 10/03/2020 - 08/04/2020 | Inpatients returning consecutive positive PCR tests for SARS-CoV2 on nasopharyngeal swabs during their hospital admission were included for analysis |
| Chang, 2020^23^ | South Korea | 50.1% male  Mean age: 67.6 years  (N=106) | 01/02/2020 - 10/04/2020 | RT-PCR assay for SARS-CoV-2 in upper respiratory specimens (nasopharyngeal and oropharyngeal swab), with or without a lower respiratory specimen (sputum) |
| Chen, 2020^24^ | China | 62% male  median age: 62 years  (N=799) | 13/01/2020 - 28/02/2020 | RT-PCR assay of SARS-Cov-2 RNA |
| Chen, 2020^25^ | China | 50% male  Median age: 59 years  (N=1859) | 20/01/2020 - 04/05/2020 | Clinical symptoms and lung CT consistent with COVID-19 followed by confirmation of SARS-CoV-2- infection by qRT-PCR. AntiSARS-CoV-2 IgM and/or IgG antibodies were assayed in qRT-PCR-negative patients |
| Ciceri, 2020^26^ | Italy | 72.9% male  Median age: 65 years  Ethnicity: 93.2% Euro, 5.6% Chinese, 1.2% Hispanic  (N=410) | 25/02/2020 - 24/03/2020 | RT-PCR from a nasal and/or throat swab together with signs, symptoms, or radiological findings suggestive of COVID-19 pneumonia |
| Cortes-Telles, 2020^27^ | Mexico | 69% male  Median age: 55 years  (N=200) | 28/03/2020 - 30/06/2020 | RT_PCR using nasal and pharyngeal swabs specimens |
| COVID-ICU Group on behalf of the REVA Network and the COVID-ICU Investigators, 2020^28^ | 138 hospitals in France, Belgium, and Switzerland | 74% male  Mean age: 63 years  (N=4244) | 25/02/2020 - 04/05/2020 | RT-PCR assay from either nasal or pharyngeal swabs, or lower respiratory tract aspirates |
| Cuvelier, 2020^29^ | France | 74% male  Mean age: 63.2 years  (N=50) | 01/03/2020 - 30/04/2020 | nasal viral RNA detection using reverse transcriptase polymerase chain reaction (RT-PCR) |
| Deng, 2020^30^ | China | 67% male in death group, 44% male in recovered group;  median age death group: 69 years, median age recovered group: 40 years  (N=225) | 01/01/2020 –  21/02/2020 | real-time reverse transcription polymerase chain reactions |
| De Michieli, 2021^31^ | Italy | 57.7% male  Mean age: 64.1 years  (N=426) | 21/02/2020 - 31/05/2020 | RT-PCR from nasopharyngeal swabs |
| De Smet, 2020^32^ | Belgium | 41% male  Median age: 85 years  (N=81) | 12/03/2020 - 30/04/2020 | RT-PCR testing on nasopharyngeal swabs |
| Du, 2020^33^ | China | 54.2% male  Mean age: 57.6 years  (N=179) | 25/12/2019 - 07/02/2020 | The probable and definite diagnosis of COVID-19 pneumonia was established according to the case definition established by WHO interim guidance |
| Gadotti, 2020^34^ | Brazil | 69.6% male  Median age: 61 years  (N=56) | 01/06/2020 - 31/07/2020 | COVID-19 infection was defined by clinical-radiological presentation plus a nasopharyngeal swab PCR positive to COVID-19 |
| Garcia de Guadiana-Romualdo, 2021^35^ | Spain | 59.1% male  Median age: 66 years  (N=2873) | 01/03/2020 - 30/04/2020 | RT-PCR testing of a nasopharyngeal specimen, or by a positive result of serological testing and a clinically compatible presentation |
| Gayam, 2021^36^ | US | 57% male  Mean age: 67 years  Ethnicity: 100% Black | 01/03/2020 - 09/04/2020 | Nasopharyngeal swabs, RT-PCR test |
| Genet, 2020^37^ | France | 32.8% male  Mean age: 86.3 years  (N=201) | 17/03/2020 - 18/04/2020 | RTPCR from nasal swabs |
| Guo, 2020^38^ | China | 48.7% male  Mean age: 58.5 (14.66) years  (N=187) | 23/01/2020 - 23/02/2020 | RT-PCR |
| Gupta, 2020^39^ | India | 58% male  Mean age: 40.03 years  (N=108) | 20/03/2020 - 08/05/2020 | Oro-nasopharyngeal swab-based testing using RT-PCR |
| Gurtoo, 2020^40^ | India | 59% male  Mean age: 46.1 years  (N=182) | 31/03/2020 - 30/06/2020 | NR |
| Huang, 2020^41^ | China | 53.5% male  Mean age: 53.4 (16.7) years  (N=299) | 25/01/2020 - 24/03/2020 | RT‐PCR assay from naso‐pharyngeal swab specimens |
| Kayina, 2020^42^ | India | 68.1% male  Mean age: 50.7 years  (N=235) | 11/05/2020 - 28/06/2020 | RT-PCR of the nasopharyngeal and oropharyngeal swabs |
| Khoshnood, 2020^43^ | Iran | 61.9% male  Mean age: 50.75 years  (N=1086) | 16/02/2020 - 08/04/2020 | RT-PCR |
| Kim, 2021^44^ | Korea | 35.8% male  Mean age: 58 years  (N=2254) | 18/02/2020 - 10/07/2020 | RT–PCR assays performed on nasopharyngeal swab specimens |
| Kokoszka-Bargiel, 2020^45^ | Poland | 69% male  Mean age: 62.4 years  (N=32) | 10/03/2020 - 10/06/2020 | RT-PCR assay from nasal and pharyngeal swabs or lower respiratory tract aspirates |
| Kunal, 2020^46^ | India | 64.8% male  Mean age: 51.2 years  (N=108) | NR | RT-PCR assay |
| Lee, 2020^47^ | South Korea | 44.9% male  Median age: 72 years  (N=98) | 18/02/2020 - 04/03/2020 | RT-PCR assay of a nasopharyngeal swab or sputum |
| Li, 2020^48^ | China | 56.8% male  Mean age: 62.05 (12.68) years  (N=132) | 18/01/2020 - 26/02/2020 | Sputum and throat swab specimens collected from all patients at admission were tested by RT-PCR for SARS-Cov-2 RNA |
| Li, 2020^49^ | China | 51% male  Median age: 57 years  (N=1449) | 20/01/2020 - 04/04/2020 | qRTPCR of nasal and pharyngeal swabs and/or blood test for anti-SARS-CoV-2 IgG/IgM antibodies using a colloidal gold-based 2019-nCoV IgG/IgM Detection Kit |
| Mandel, 2020^50^ | Israel | 73.2% male  Mean age: 62.0 years  (N=71) | NR | nasopharyngeal test |
| Marcolino, 2021^51^ | Brazil | 52.6% male  Median age: 59 years  (N=2054) | 01/03/2020 - --/09/2020 | RT-PCR tests on nasopharyngeal or oropharyngeal swabs. |
| Mendes, 2020^52^ | Switzerland | 43% male  Mean age: 86 years  Ethnicity: 100% White  (N=235) | 13/03/2020 - 14/04/2020 | RT-PCR using nasopharyngeal swabs |
| Mikami, 2021^53^ | US | 54.5% male  Mean age: 59 years  Ethnicity: 44.7% other, 26.9% White, 25.4% Hispanic, 24.1% Black, 4.4% South East Asian  (N=2820) | 12/03/2020 - 17/04/2020 | RT-PCR assay of nasopharyngeal swab specimens |
| Namendys-Silva, 2020^54^ | Mexico | 69.5% male  Mean age: 57.3 years  (N=164) | 01/04/2020 - 30/04/2020 | Polymerase chain reaction testing of nasopharyngeal specimens |
| Neumann-Podczaska, 2020^55^ | Poland | 70% male  Mean age: 74.8 years  (N=50) | 12/03/2020 - 05/04/2020 | RT-PCR testing of a nasopharyngeal swab |
| Nicholson, 2021^56^ | US | 56.8% male  Median age: 64 years  Ethnicity: 42.0% White, 17.9% Black, 10.8% Hispanic, 3.6% South East Asian, 17.0% other, 8.6% NR  (N=1042) | NR - 19/05/2020 | RT-PCR assay of a specimen collected on a nasopharyngeal swab |
| Omar, 2020^57^ | Sudan | 81.8% male  Median age: 62 years  (N=88) | --/04/2020 - --/07/2020 | Laboratory testing using PCR |
| Oliynyk, 2020^58^ | Ukraine | 50.6% male  Mean age: 69.15 years  (N=103) | 02/02/2020 - 15/09/2020 | reverse transcription polymerase chain reaction |
| Park, 2020^59^ | South Korea | 46% male  Median age: 72 years  (N=289) | 20/02/2020 - 14/04/2020 | RT-PCR from nasopharyngeal and oropharyngeal swabs |
| Parker, 2020^60^ | South Africa | 39% male  Mean age: 48 years  (N=80) | 24/03/2020 - 11/05/2020 | SARS-CoV-2-positive PCR result and radiological evidence  compatible with COVID-19 |
| Ponsford, 2021^61^ | UK | 52.4% male  Median age: 69 years  (N=391) | 17/03/2020 - 30/06/2020 | PCR on nasopharyngeal swab |
| Rath, 2020^62^ | Germany | 62.6% male  Mean age: 68.0 years  (N=123) | --/02/2020 - --/03/2020 | RNA detection from nasopharyngeal secretions with real-time reverse transcriptase polymerase chain reaction |
| Rizo-Tellez, 2020^63^ | Mexico | 75% male  Mean age: 62.9 years  Ethnicity: 100% Hispanic  (N=54) | 23/03/2020 - 26/06/2020 | Nasopharyngeal swabs using quantitative polymerase chain reaction (qPCR) |
| Rodriguez-Gonzalez, 2020^64^ | Spain | NR | 01/03/2020 - 24/03/2020 | PCR test |
| Rokni, 2020^65^ | Iran | 63.9% male  Mean age: 49.8 years  (N=233) | 29/02/2020 - 24/05/2020 | RT-PCR assay of naso-pharyngeal or oro-pharyngeal swab specimens |
| Sun, 2020^66^ | China | 54.5% male  Mean age: discharged 67 years (64-72); deceased 72 years (66-78)  (N=244) | 29/01/2020 - 03/05/2020 | Diagnosis of all included patients was confirmed according to the Diagnosis and Treatment Guideline for COVID‐19 published by the National Health Commission of Peopleʼs Republic of China |
| Schwarz, 2021^67^ | Israel | Survivors 70.5% male  Non-survivors 60% male  Mean age: survivors 55.05 years; non-survivors 74.93 years  (N=54) | 27/02/2020 - 30/07/2020 | polymerase chain reaction (PCR) |
| Sensusiati, 2021^68^ | Indonesia | 48.6% male  Mean age: 50 years  (N=111) | 13/03/2020 - 15/05/2020 | RT PCR using nasopharyngeal and oropharyngeal specimens |
| Serin, 2020^69^ | Turkey | 53% male  Mean age: 47.7 years  (N=2217) | 01/03/2020 - 30/06/2020 | nasopharyngeal PCR swab |
| Shi, 2020^70^ | China | 49% male  Median age: 65 years  (N=153) | 01/01/2020 - 08/03/2020 | Throat swabs using RT-PCR |
| Sovik, 2020^71^ | Norway | 75.0% male  Mean age: 61 years  (N=36) | 10/03/2020 - 04/05/2020 | SARS-CoV-2 RT-PCR nasopharyngeal swab |
| Sulejmani, 2021^72^ | Italy | 69.7% male  Mean age: 71.0 years  (N=175) | 01/03/2020 - 31/03/2020 | RT–PCR assays of nasopharyngeal swabs |
| Sweeney, 2020^73^ | Greece | 68% male  Mean age: 62 years  (N=97) | NR | PCR testing |
| Tahtasakal, 2021^74^ | Turkey | 56.4% male  Median age: 66 years  (N=136) | 12/03/2020 - 21/04/2020 | Patients were detected with SARS‐CoV2 RT‐PCR (+) and/or SARS‐CoV2 Ig M/IgG (+) or who have the symptoms that are radiologically compatible with COVID‐19 but could not be explained by other factors were included in the study |
| Tang, 2020^75^ | China | 59.7% male  Mean age: 65.1 years  (N=449) | 01/01/2020 - 13/02/2020 | RNA detection of the SARS-CoV-2 in a clinical laboratory |
| Tang, 2020^76^ | China | 53.6% male  Mean age: 54.1 years  (N=183) | 01/01/2020 - 03/02/2020 | RNA detection of the 2019-nCoV in the clinical laboratory |
| Thompson, 2020^77^ | UK | 54.3% male  Mean age: 68.7 years  (N=470) | 12/03/2020 - 19/05/2020 | RT-PCR from a nasopharyngeal swab |
| Torres-Macho, 2020^78^ | Spain | 56.4% male  Median age: 67 years  (N=1968) | 02/03/2020 - 31/05/2020 | RT-PCR assay |
| Toutkaboni, 2020^79^ | Iran | 65.2% male  Mean age: 55 years  (N=1061) | 20/02/2020 - 02/04/2020 | RT-PCR or those suspected of having COVID-19 based on characteristic clinical symptoms and chest CT findings (only 53.6% had a positive RT-PCR test) |
| Trabulus, 2020^80^ | Turkey | 57.1% male  Mean age: 55 years  (N=336) | 15/03/2020 - 01/05/2020 | Positive RT-PCR test result in cases admitted with symptoms, signs and findings (laboratory / radiological) suggestive of COVID-19, according to the national guidelines |
| Van Halem, 2020^81^ | Belgium | 60% male  Mean age: 74 years  (N=319) | 11/03/2020 - 15/04/2020 | RT-PCR |
| Vassiliou, 2021^82^ | Greece | 81.5% male  Mean age: 63 years  (N=38) | 22/03/2020 - 25/10/2020 | RT-PCR in nasopharyngeal swabs |
| Von Meijenfeldt, 2020^83^ | Sweden | 63.7% male  Mean age: NR  (N=102) | 09/04/2020 - 08/06/2020 | diagnosed with COVID-19 based on reverse-transcriptase polymerase chain reaction (RT-PCR) viral RNA detection of nasopharyngeal or oropharyngeal swabs or clinical presentation |
| Vrillon, 2020^84^ | France | 44.7% male  Median age: 90 years  (N=76) | 14/03/2020 - 09/04/2020 | Polymerase chain reaction testing of a nasopharyngeal sample |
| Wang, 2020^85^ | China | 49% male  Mean age: 71 years  (N=339) | 01/01/2020 - 06/02/2020 | RT-PCR |
| Wendel-Garcia, 2020^86^ | International | 75.1% male  Mean age: 63 years  (N=639) | NR – 22/04/2020 | laboratory confirmed SARS-CoV-2 infection by nucleic acid amplification according to the WHO-issued testing guidelines |
| Xu, 2020^87^ | China | 55% male  Median age: 62 years  (N=187, only 145 included in mortality analysis) | 26/12/2020 - 01/03/2020 | Throat swab samples were collected and tested for SARS-CoV-2 following WHO guidelines for qRT-PCR |
| Yan, 2020^88^ | China | 59.1% male  Mean age: 64 years  (N=193) | 10/01/2020 - 24/02/2020 | Laboratory confirmation of covid-19 by RT-PCR |
| Yang, 2020^89^ | China | 67% male  Mean age: 59.7 (13.3) years  (N=52) | 24/12/2019 - 26/01/2020 | RT-PCR |
| Yang, 2020^90^ | China | 52.6% male  Mean age: 57 years  (N=1476) | late December/2019 – 25/02/2020 | The diagnosis of COVID-19 was according to WHO interim guidance and confirmed by RNA detection of SARS-CoV-2 |
| Yao, 2020^91^ | China | 39.8% male  Median age: 52 years  (N=95) | 30/01/2020 - 03/03/2020 | Throat‑swab or sputum specimens were collected from all patients before admission for SARS‑CoV‑2 detection using RT-PCR assays |
| Zhao, 2020^92^ | China | 46.1% male in survivor group, 48.3% male in non-survivor group  Mean age: 48.2 years in survivor groups, 64.7 years in non-survivor group  (N=532) | 07/01/2020 - 28/02/2020 | Upper respiratory tract specimens were positive for SARS-CoV-2 nucleic acid by real-time RT-PCR |
| Zhang, 2020^93^ | China | 57.9% male  Median age: 73 years  (N=19) | 16/01/2020 - 20/02/2020 | Chest CT or qRT-PCR assay |
| Zhou, 2020^94^ | China | 62% male  Mean age: 56.0 years  (N=191) | 29/12/2019 - 31/01/2020 | Detection in respiratory specimens by next-generation sequencing or real-time RT-PCR methods |

**Table S14.** Critical appraisal of included studies (n=94) using Joanna Briggs Institute tool for case series.

| Author | Were there clear criteria for inclusion in the case series? | Was the condition measured in a standard, reliable way for all participa- nts included in the case series?* | Were valid methods used for identificati-on of the condition for all participants included in the case series?* | Did the case series have consecutive inclusion of participants? | Did the case series have complete inclusion of participants? | Was there clear reporting of the demograp-hics of the participants in the study? | Was there clear reporting of clinical information of the participants? | Were the outcomes or follow up results of cases clearly reported? | Was there clear reporting of the presenting site(s)/ clinic(s) demograp-hic information? | Was statistical analysis appropriate? |
| --- | --- | --- | --- | --- | --- | --- | --- | --- | --- | --- |
| Chen^24^ | Yes | Yes | Yes | Yes | Yes | Yes | Yes | Yes | Yes | Yes |
| Deng^30^ | No | Yes | Yes | Unclear | Unclear | No | Yes | Yes | Yes | Yes |
| Guo^38^ | Yes | Yes | Yes | Yes | Yes | No | Yes | Yes | Yes | Yes |
| Tang^75^ | Yes | Yes | Yes | Yes | Yes | Yes | Yes | Yes | Yes | Yes |
| Tang^76^ | No | Yes | Unclear | Unclear | Unclear | Yes | No | Yes | Yes | No |
| Yang^89^ | Yes | Yes | Yes | No | Unclear | Yes | Yes | Yes | No | Yes |
| Zhou^94^ | Yes | Yes | Yes | Yes | Yes | Yes | Yes | Yes | No | Yes |
| Zhao^92^ | Yes | Yes | Yes | Unclear | Unclear | Yes | Yes | Yes | No | Yes |
| Sovik^71^ | Yes | Yes | Yes | Yes | Yes | Yes | Yes | Yes | Yes | Yes |
| Schwarz^67^ | Yes | Yes | Yes | Unclear | No | Yes | Yes | Yes | Yes | Yes |
| Huang^41^ | Yes | Yes | Yes | Unclear | Unclear | Yes | Yes | Yes | Yes | Yes |
| Sun^66^ | Yes | Yes | Yes | Unclear | Unclear | Yes | Yes | Yes | Yes | Yes |
| Sulejmani^72^ | Yes | Yes | Yes | Unclear | Unclear | Yes | Yes | Yes | Yes | Yes |
| Mikami^53^ | Yes | Yes | Yes | Unclear | Unclear | Yes | Yes | Yes | Yes | Yes |
| Sensusaiti^68^ | Yes | Yes | Yes | No | Yes | Yes | Yes | Yes | Yes | Yes |
| Rodriguez-Gonzalez^64^ | Yes | Yes | Yes | Yes | No | Yes | Yes | Yes | Yes | Yes |
| Balfanz^14^ | Yes | Yes | Yes | Yes | Yes | Yes | Yes | Yes | Yes | Yes |
| COVID-ICU Group^28^ | Yes | Yes | Yes | Yes | No | Yes | Yes | Yes | Yes | Yes |
| Xu^87^ | No | Yes | Unclear | Unclear | Yes | Yes | Yes | Yes | Yes | Yes |
| Sweeney^73^ | Yes | Yes | Yes | No | Yes | Yes | Yes | Yes | No | Yes |
| Yao^91^ | Yes | Yes | Yes | Yes | No | Yes | Yes | Yes | Yes | Yes |
| Zhang^93^ | Yes | Yes | Yes | Unclear | Unclear | Yes | Yes | Yes | Yes | Yes |
| Gayam^36^ | Yes | Yes | Yes | Yes | Yes | Yes | Yes | Yes | Yes | Yes |
| Kim^44^ | Yes | Yes | Yes | No | No | Yes | Yes | Yes | Yes | Yes |
| Cuvelier^29^ | Yes | Yes | Yes | Yes | Yes | Yes | Yes | Yes | Yes | Yes |
| Li^48^ | Unclear | Yes | Unclear | Unclear | Unclear | Yes | Yes | Yes | Unclear | Yes |
| Wang^85^ | Yes | Yes | Yes | Yes | Yes | No | Yes | Yes | Yes | Yes |
| Van Halem^81^ | Yes | Yes | Yes | No | No | Yes | Yes | Yes | Yes | Yes |
| Vrillon^84^ | Yes | Yes | Yes | Yes | Yes | Yes | Yes | Yes | Yes | Yes |
| Du^33^ | Yes | Yes | Yes | Yes | Yes | Yes | Yes | Yes | No | Yes |
| Kunal^46^ | Yes | Yes | Yes | Yes | Yes | Yes | Yes | Yes | No | Yes |
| Bertsimas^19^ | No | Yes | Yes | No | Yes | Yes | Yes | Yes | Yes | Yes |
| Namendys-Silva^54^ | Yes | Yes | Yes | Unclear | No | Yes | Yes | Yes | Yes | Yes |
| Thompson^77^ | Yes | Unclear | Unclear | No | No | Yes | Yes | Yes | Yes | Yes |
| Rokni^65^ | Yes | Yes | Yes | Yes | Yes | Yes | Yes | Yes | Yes | Yes |
| Yan^88^ | Yes | Yes | Yes | Unclear | Unclear | Yes | Yes | Yes | Yes | Yes |
| Bonetti^20^ | Yes | Yes | Yes | Unclear | No | Yes | Yes | Yes | Yes | Yes |
| Kayina^42^ | Yes | Yes | Yes | Yes | Yes | Yes | Yes | Yes | Yes | Yes |
| Chen^25^ | Yes | Yes | Yes | Yes | No | Yes | Yes | Yes | Yes | Yes |
| Li^49^ | Yes | Yes | Yes | Yes | No | Yes | Yes | Yes | Yes | Yes |
| Serin^69^ | Yes | Unclear | Unclear | Unclear | Yes | Yes | Yes | Yes | No | Yes |
| Gadotti^34^ | Yes | Yes | Yes | No | No | Yes | Yes | Yes | No | Yes |
| Berenguer^18^ | No | Yes | Yes | Yes | Unclear | Yes | Yes | Yes | Yes | Yes |
| Mandel^50^ | Yes | Unclear | Yes | Unclear | Unclear | Yes | Yes | Yes | No | Yes |
| Parker^60^ | Yes | Yes | Yes | Yes | Yes | Yes | Yes | Yes | Yes | Yes |
| Neumann-Podczaska^55^ | Yes | Yes | Yes | Yes | Yes | Yes | Yes | Yes | Yes | Yes |
| Park^59^ | Yes | Yes | Yes | Unclear | Unclear | Yes | Yes | Yes | No | Yes |
| Gupta^39^ | No | Yes | Yes | No | No | Yes | Yes | Yes | No | Yes |
| Mendes^52^ | Yes | Yes | Yes | No | Unclear | Yes | Yes | Yes | Yes | Yes |
| Genet^37^ | Yes | Yes | Yes | Yes | Yes | Yes | Yes | Yes | Unclear | Yes |
| Abohamr^2^ | Yes | Yes | Yes | Yes | No | Yes | Yes | Yes | No | Yes |
| Altschul^7^ | Yes | Yes | Yes | Yes | Yes | Yes | Yes | Yes | No | Yes |
| Bannaga^15^ | Yes | Yes | Yes | Unclear | No | Yes | Unclear | Yes | Unclear | Yes |
| Alamdari^3^ | Yes | Yes | Yes | Unclear | Unclear | Yes | Yes | Yes | Yes | Yes |
| Chang^23^ | Yes | Yes | Yes | Unclear | Unclear | Yes | Yes | Yes | Yes | Yes |
| DeSmet^32^ | Yes | Yes | Yes | Unclear | Unclear | Yes | Yes | Yes | No | Yes |
| Lee^47^ | Yes | Yes | Yes | No | No | Yes | Yes | Yes | No | Yes |
| Gurtoo^40^ | No | Yes | Yes | No | Yes | Yes | Yes | Yes | No | Yes |
| Ponsford^61^ | Yes | Yes | Yes | Unclear | Yes | Yes | No | Yes | Yes | Yes |
| Nicholson^56^ | Yes | Yes | Yes | Yes | No | Yes | Yes | Yes | No | Yes |
| Garcia de Guadiana-Romualdo^35^ | Yes | Yes | Yes | Yes | No | Yes | Yes | Yes | Yes | Yes |
| Vassiliou^82^ | Yes | Yes | Yes | Yes | Yes | Yes | Yes | Yes | No | Yes |
| Tahtasakal^74^ | Yes | Yes | Yes | Unclear | No | Yes | Yes | Yes | No | Yes |
| De Michieli^31^ | Yes | Yes | Yes | Unclear | Unclear | Yes | Yes | Yes | Yes | Yes |
| Belaid^17^ | Yes | Yes | Yes | Unclear | Unclear | Yes | Yes | Yes | Yes | Yes |
| Cortes-Telles^27^ | Yes | Yes | Yes | Yes | Yes | Yes | Yes | Yes | Yes | Yes |
| Marcolino^51^ | Yes | Yes | Yes | Yes | No | Yes | Yes | Yes | Yes | Yes |
| Oliynyk^58^ | Yes | Yes | Yes | No | No | Yes | Yes | Yes | No | Yes |
| von Meijenfeldt^83^ | Yes | Unclear | Unclear | Unclear | No | Yes | Yes | Yes | Yes | Yes |
| Omar^57^ | Yes | Yes | Yes | Unclear | Unclear | Yes | Yes | Yes | Yes | Yes |
| Rath^62^ | Yes | Yes | Yes | Yes | Yes | Yes | Yes | Yes | No | Yes |
| Arifputra^8^ | Yes | Yes | Yes | Unclear | Yes | Yes | Yes | Yes | No | Yes |
| Abdeladim^1^ | Yes | Yes | Yes | Yes | No | Yes | Yes | Yes | Yes | Yes |
| Toutkaboni^79^ | Yes | Yes | No | Yes | Yes | Yes | Yes | Yes | Yes | Yes |
| Ayed^13^ | Yes | Yes | Yes | Unclear | Unclear | Yes | Yes | Yes | Yes | Yes |
| Alharthy^5^ | Yes | Yes | Yes | Yes | No | Yes | Yes | Yes | Yes | Yes |
| Torres-Macho^78^ | Yes | Yes | Yes | Unclear | Yes | Yes | Yes | Yes | Yes | Yes |
| Yang^89^ | Yes | Yes | Yes | Yes | Unclear | Yes | Yes | Yes | Yes | Yes |
| Alharthy^4^ | Yes | Yes | Yes | No | No | Yes | Yes | Yes | Yes | Yes |
| Wendel Garcia^86^ | Yes | Yes | Yes | Unclear | Unclear | Yes | Yes | Yes | Yes | Yes |
| Kokoszka-Bargiel^45^ | Yes | Yes | Yes | Unclear | No | Yes | Yes | Yes | Yes | Yes |
| Trabulus^80^ | Yes | Yes | Yes | Yes | Yes | Yes | Yes | Yes | Yes | Yes |
| Almazeedi^6^ | Yes | Yes | Yes | Yes | Yes | Yes | Yes | Yes | Yes | Yes |
| Brill^22^ | Yes | Yes | Yes | Yes | Yes | Yes | Yes | Yes | Yes | Yes |
| Shi^70^ | Yes | Yes | Yes | No | No | Yes | Yes | Yes | Yes | Yes |
| Barman^16^ | Yes | Yes | Yes | Yes | Yes | Yes | Yes | Yes | No | Yes |
| Ciceri^26^ | Yes | Yes | Yes | Yes | No | Yes | Yes | Yes | Yes | Yes |
| Khoshnood^43^ | Yes | Yes | Yes | Unclear | Yes | Yes | Yes | Yes | Yes | Yes |
| Brandao Neto^21^ | Yes | Yes | Yes | Unclear | Yes |  | Yes | Yes | Yes | Yes |
| Asghar^9^ | Yes | Yes | Yes | Yes | Yes | Yes | Yes | Yes | Yes | Yes |
| Asghar^10^ | Yes | Yes | Yes | Yes | Yes | Yes | Yes | Yes | Yes | Yes |
| Rizo-Tellez^63^ | Yes | Yes | Yes | No | No | Yes | Yes | Yes | Yes | Yes |
| Asghar^11^ | Yes | Unclear | Unclear | Yes | Yes | Yes | Yes | Yes | No | Yes |
| Asghar^12^ | Yes | Yes | Yes | Yes | Yes | Yes | Yes | Yes | Yes | Yes |
| Total "Yes" (%) | 86 (91.5) | 89 (94.7) | 86 (91.5) | 44 (46.8) | 41 (43.6) | 90 (95.8) | 91 (96.8) | 94 (100.0) | 67 (71.3) | 1. 98.9) |

*For JBI questions 2 and 3, “standard, reliable” and “valid” methods were considered to be anti-SARS-CoV-2 IgM and/or IgG antibody assay.

**Table S15.** Critical appraisal of included studies conducted in China (n=21) and studies conducted in other countries (n=73) using Joanna Briggs Institute tool for case series.

| Author | Were there clear criteria for inclusion in the case series? | Was the condition measured in a standard, reliable way for all participa- nts included in the case series? | Were valid methods used for identification of the condition for all participants included in the case series? | Did the case series have consecutive inclusion of participants? | Did the case series have complete inclusion of participants? | Was there clear reporting of the demograp-hics of the participants in the study? | Was there clear reporting of clinical information of the participants? | Were the outcomes or follow up results of cases clearly reported? | Was there clear reporting of the presenting site(s)/ clinic(s) demograp-hic information? | Was statistical analysis appropriate? |
| --- | --- | --- | --- | --- | --- | --- | --- | --- | --- | --- |
| Studies conducted in China (n=21) | 17 (81.6) | 21 (100.0) | 18 (85.7) | 10 (47.6) | 7 (33.3) | 18 (85.7) | 20 (95.2) | 21 (100.0) | 16 (76.2) | 20 (95.2) |
| Studies conducted in other countries (n=73) | 69 (94.5) | 68 (93.2) | 68 (93.2) | 34 (46.6) | 34 (46.6) | 72 (98.6) | 71 (97.3) | 73 (100.0) | 51 (69.9) | 73 (100.0) |
| Chi-square P-value | 0.049 | 0.22 | 0.28 | 0.93 | 0.28 | 0.01 | 0.64 |  | 0.57 | 0.06 |

**Appendix S1: FOREST PLOTS OF INFLAMMATORY BIOMARKERS (18 BIOMARKERS)**


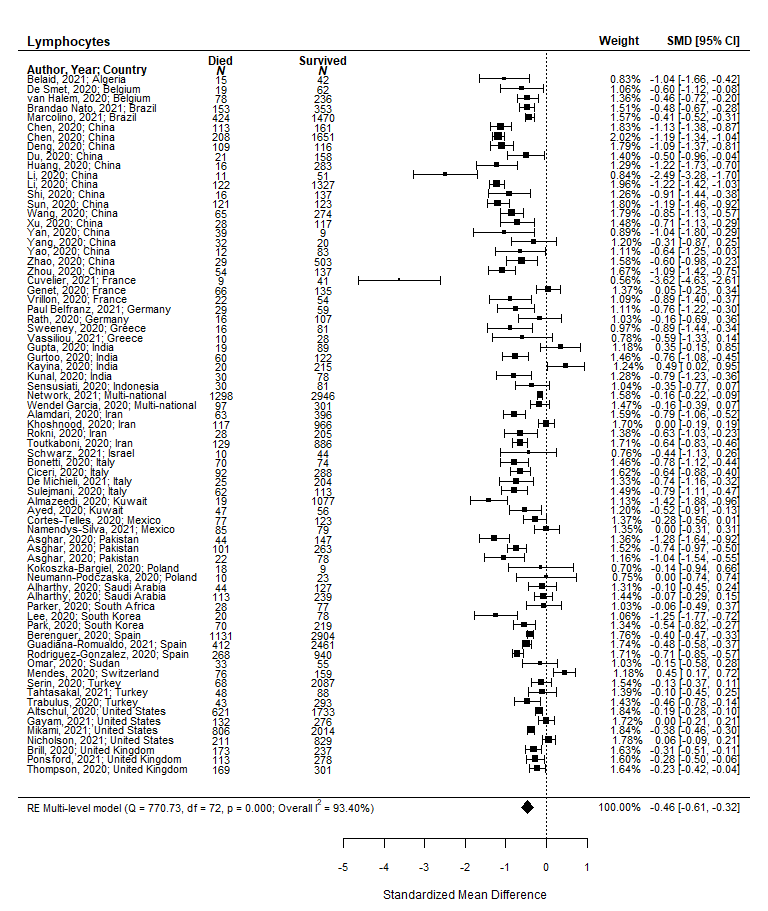
 **Figure S1.** Forest plot for lymphocytes (n=72 studies).

**Lymphocytes – Effect by country:**


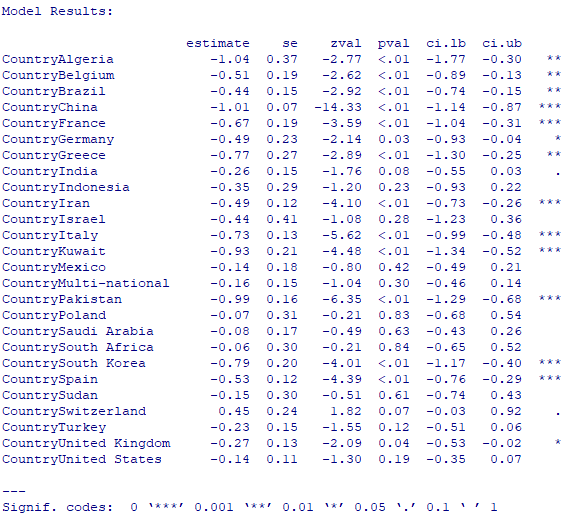


**Estimate** = SMD**; se** = standard error**; ci.lb** = 95% CI lower limit**; ci.ub** = 95% CI upper limit.


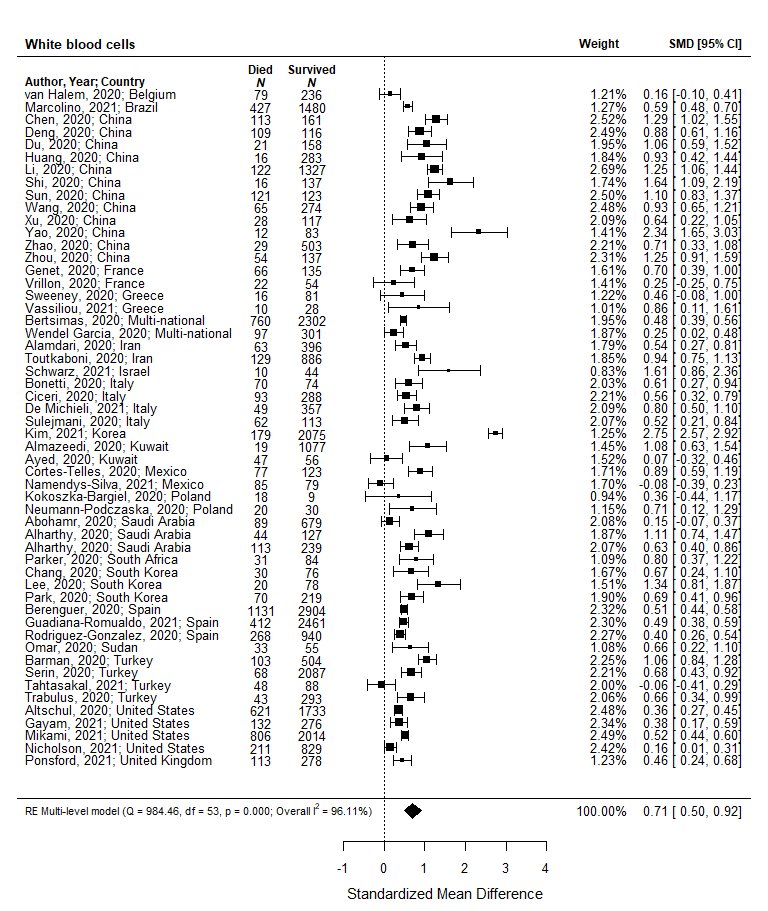
 **Figure S2.** Forest plot for white blood cell count (n=54 studies).

**White blood cells – Effect by country:**


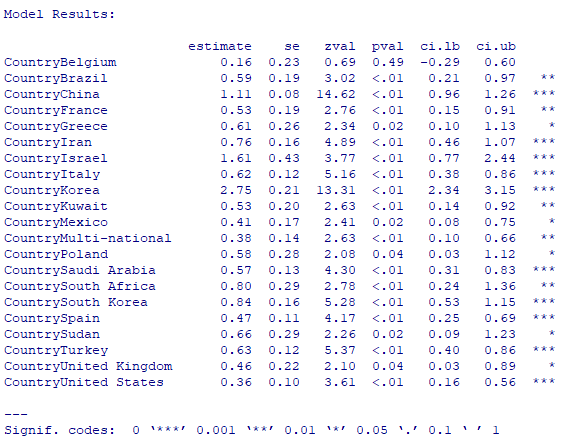


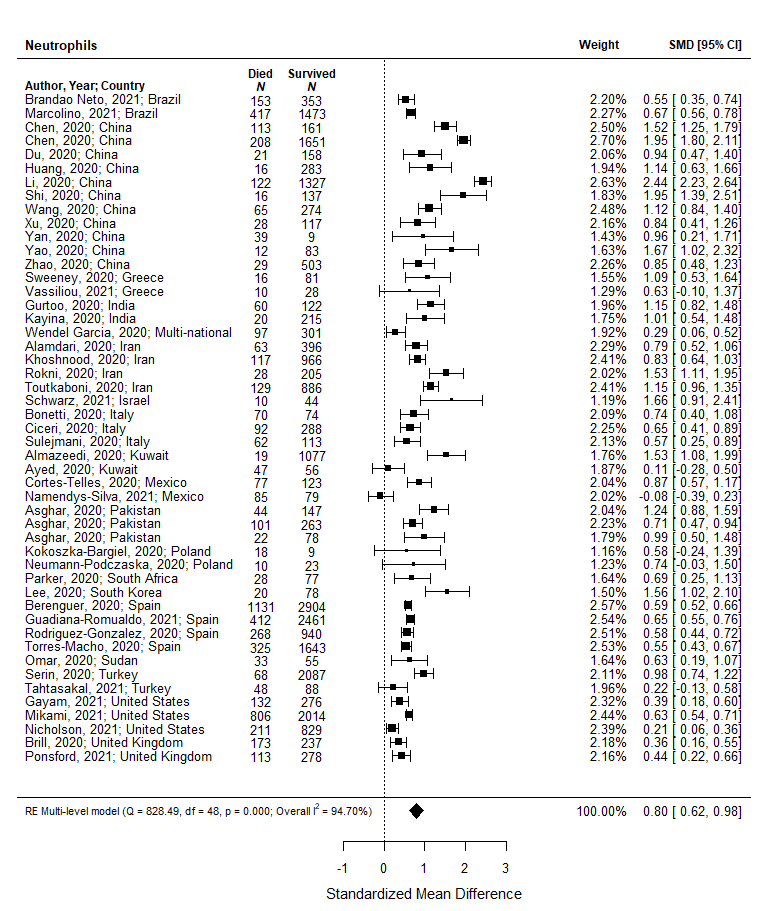


**Figure S3.** Forest plot for neutrophils (n=49 studies).

**Neutrophils – Effect by country:**


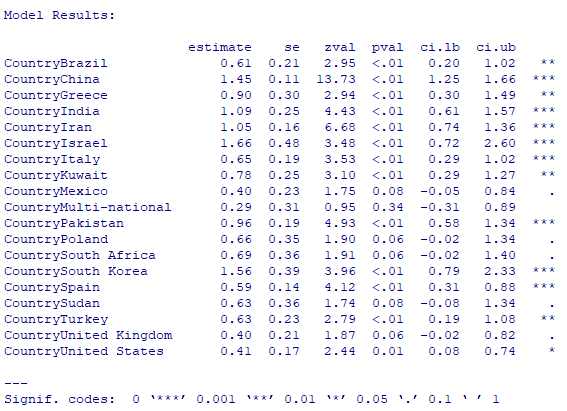


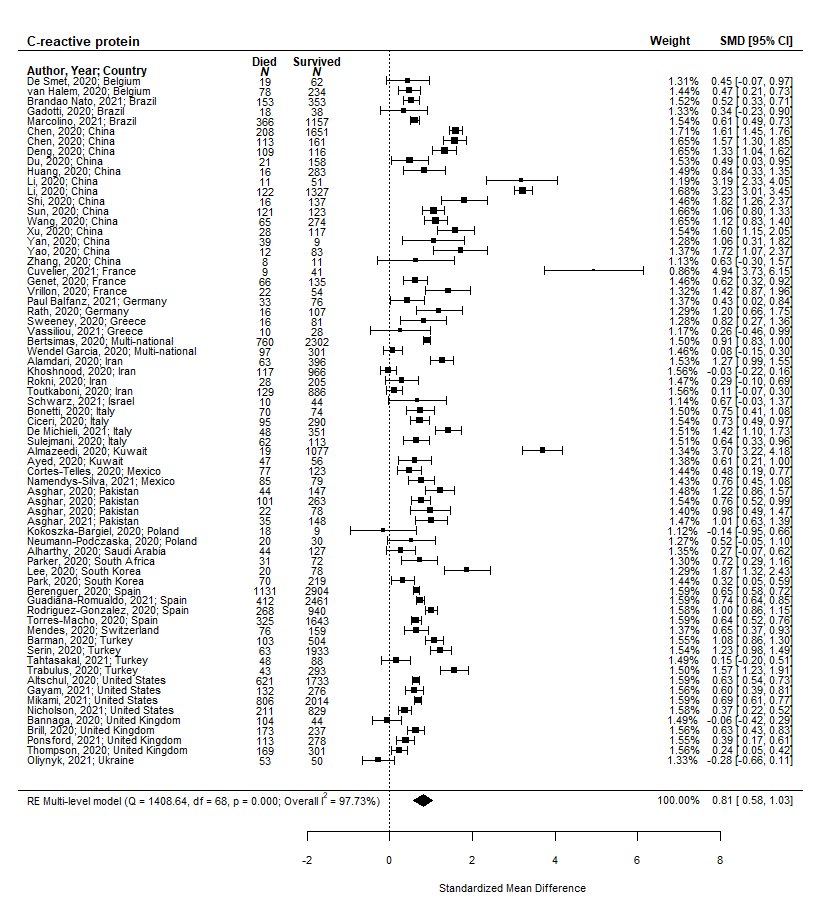


**Figure S4.** Forest plot for C-reactive protein (n=69 studies).

**C-reactive protein – Effect by country:**


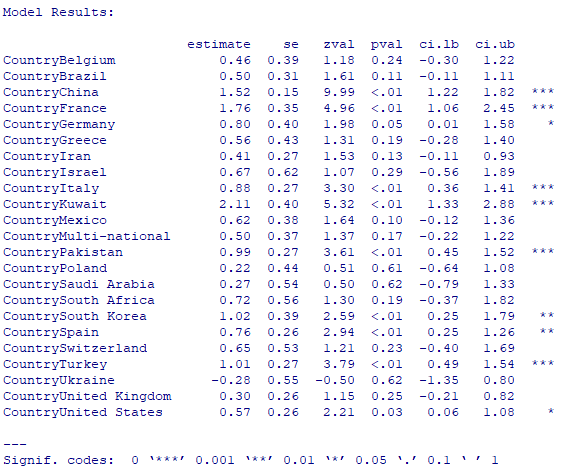


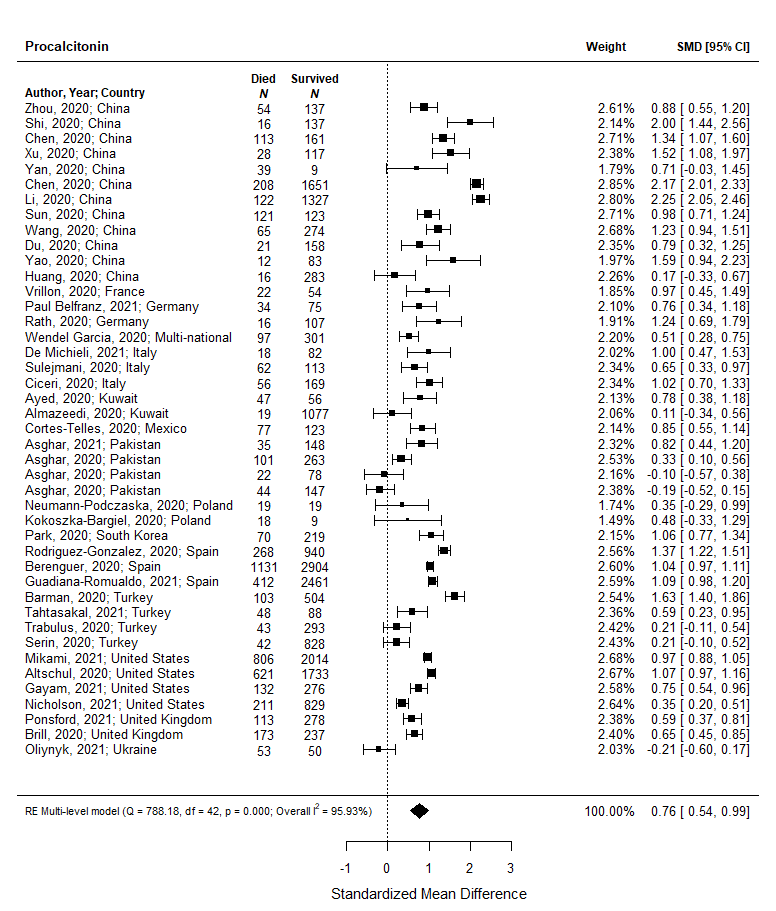


**Figure S5.** Forest plot for procalcitonin (n=43 studies).

**Procalcitonin – Effect by country:**


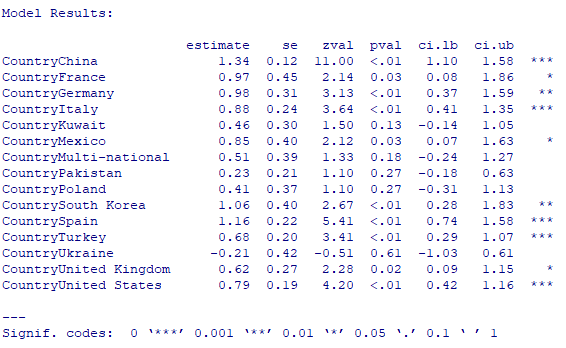


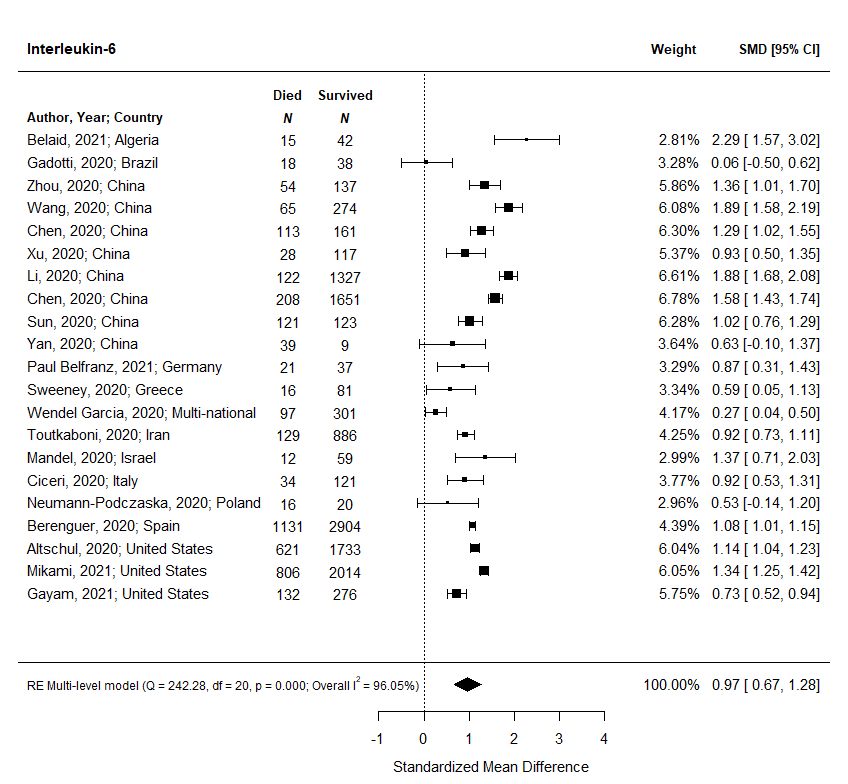
 **Figure S6.** Forest plot for IL-6 (n=21 studies).

**Interleukin-6 – Effect by country:**


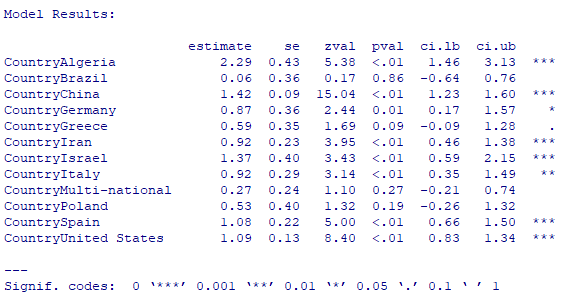


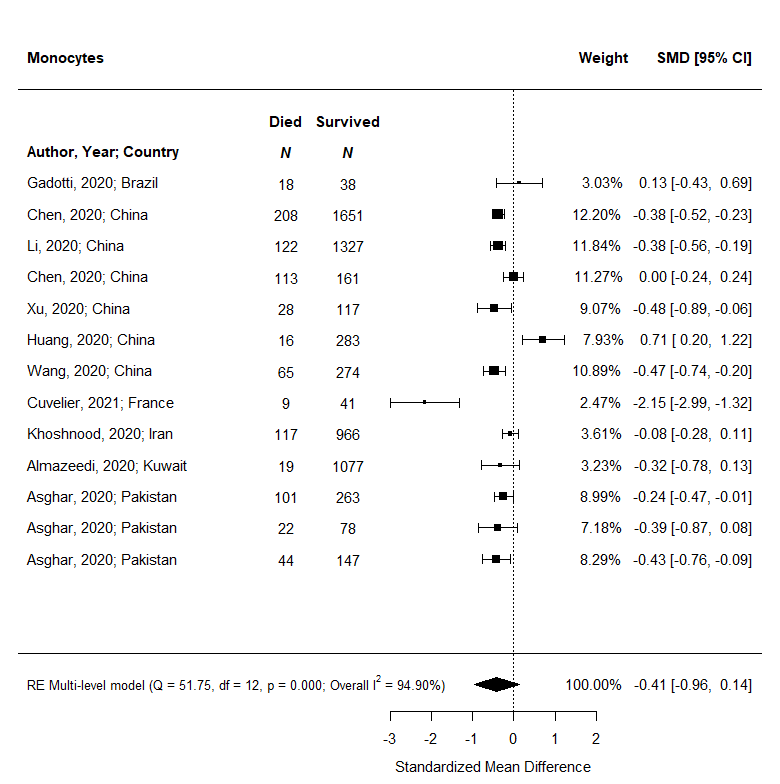


**Figure S7.** Forest plot for monocytes (n=13 studies).

**Monocytes – Effect by country:**


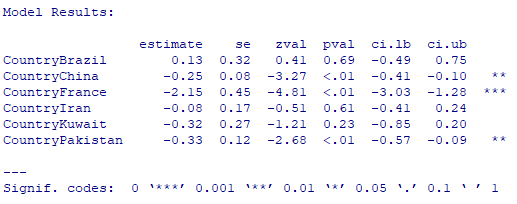


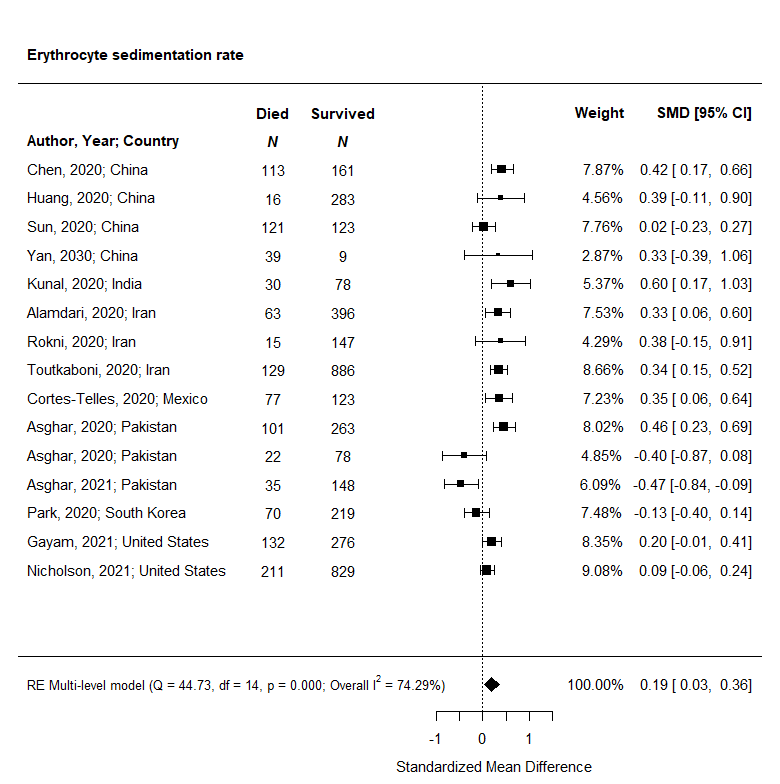


**Figure S8.** Forest plot for erythrocyte sedimentation rate (n=15 studies).

**Erythrocyte sedimentation rate – Effect by country:**


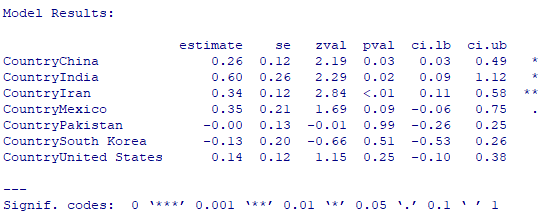


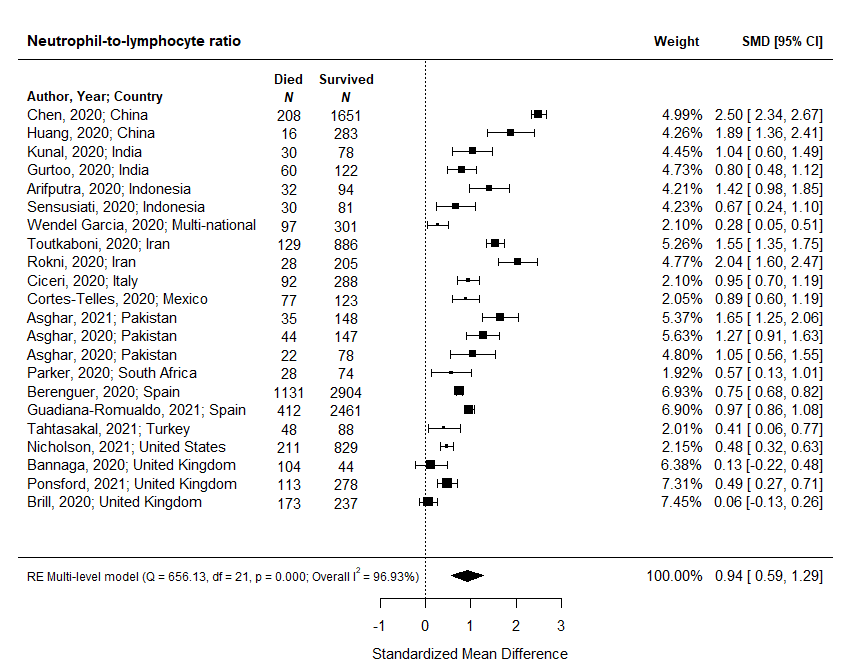


**Figure S9.** Forest plot for neutrophil-to-lymphocyte ratio (n=43 studies).

**Neutrophil-to-lymphocyte ratio – Effect by country:**


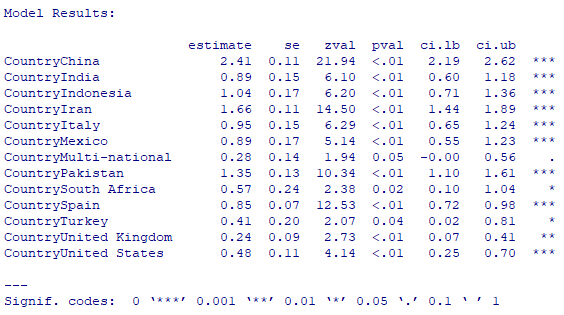


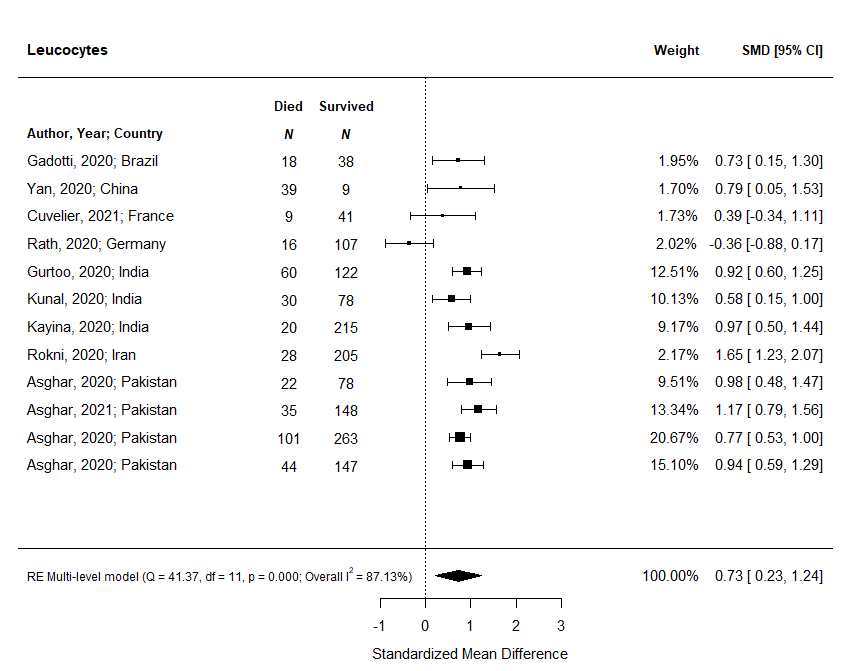


**Figure S10.** Forest plot for leucocytes (n=12 studies).

**Leucocytes – Effect by country:**


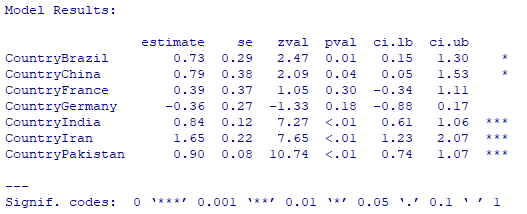


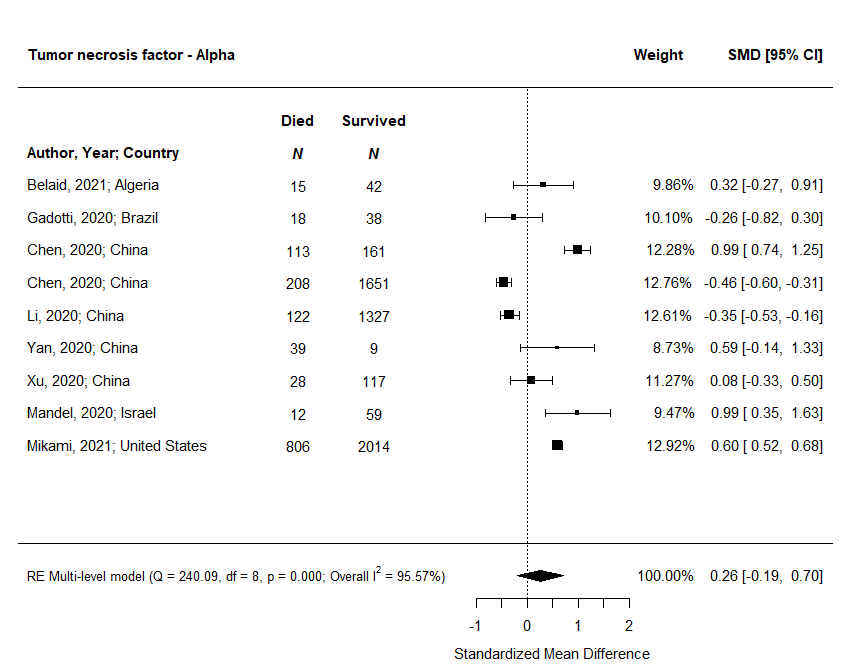


**Figure S11.** Forest plot for Tumor necrosis factor - Alpha (n=9 studies).

**Tumor necrosis factor - Alpha – Effect by country:**


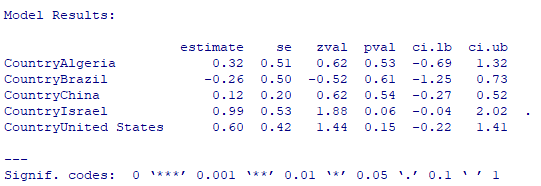


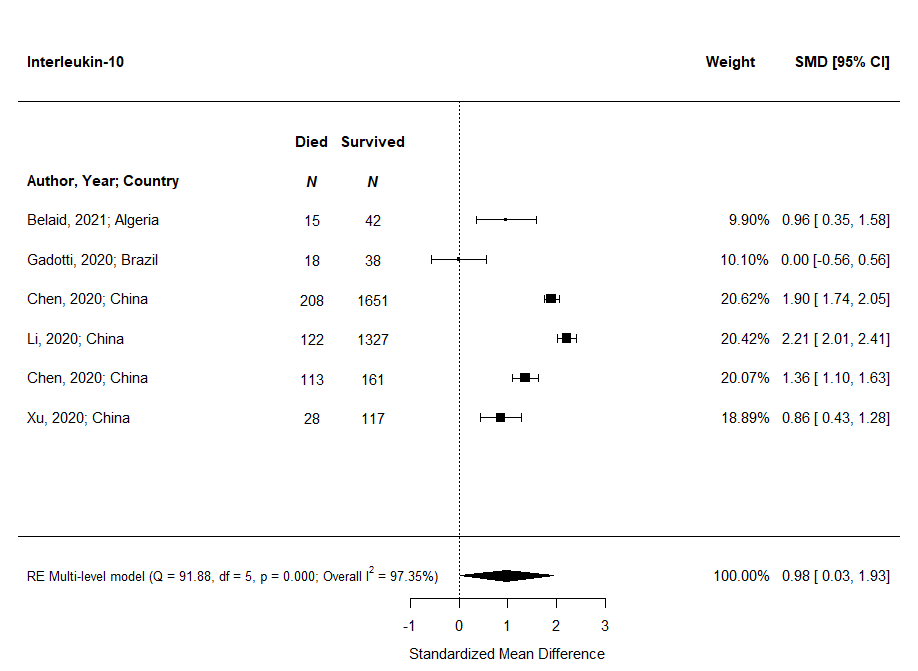


**Figure S12.** Forest plot for IL-10 (n=6 studies).

**Interleukin-10 – Effect by country:**


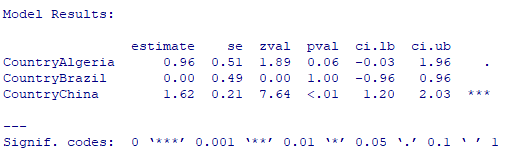


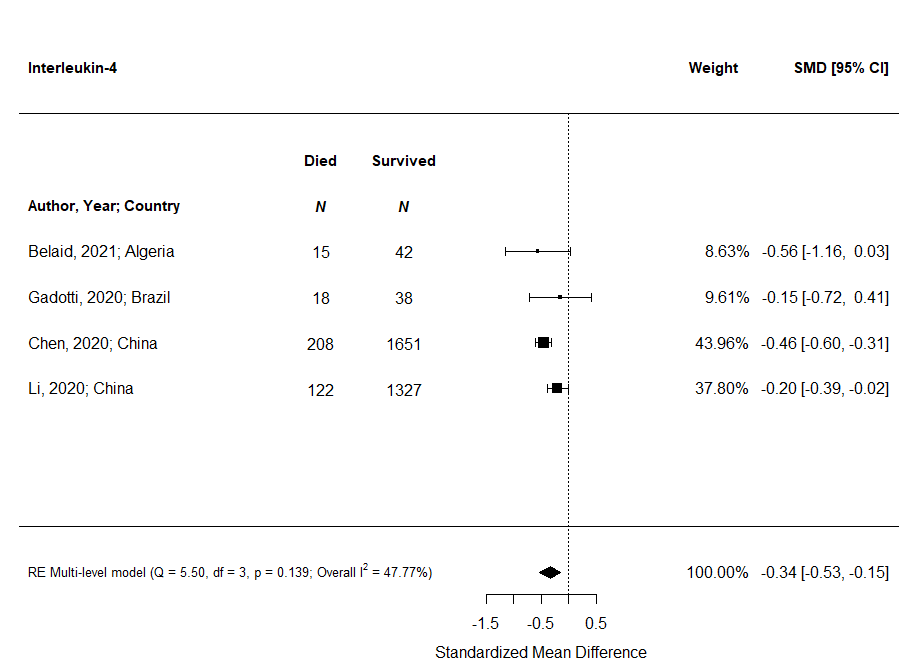


**Figure S13.** Forest plot for IL-4 (n=4 studies).

**Interleukin-4 – Effect by country:**


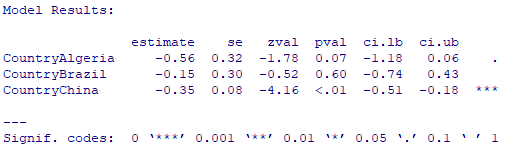


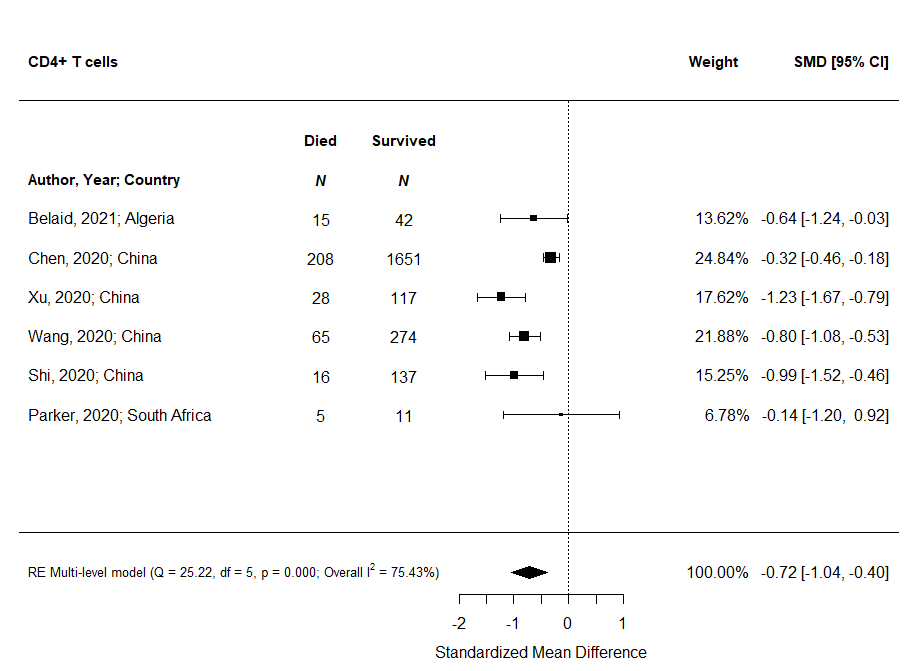


**Figure S14.** Forest plot for CD4+ (n=6 studies).

**CD4+ T cells – Effect by country:**


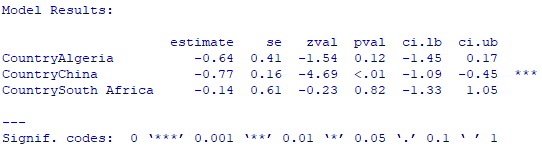


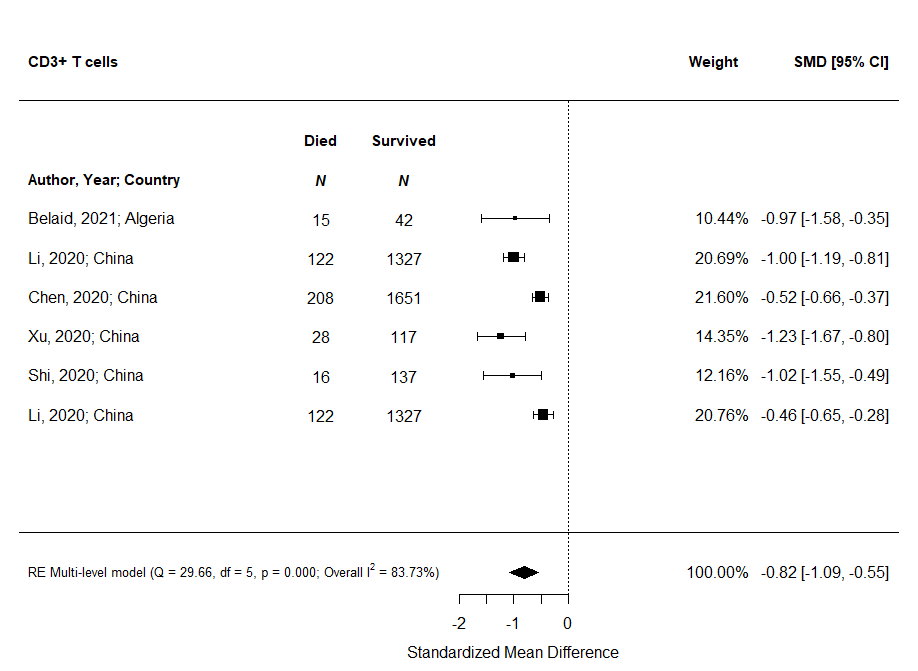
 **Figure S15.** Forest plot for CD3+ (n=6 studies).

**CD3+ T cells – Effect by country:**


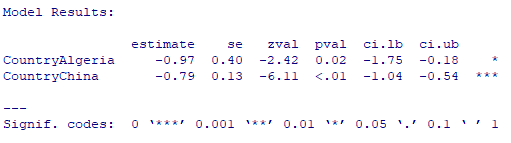


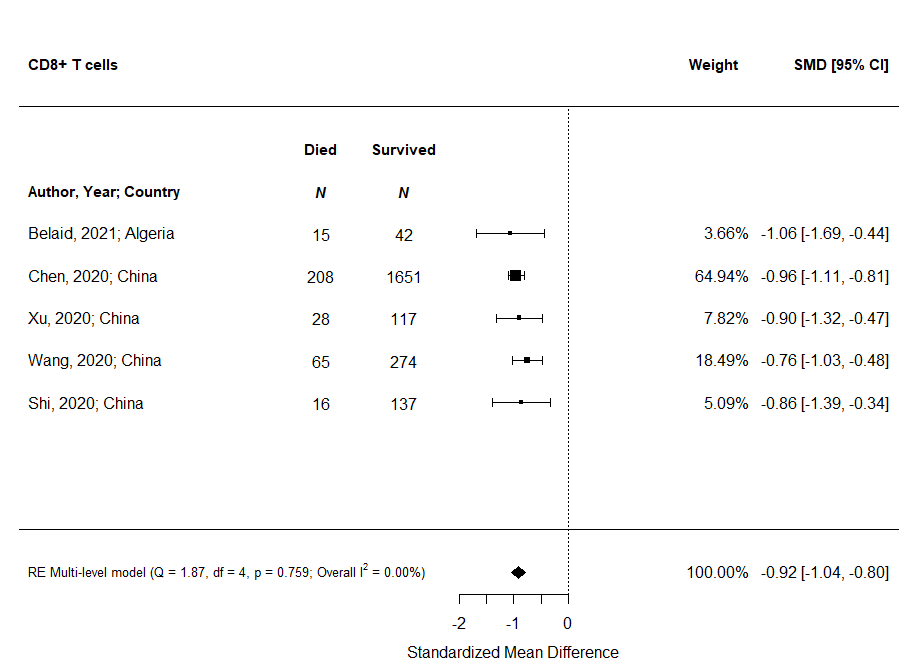


**Figure S16.** Forest plot for CD8+ (n=5 studies).

**CD8+ T cells – Effect by country:**


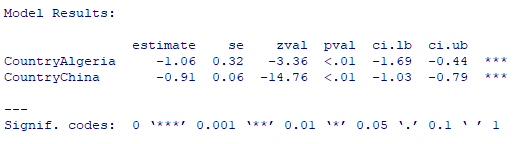


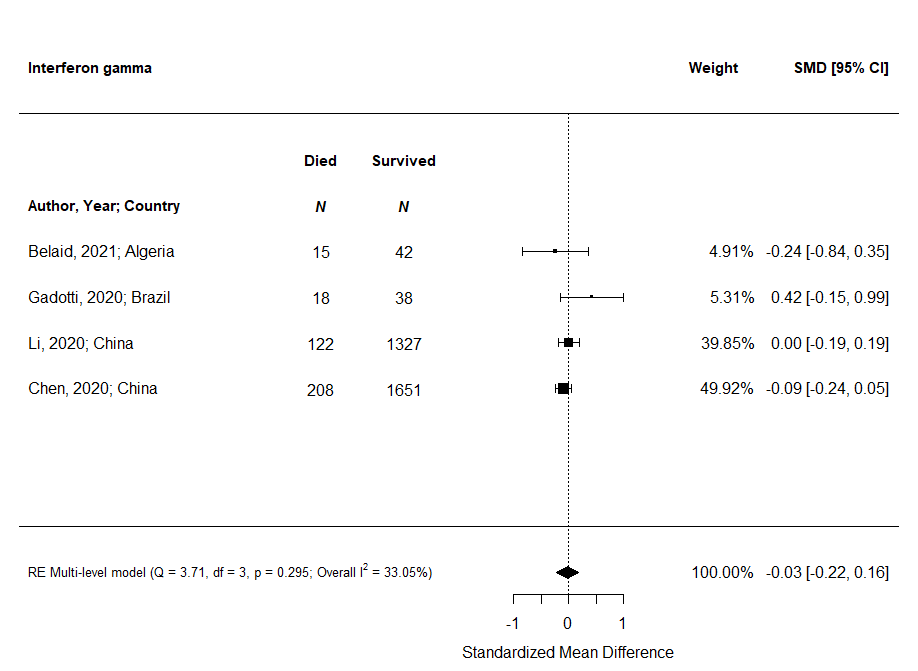


**Figure S17.** Forest plot for IFN-γ (n=4 studies).

**Interferon gamma – Effect by country:**


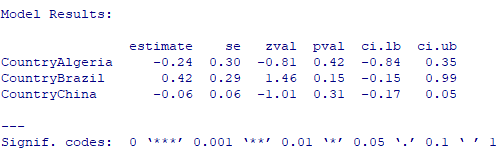


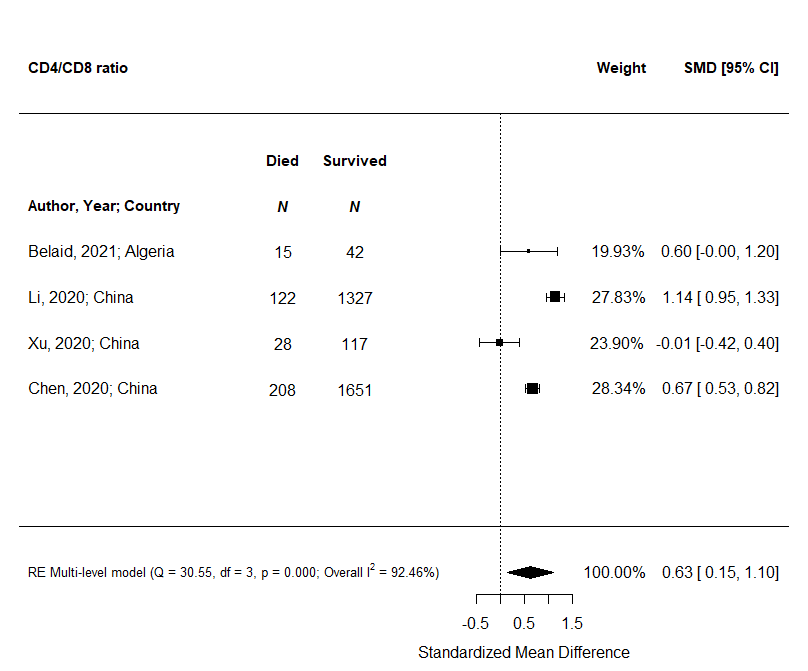


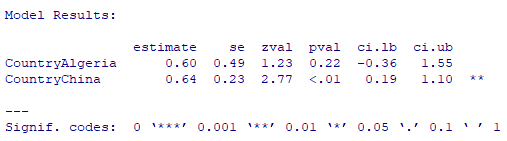


**Figure S18.** Forest plot for CD4/CD8 (n=4 studies).

**FOREST PLOTS OF CARDIAC BIOMARKERS (7 BIOMARKERS)**


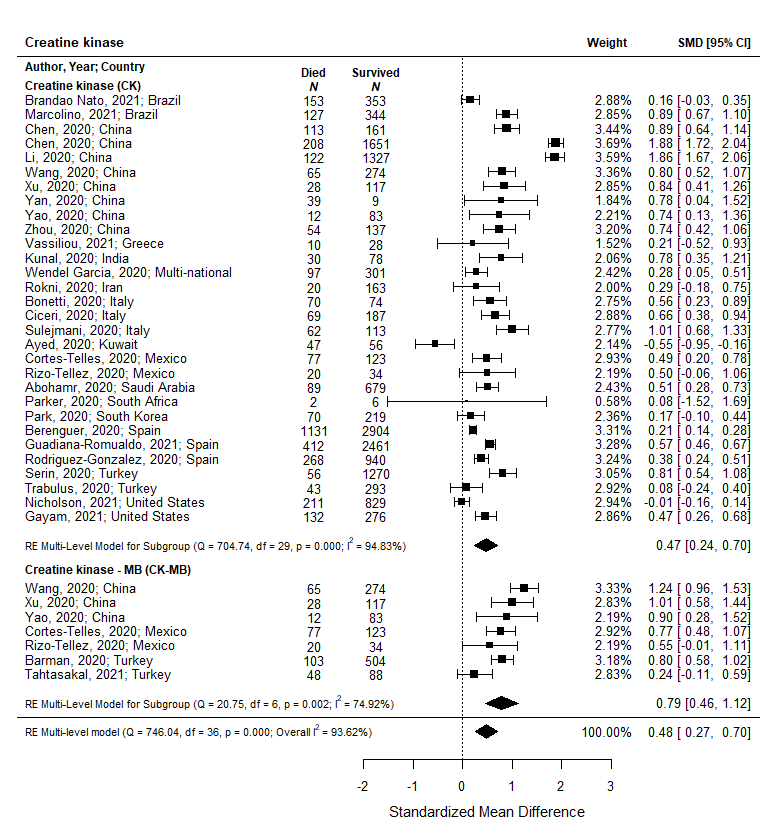


**Figure S19.** Forest plot for CK and CK-MB (n=30 and n=7 studies, respectively).

**Creatine kinase (CK) – Effect by country:**


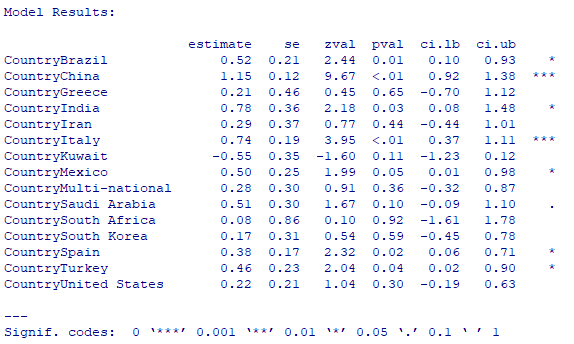


**Creatine kinase - MB (CK-MB) – Effect by country:**


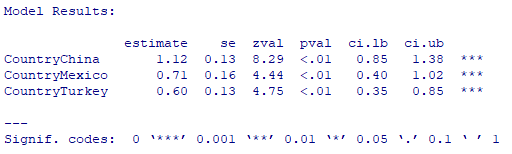


**Estimate** = SMD**; se** = standard error**; ci.lb** = 95% CI lower limit**; ci.ub** = 95% CI upper limit.


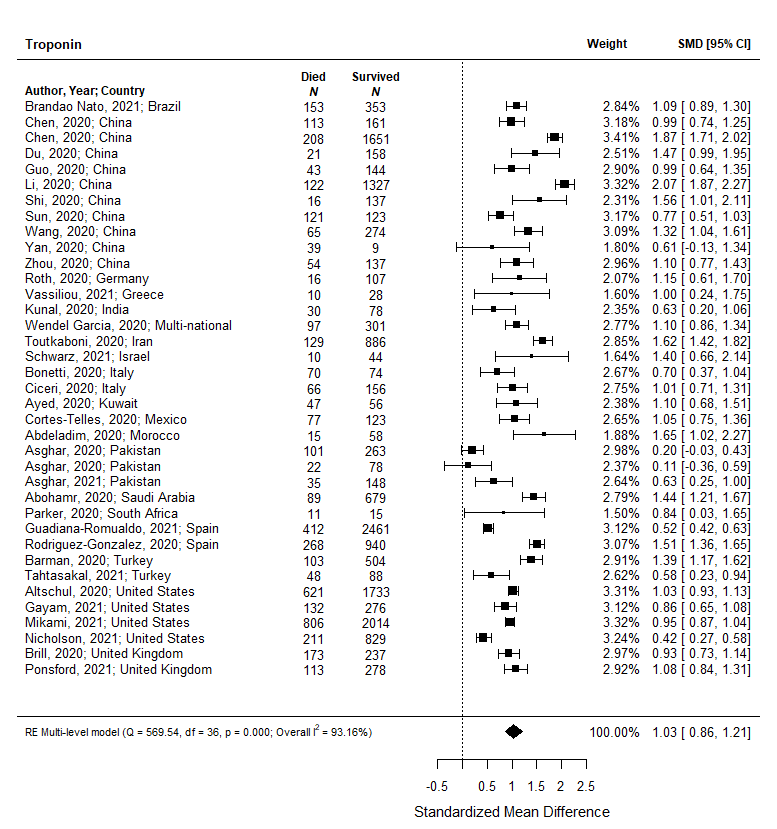


**Figure S20.** Forest plot for troponin (n=37 studies).

**Troponin – Effect by country:**


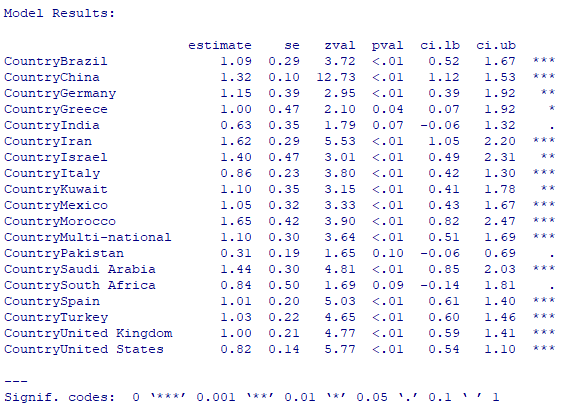


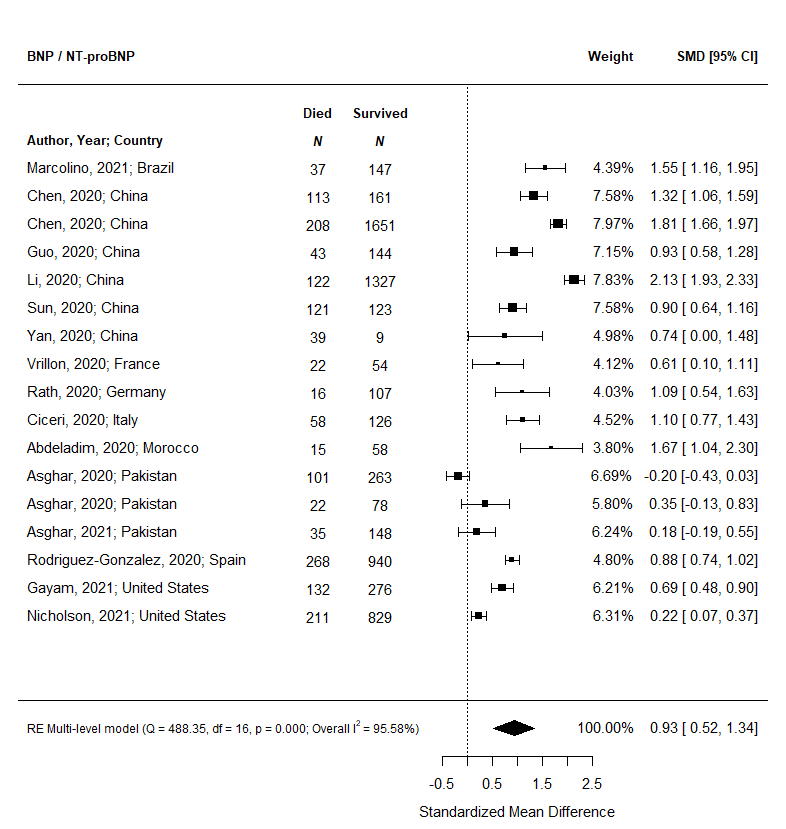


**Figure S21.** Forest plot for BNP/NT pro-BNP (n=17 studies).

**BNP / NT-proBNP – Effect by country:**


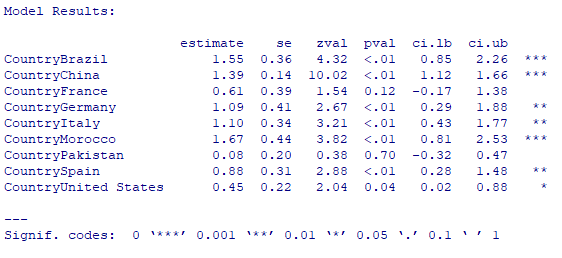


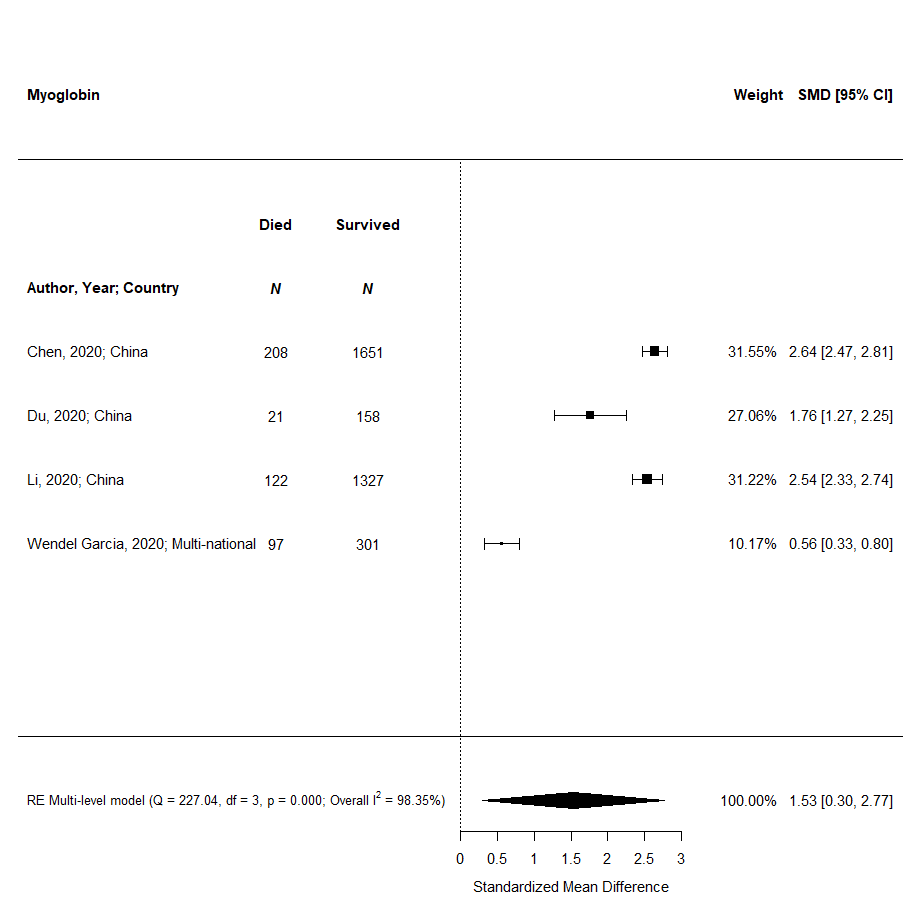


**Figure S22.** Forest plot for myoglobin (n=4 studies).

**Myoglobin – Effect by country:**


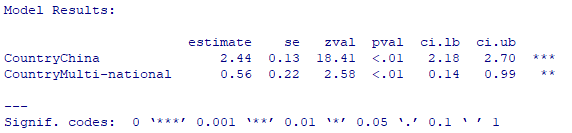


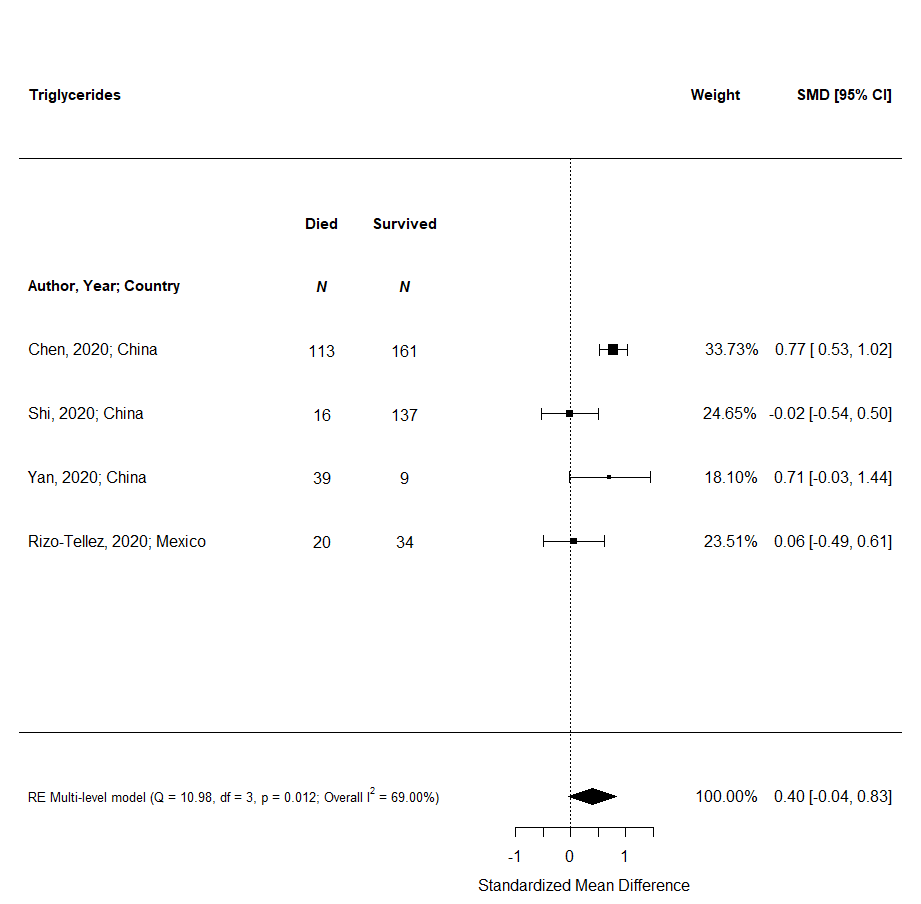


**Figure S23.** Forest plot for triglycerides (n=4 studies).

**Triglycerides – Effect by country:**


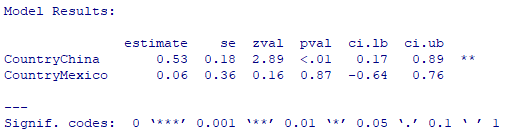


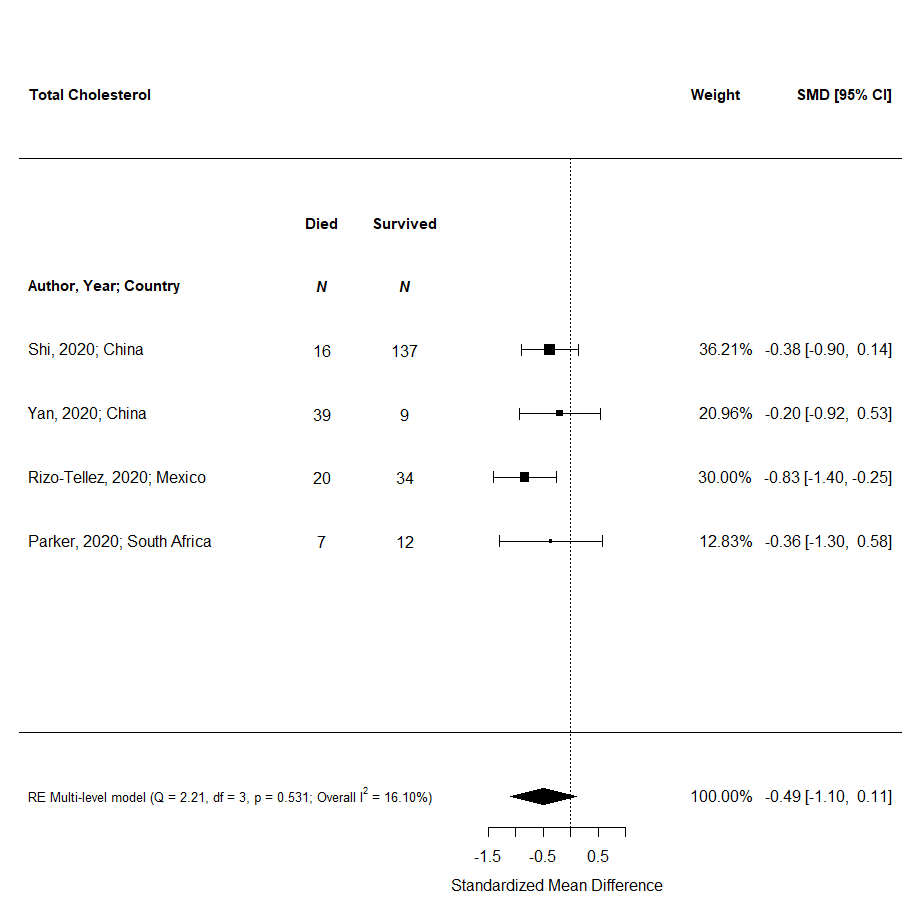


**Figure S24.** Forest plot for cholesterol (n=4 studies).


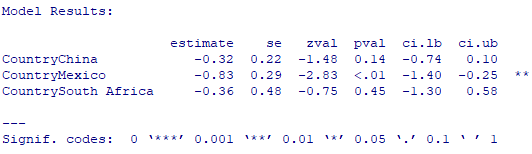


**FOREST PLOTS OF ANEMIA BIOMARKERS (2 BIOMARKERS)**


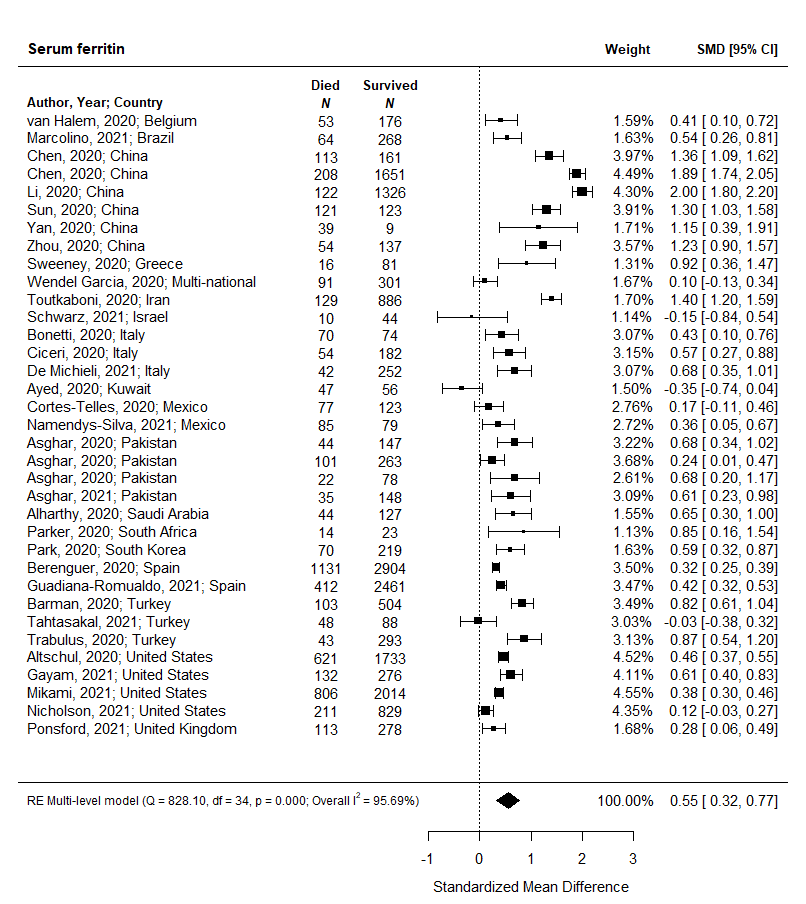


**Figure S25.** Forest plot for ferritin (n=35 studies).

**Serum Ferritin – Effect by country:**


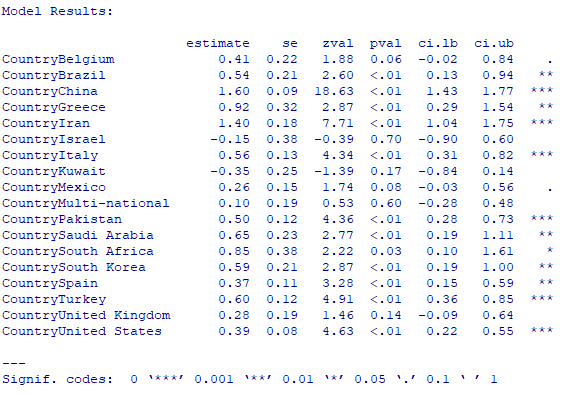


**Estimate** = SMD**; se** = standard error**; ci.lb** = 95% CI lower limit**; ci.ub** = 95% CI upper limit.


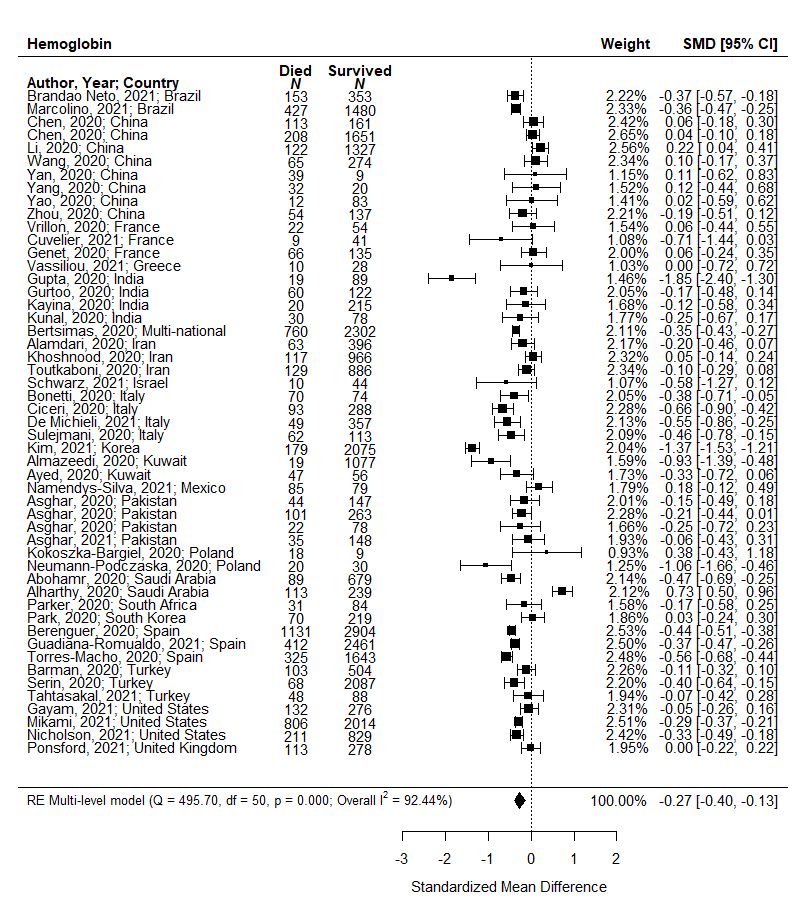


**Figure S26.** Forest plot for hemoglobin (n=51 studies).

**Hemoglobin – Effect by country:**


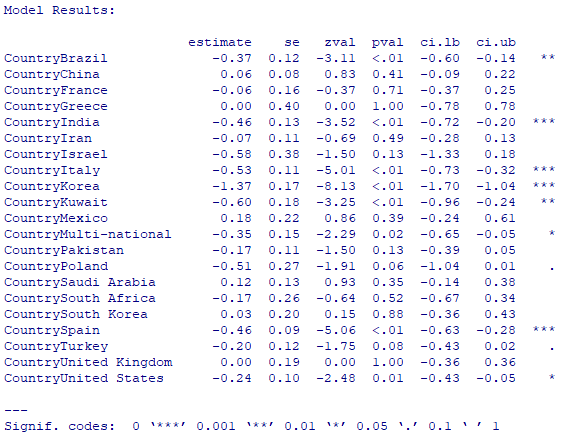


**FOREST PLOTS OF HEPATIC BIOMARKERS (6 BIOMARKERS)**


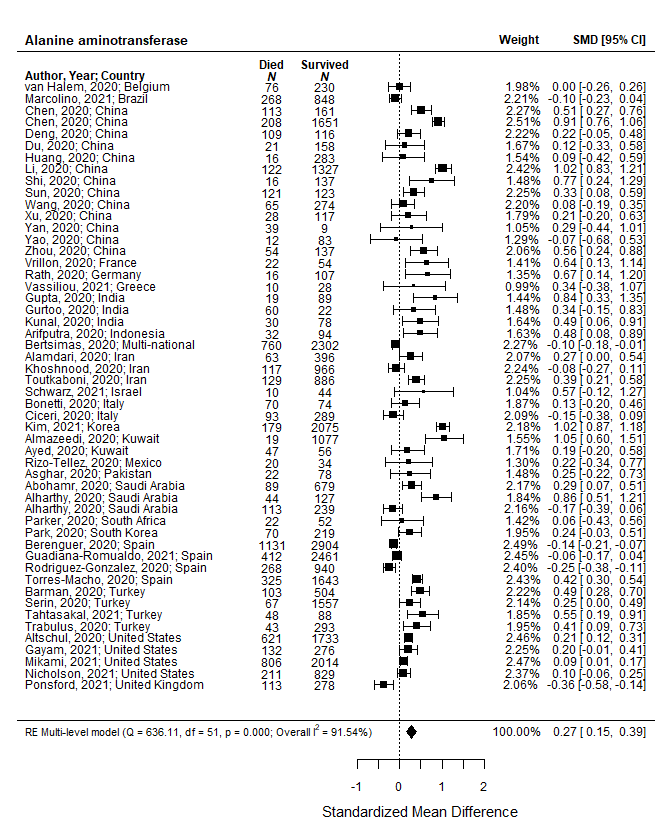


**Figure S27.** Forest plot for alanine aminotransferase (n=52 studies).

**Alanine aminotransferase – Effect by country:**


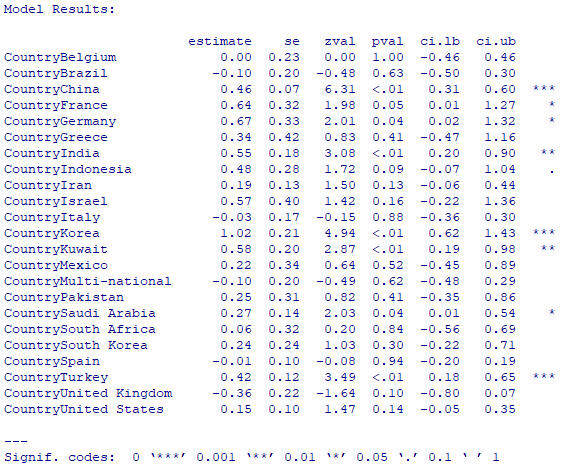


**Estimate** = SMD**; se** = standard error**; ci.lb** = 95% CI lower limit**; ci.ub** = 95% CI upper limit.


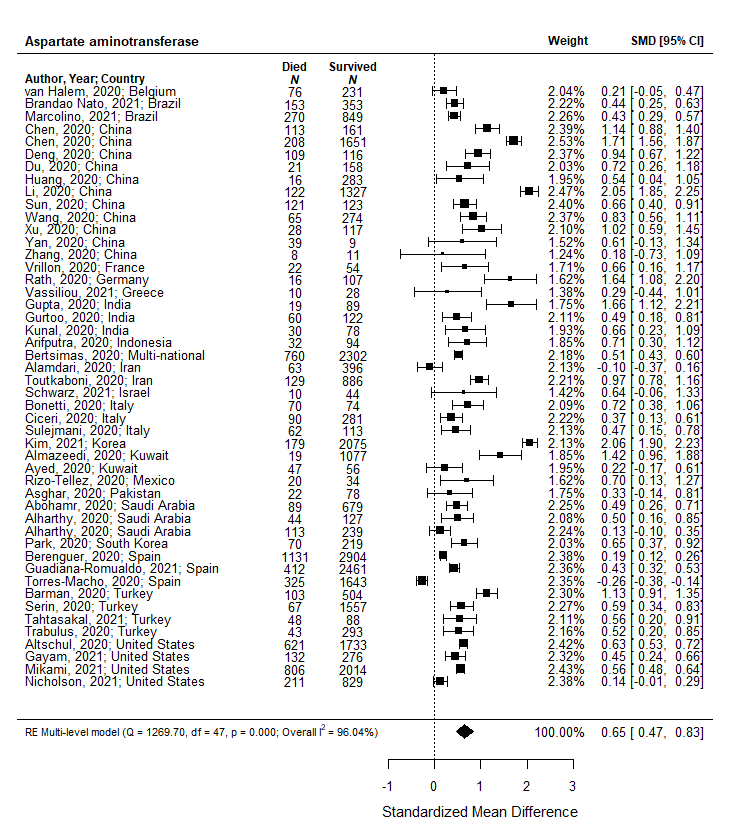


**Figure S28.** Forest plot for aspartate aminotransferase (n=48 studies).

**Aspartate aminotransferase – Effect by country:**


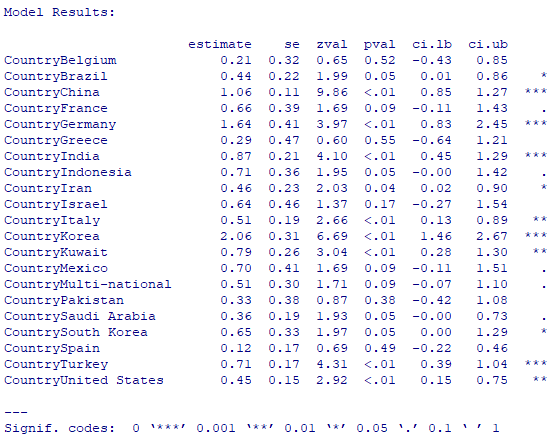


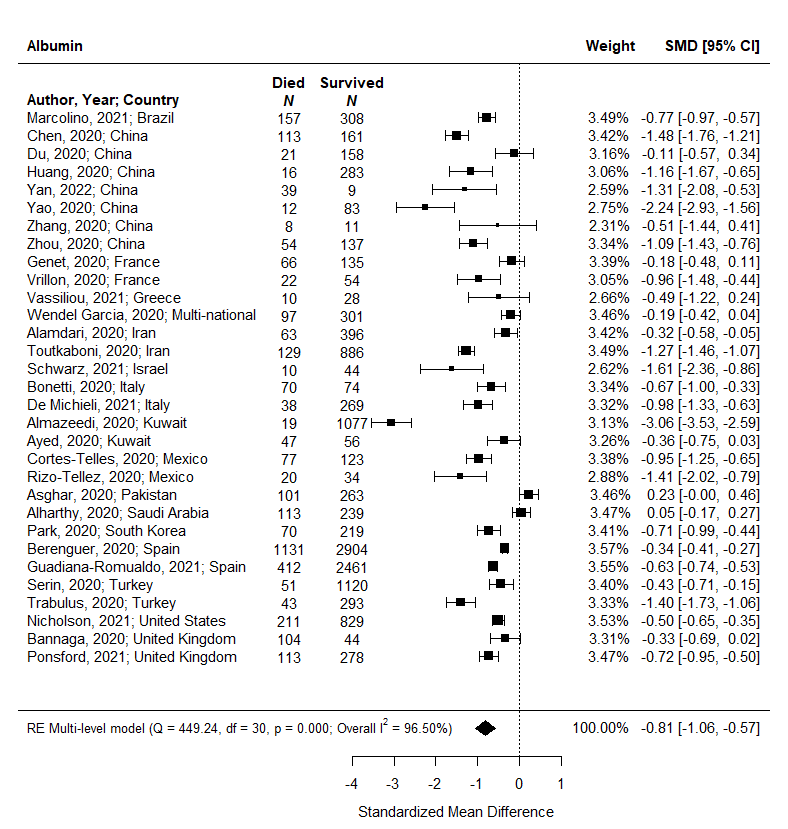


**Figure S29.** Forest plot for albumin (n=31 studies).

**Albumin – Effect by country:**


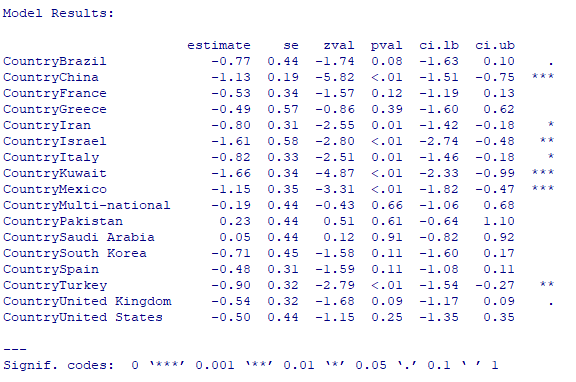


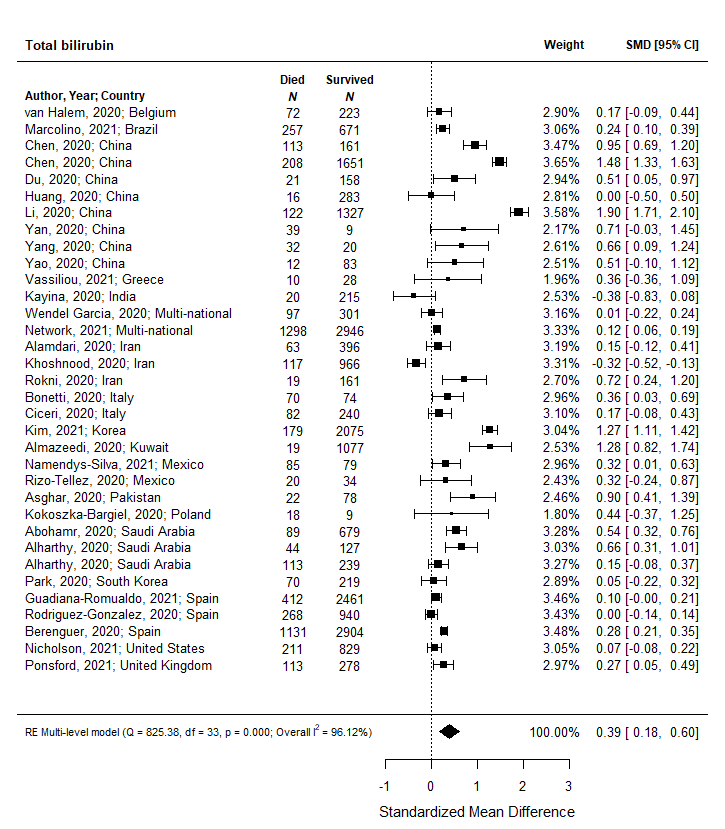


**Figure S30.** Forest plot for bilirubin (n=34 studies).

**Total bilirubin – Effect by country:**


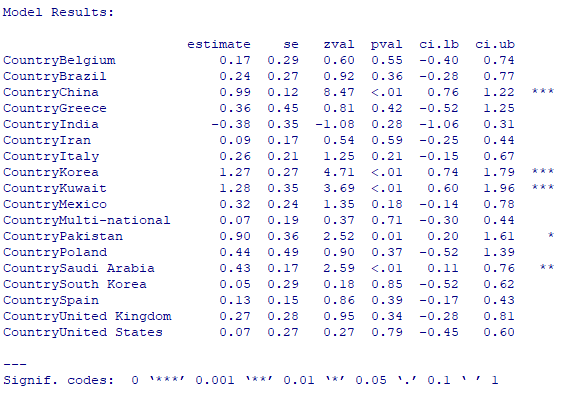


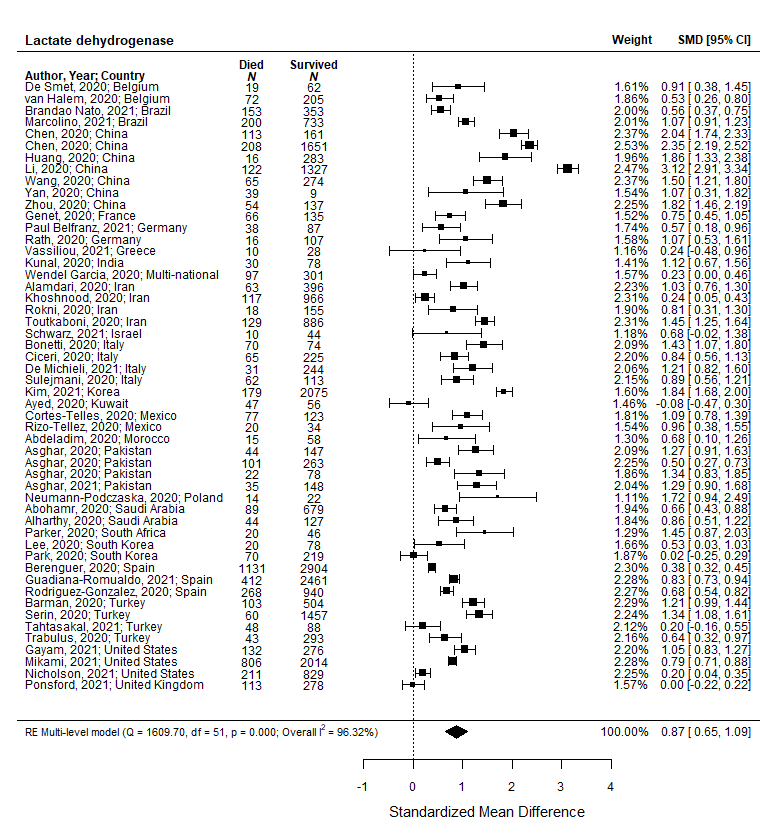


**Figure S31.** Forest plot for lactate dehydrogenase (n=52 studies).

**Lactate dehydrogenase – Effect by country:**


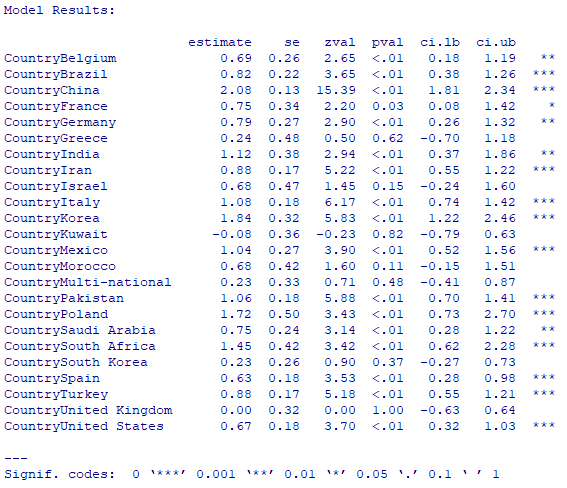


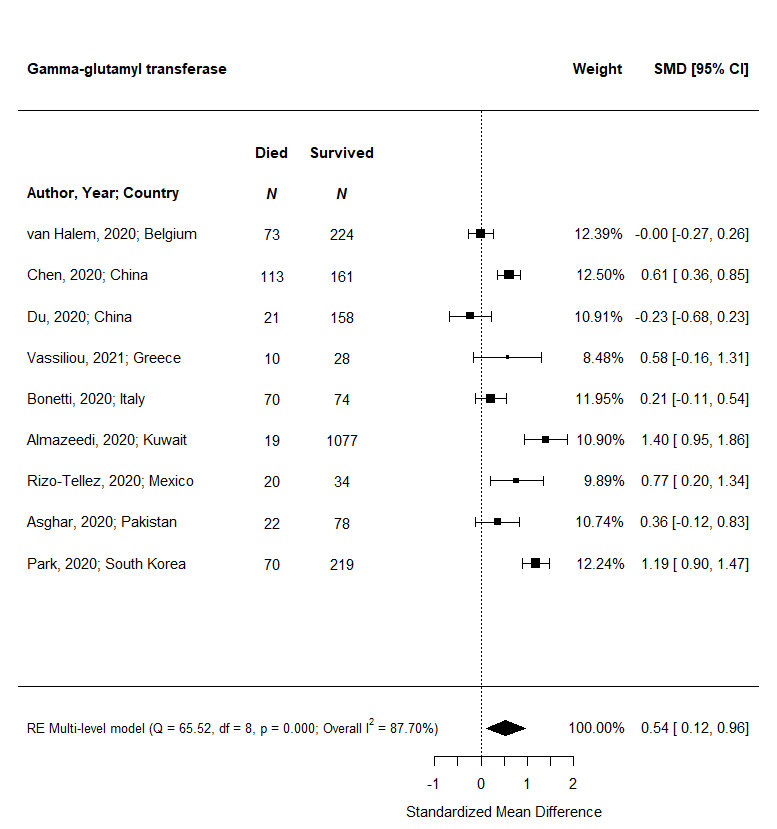


**Figure S32.** Forest plot for gamma-glutamyl transferase (n=9 studies).

**gamma-glutamyl transferase - Effect by country:**


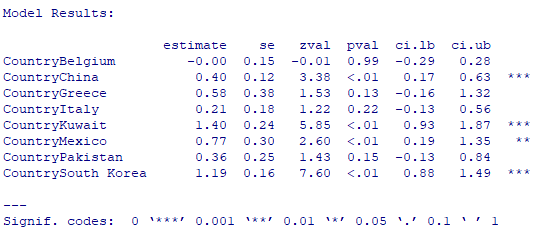


**FOREST PLOTS OF COAGULATION BIOMARKERS (5 BIOMARKERS)**


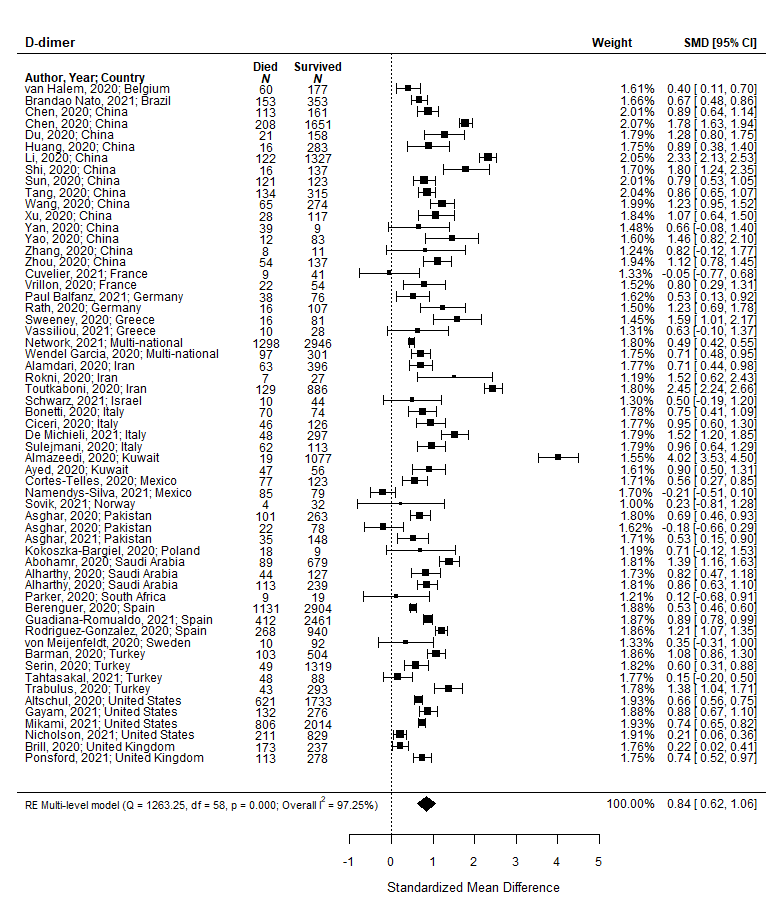


**Figure S33.** Forest plot for D-dimer (n=59 studies).

**D-dimer – Effect by country:**


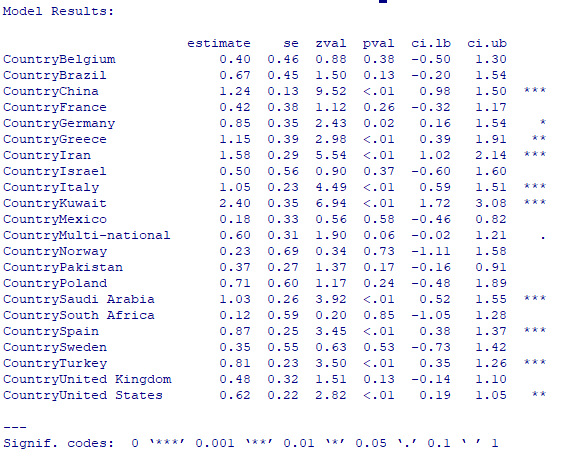


**Estimate** = SMD**; se** = standard error**; ci.lb** = 95% CI lower limit**; ci.ub** = 95% CI upper limit.


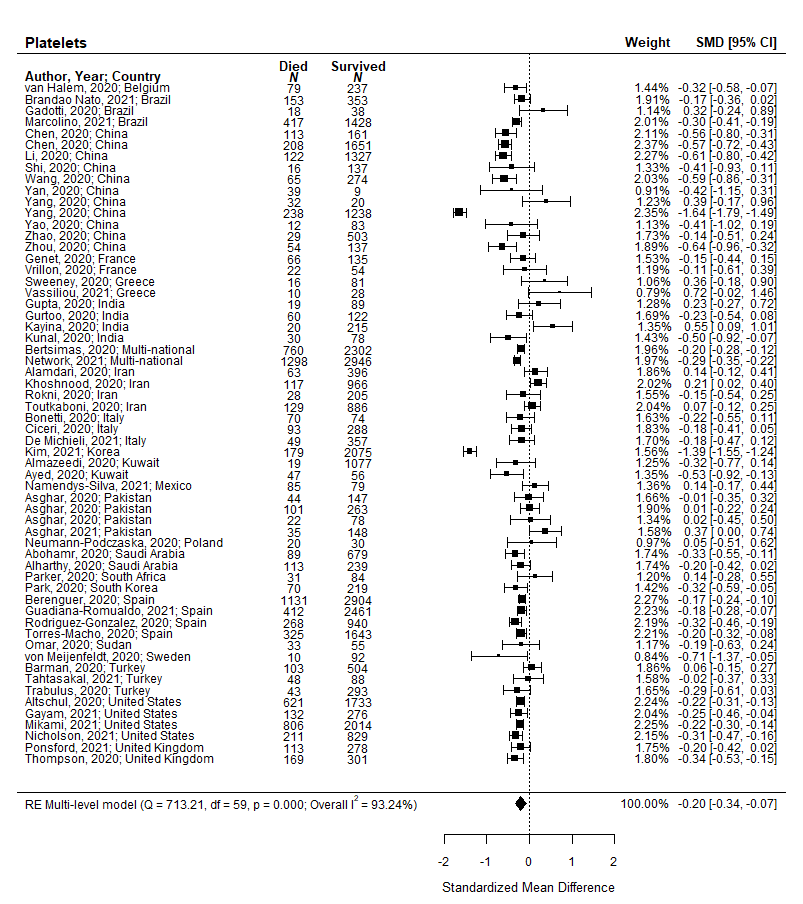


**Figure S34.** Forest plot for platelets (n=60 studies).

**Platelets – Effect by country:**


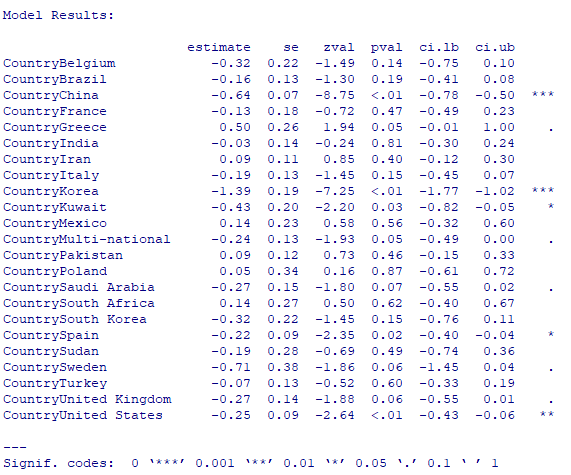


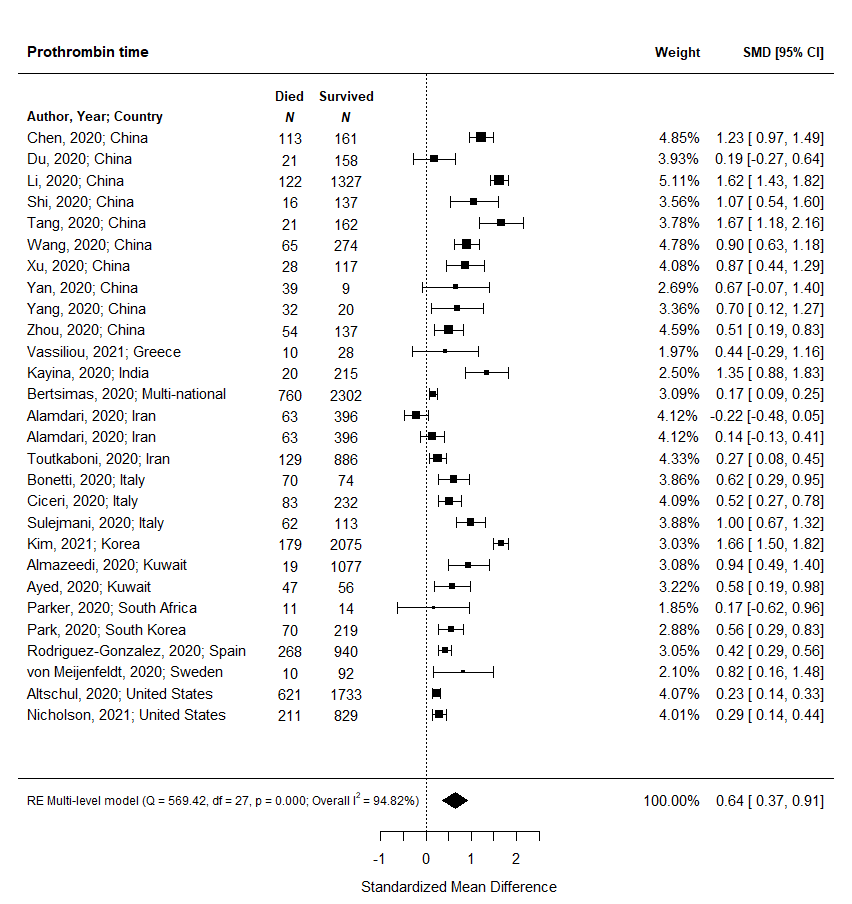


**Figure S35.** Forest plot for prothrombin time (n=27 studies).

**Prothrombin time – Effect by country:**


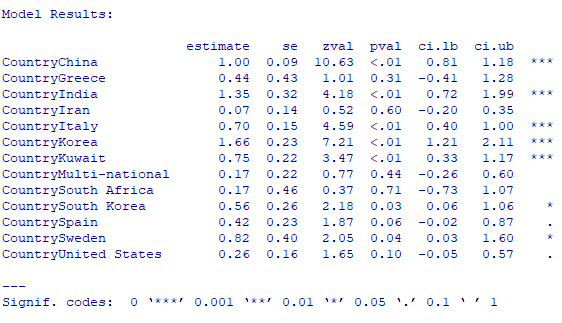


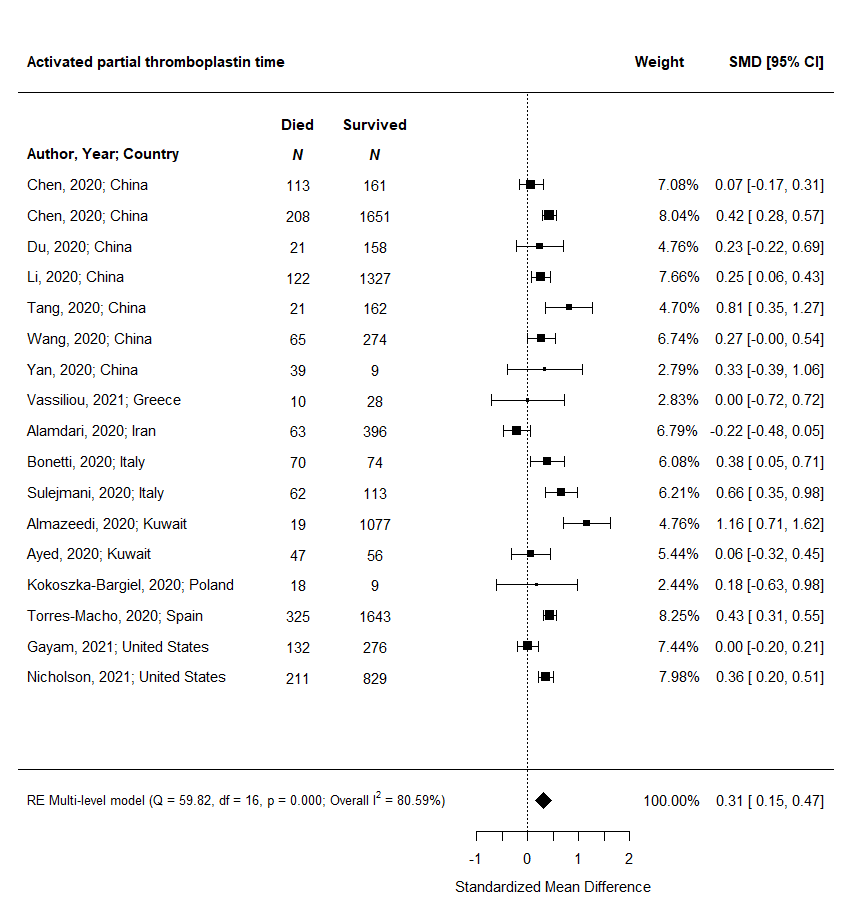


**Figure S36.** Forest plot for Activated partial thromboplastin time (n=17 studies).

**Activated partial thromboplastin time – Effect by country:**


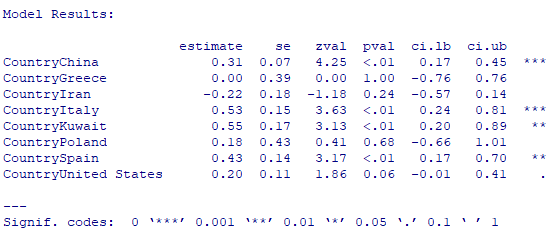


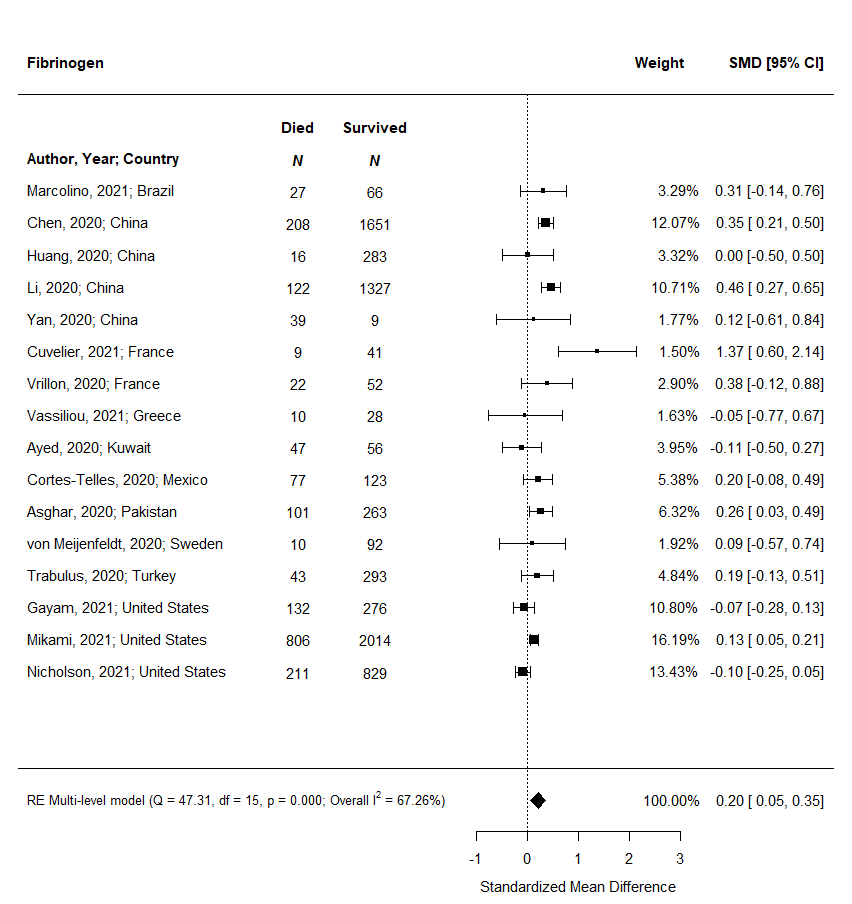


**Figure S37.** Forest plot for Fibrinogen (n=16 studies).

**Fibrinogen – Effect by country:**


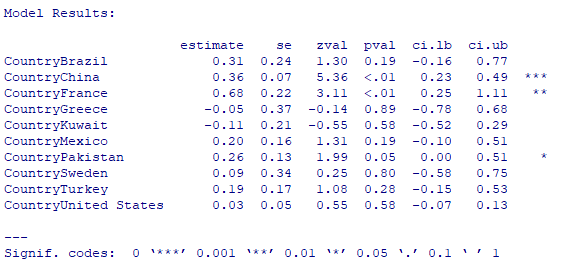


**FOREST PLOTS OF RENAL BIOMARKERS (3 BIOMARKERS)**


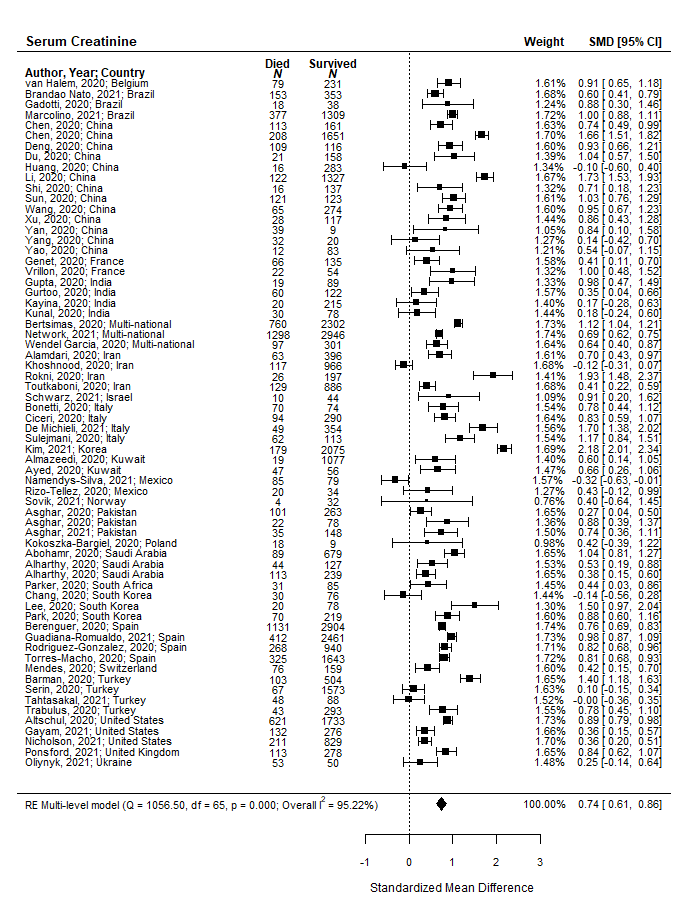


**Figure S38.** Forest plot for creatinine (n=66 studies).

**Serum Creatinine – Effect by country:**


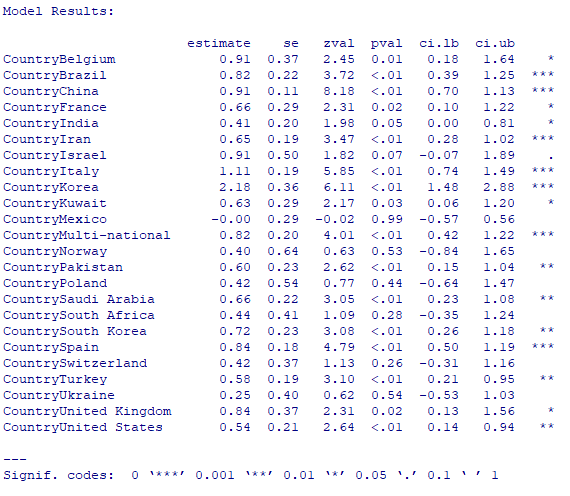


**Estimate** = SMD**; se** = standard error**; ci.lb** = 95% CI lower limit**; ci.ub** = 95% CI upper limit.

**Figure S39.** Forest plot for Fibrinogen (n=16 studies).


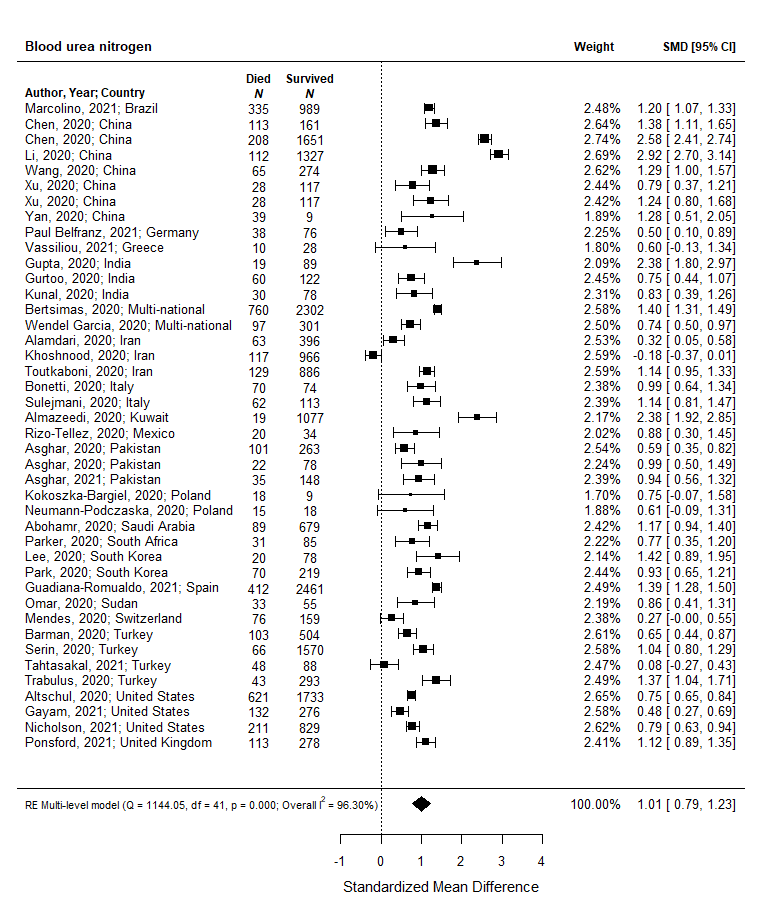


**Figure S40.** Forest plot for blood urea nitrogen (n=41 studies).

**Blood urea nitrogen – Effect by country:**


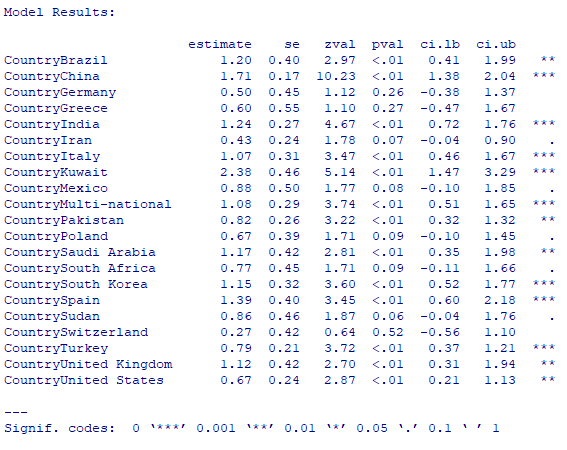


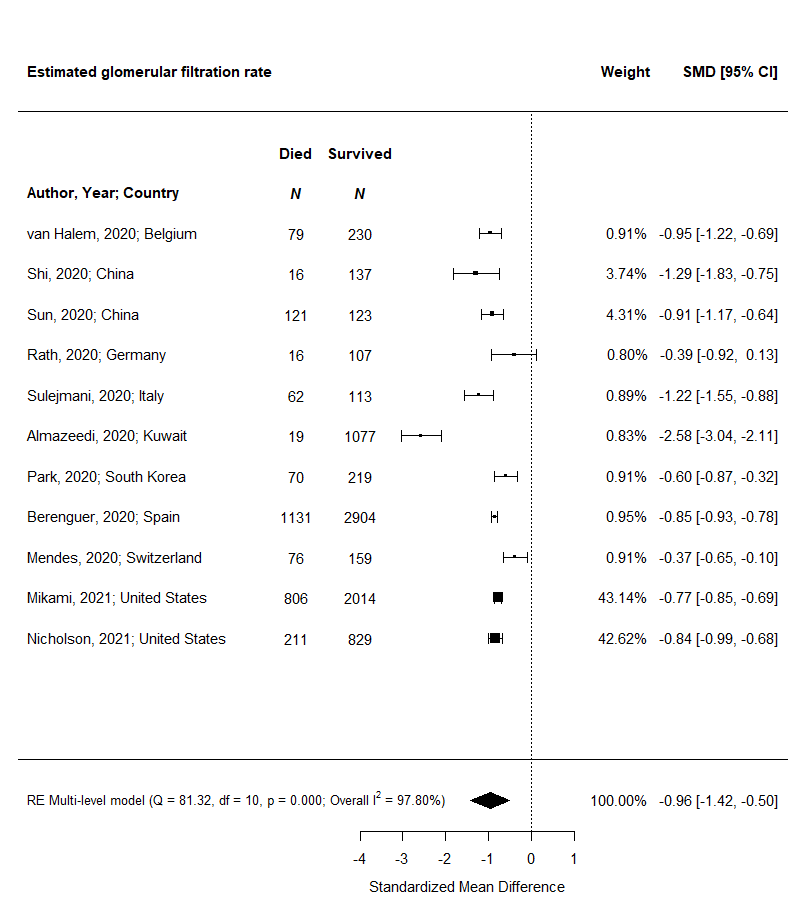


**Figure S41.** Forest plot for Estimated glomerular filtration rate (n=11 studies).

**Estimated glomerular filtration rate – Effect by country:**


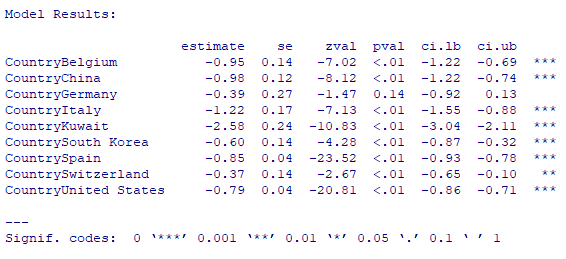


**FOREST PLOTS OF METABOLIC BIOMARKERS (8 BIOMARKERS)**


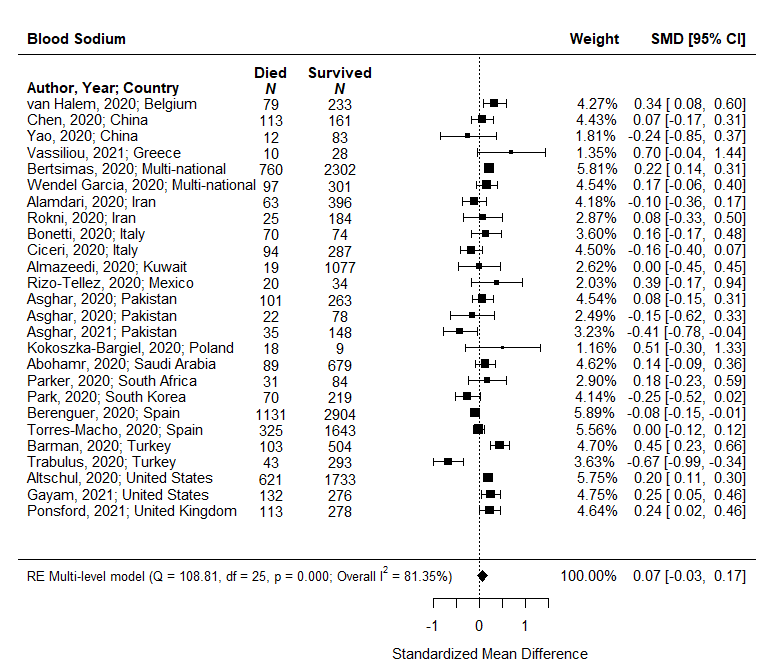


**Figure S42.** Forest plot for sodium (n=26 studies).

**Blood Sodium – Effect by country:**


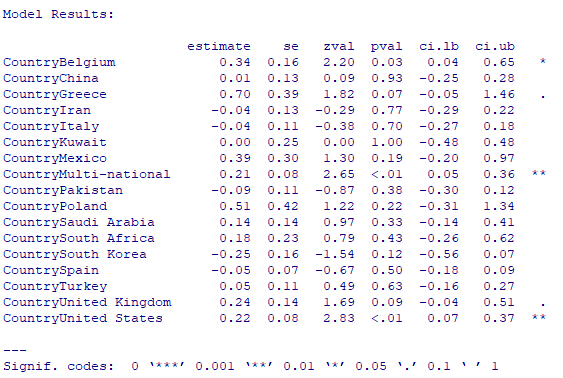


**Estimate** = SMD**; se** = standard error**; ci.lb** = 95% CI lower limit**; ci.ub** = 95% CI upper limit.


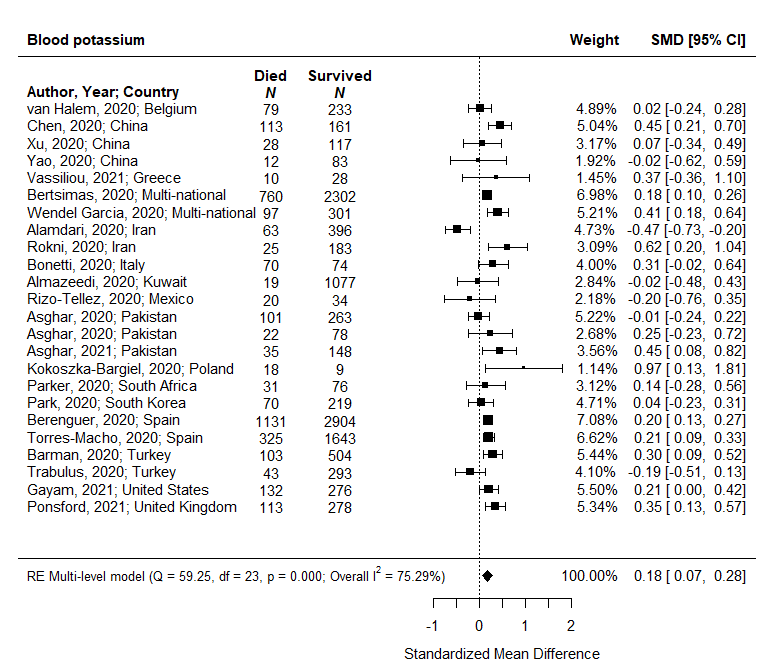


**Figure S43.** Forest plot for potassium (n=24 studies).

**Blood potassium – Effect by country:**


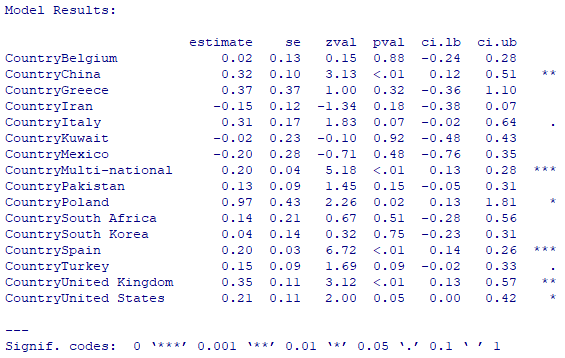


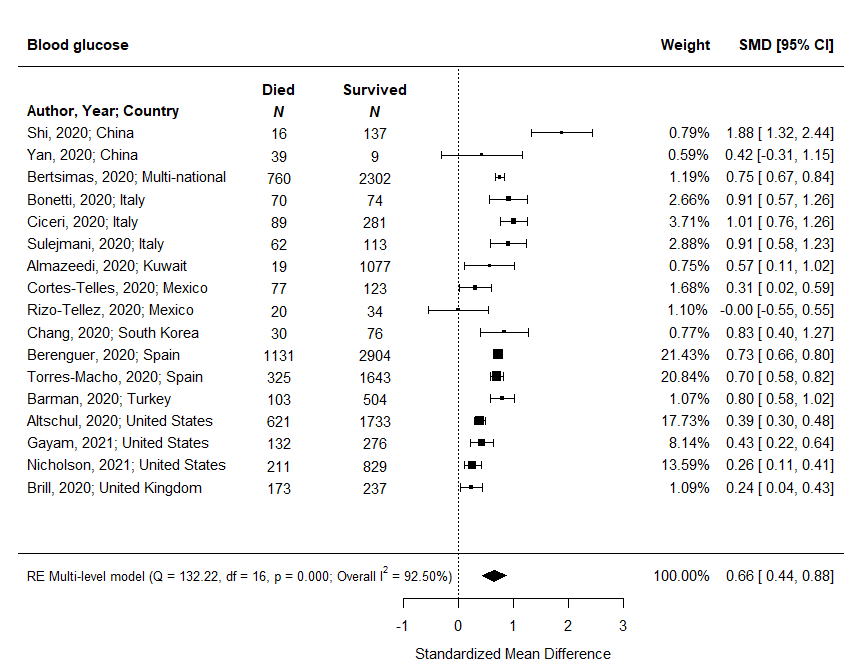


**Figure S44.** Forest plot for blood glucose (n=17 studies).

**Blood glucose – Effect by country:**


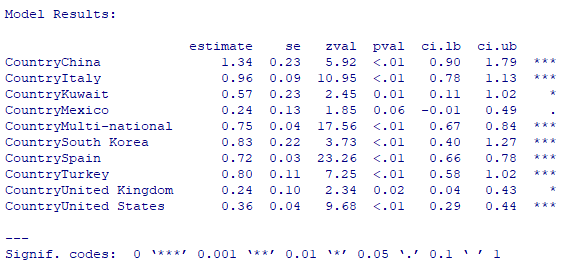


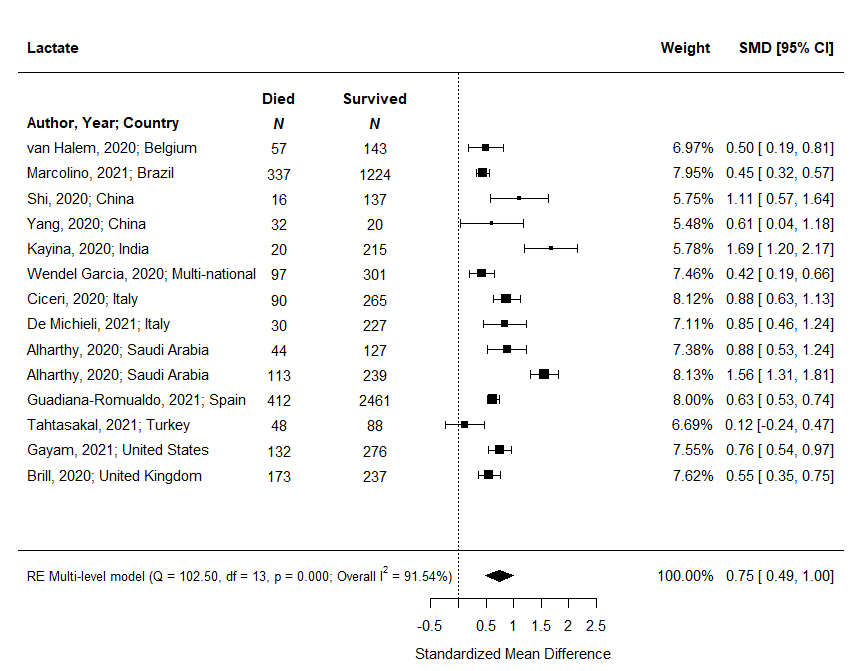


**Figure S45.** Forest plot for lactate (n=13 studies).

**Lactate – Effect by country:**


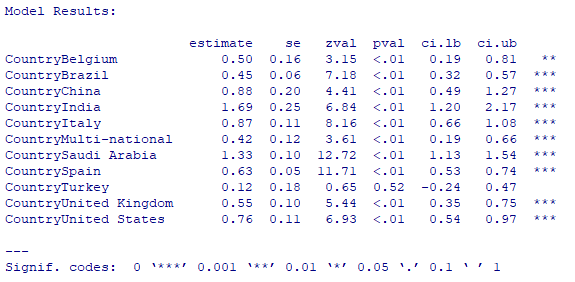


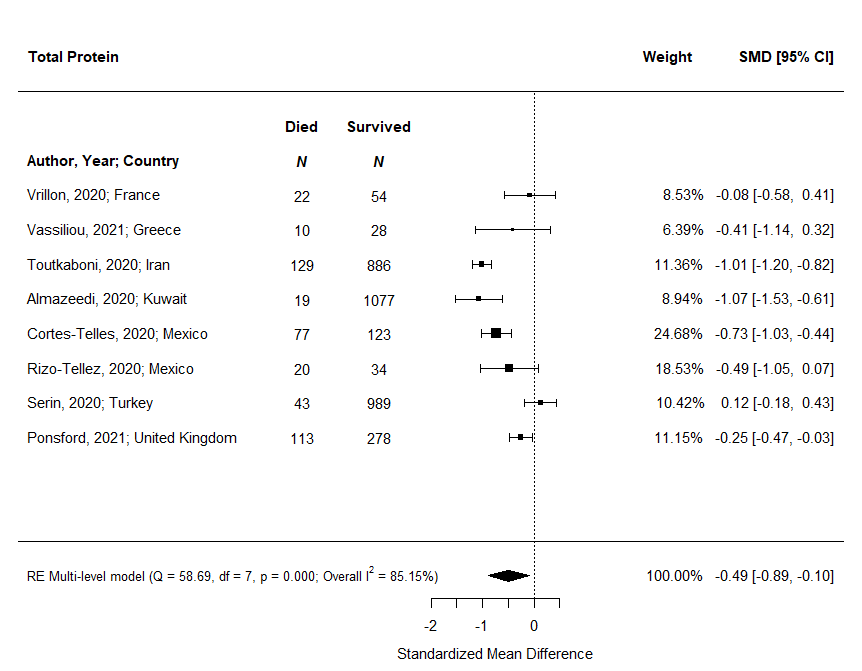


**Figure S46.** Forest plot for total protein (n=8 studies).

**Total Protein – Effect by country:**


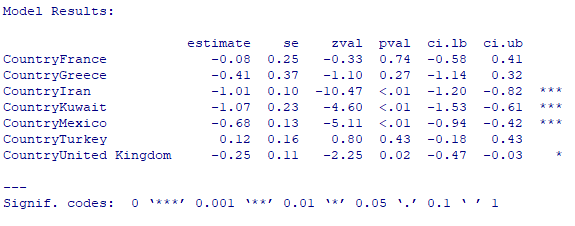


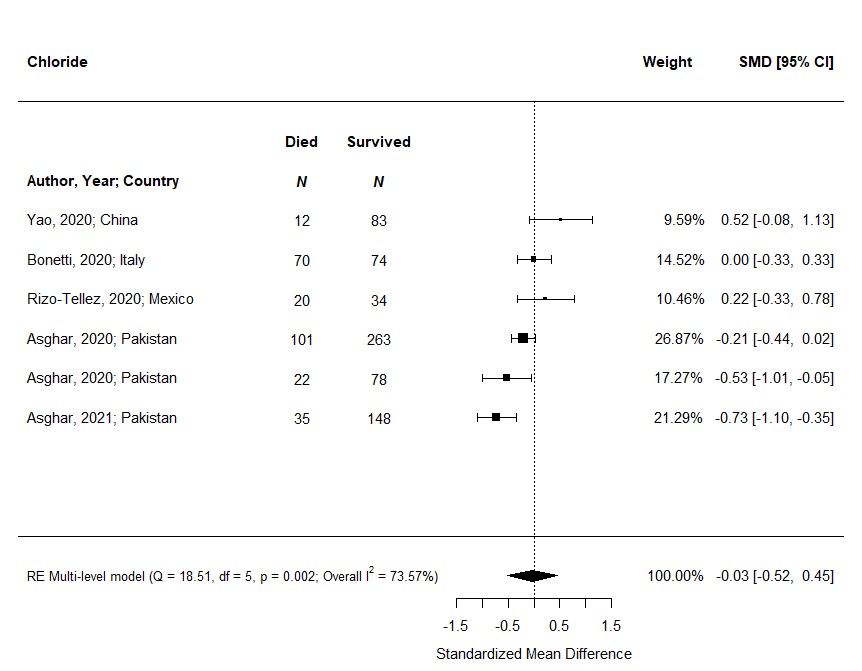


**Figure S47.** Forest plot for chloride (n=6 studies).

**Chloride – Effect by country:**


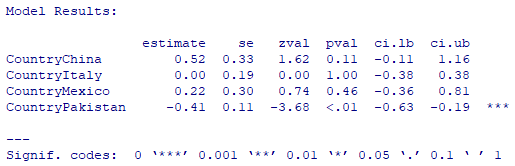


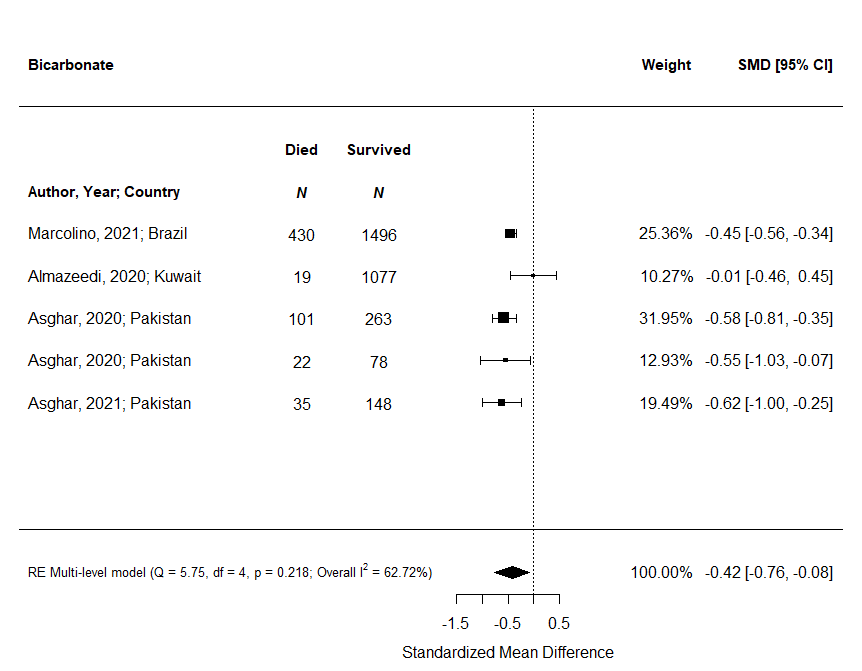
 **Figure S48.** Forest plot for bicarbonate (n=5 studies).

**Bicarbonate – Effect by country:**


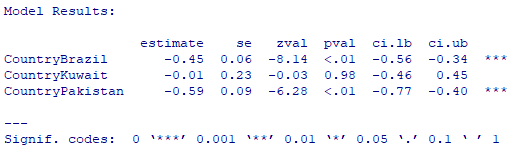


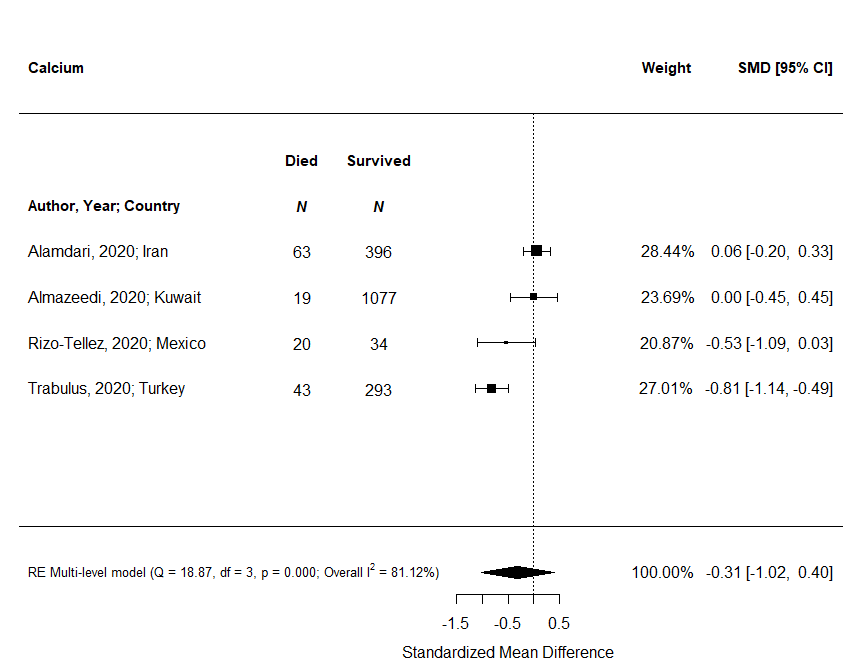


**Figure S49.** Forest plot for calcium (n=4 studies).

**Calcium – Effect by country:**


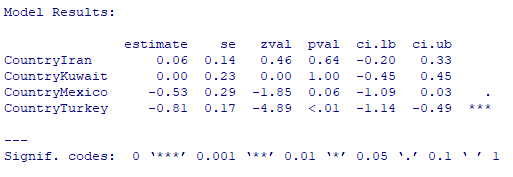


**FOREST PLOTS OF PULMONARY BIOMARKERS (2 BIOMARKERS)**


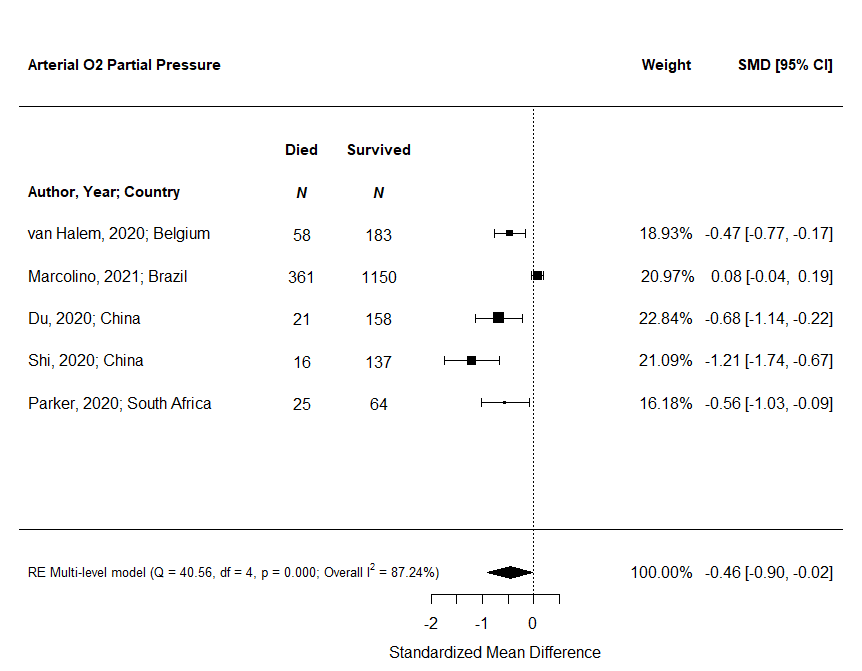


**Figure S50.** Forest plot for Arterial O2 Partial Pressure (n=5 studies).

**Arterial O2 Partial Pressure – Effect by country:**


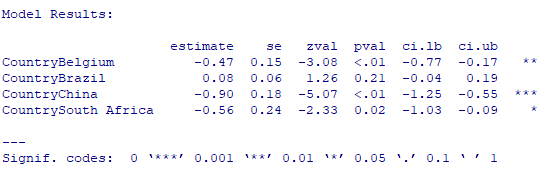


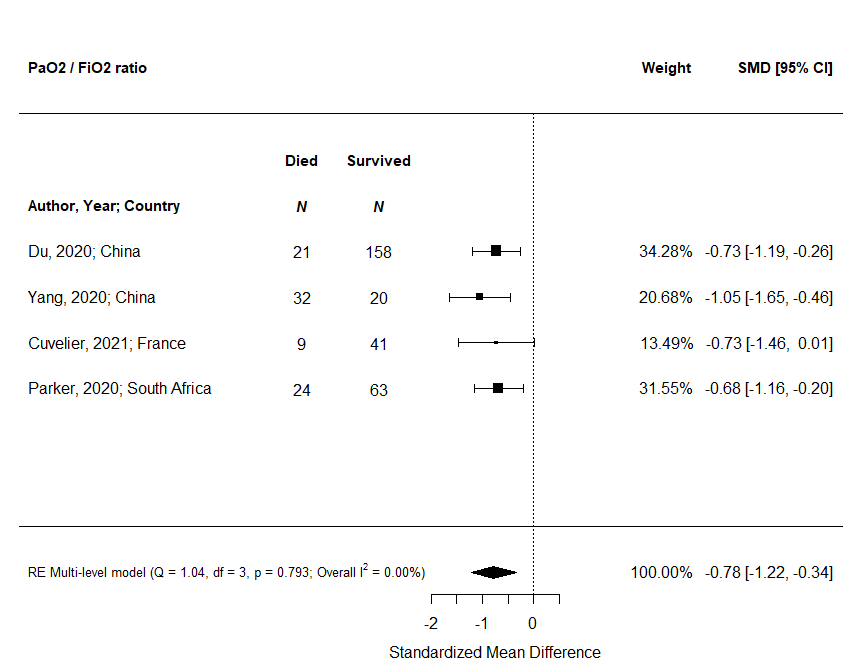


**Figure S51.** Forest plot for PaO2/FiO2 ratio (n=4 studies).

**PaO2/FiO2 ratio – Effect by country:**

**Figure S52.** Bubble plot describing studies that evaluated laboratory biomarkers in relation to mortality (n=81). N=13 studies not displayed in plot due to at least 1 missing value (n=11 missing admission start date, n=2 missing mean age).

**Reference List**

1. Abdeladim S, Oualim S, Elouarradi A, et al. Analysis of cardiac injury biomarkers in COVID-19 patients. *Archives of Clinical Infectious Diseases*. 2020;15(4):1-5. doi:<http://dx.doi.org/10.5812/archcid.105515>

2. Abohamr SI, Abazid RM, Aldossari MA, et al. Clinical characteristics and in-hospital mortality of COVID-19 adult patients in Saudi Arabia. *Saudi Med J*. Nov 2020;41(11):1217-1226. doi:<https://dx.doi.org/10.15537/smj.2020.11.25495>

3. Alamdari NM, Afaghi S, Rahimi FS, et al. Mortality Risk Factors among Hospitalized COVID-19 Patients in a Major Referral Center in Iran. *Tohoku J Exp Med*. 09 2020;252(1):73-84. doi:<https://dx.doi.org/10.1620/tjem.252.73>

4. Alharthy A, Abuhamdah M, Balhamar A, et al. Residual Lung Injury in Patients Recovering From COVID-19 Critical Illness: A Prospective Longitudinal Point-of-Care Lung Ultrasound Study. *Journal of ultrasound in medicine : official journal of the American Institute of Ultrasound in Medicine*. 2020;13doi:<http://dx.doi.org/10.1002/jum.15563>

5. Alharthy A, Aletreby W, Faqihi F, et al. Clinical Characteristics and Predictors of 28-Day Mortality in 352 Critically Ill Patients with COVID-19: A Retrospective Study. *Journal of epidemiology and global health*. 2020;03doi:<http://dx.doi.org/10.2991/jegh.k.200928.001>

6. Almazeedi S, Al-Youha S, Jamal MH, et al. Characteristics, risk factors and outcomes among the first consecutive 1096 patients diagnosed with COVID-19 in Kuwait. *EClinicalMedicine*. July 2020;24 (no pagination)100448. doi:<http://dx.doi.org/10.1016/j.eclinm.2020.100448>

7. Altschul DJ, Unda SR, Benton J, et al. A novel severity score to predict inpatient mortality in COVID-19 patients. Observational Study. *Sci*. 10 07 2020;10(1):16726. doi:<https://dx.doi.org/10.1038/s41598-020-73962-9>

8. Arifputra J, Waleleng BJ, Gosal F, et al. Liver transaminase levels and neutrophil to lymphocyte ratio as prognostic and predictor in coronavirus disease 2019. *Open Access Macedonian Journal of Medical Sciences*. 02 Jan 2020;8(T1):282-285. doi:<http://dx.doi.org/10.3889/oamjms.2020.5395>

9. Asghar MS, Ahmed I, Alvi H, et al. Correlation of refractory hypoxemia with biochemical markers and clinical outcomes of COVID-19 patients in a developing country: A retrospective observational study Running head: Predictors of hypoxemia in COVID-19. *Journal of Community Hospital Internal Medicine Perspectives*. Jan 2021;11(1):9-16. doi:10.1080/20009666.2020.1835214

10. Asghar MS, Khan NA, Kazmi SJH, et al. Hematological parameters predicting severity and mortality in COVID-19 patients of Pakistan: a retrospective comparative analysis. *Journal of Community Hospital Internal Medicine Perspectives*. Nov 2020;10(6):514-520. doi:10.1080/20009666.2020.1816276

11. Asghar MS, Kazmi SJH, Khan NA, et al. Poor Prognostic Biochemical Markers Predicting Fatalities Caused by COVID-19: A Retrospective Observational Study From a Developing Country. *Cureus*. Aug 2020;12(8)doi:10.7759/cureus.9575

12. Asghar MS, Kazmi SJH, Khan NA, et al. Clinical Profiles, Characteristics, and Outcomes of the First 100 Admitted COVID-19 Patients in Pakistan: A Single-Center Retrospective Study in a Tertiary Care Hospital of Karachi. *Cureus*. Jun 2020;12(6)doi:10.7759/cureus.8712

13. Ayed M, Borahmah AA, Yazdani A, Sultan A, Mossad A, Rawdhan H. Assessment of clinical characteristics and mortality-associated factors in COVID-19 Critical cases in Kuwait. *Medical principles and practice : international journal of the Kuwait University, Health Science Centre*. 2020;16doi:<http://dx.doi.org/10.1159/000513047>

14. Balfanz P, Hartmann B, Muller-Wieland D, et al. Early risk markers for severe clinical course and fatal outcome in German patients with COVID-19. Research Support, Non-U.S. Gov't. *PLoS ONE*. 2021;16(1):e0246182. doi:<https://dx.doi.org/10.1371/journal.pone.0246182>

15. Bannaga AS, Tabuso M, Farrugia A, et al. C-reactive protein and albumin association with mortality of hospitalised SARS-CoV-2 patients: A tertiary hospital experience. *Clin Med*. 09 2020;20(5):463-467. doi:<https://dx.doi.org/10.7861/clinmed.2020-0424>

16. Barman HA, Atici A, Sahin I, et al. Prognostic significance of cardiac injury in COVID-19 patients with and without coronary artery disease. *Coronary artery disease*. 2020;19doi:<http://dx.doi.org/10.1097/MCA.0000000000000914>

17. Belaid B, Lamara Mahammad L, Mihi B, et al. T cell counts and IL-6 concentration in blood of North African COVID-19 patients are two independent prognostic factors for severe disease and death. *Journal of Leukocyte Biology*. 2021;doi:<http://dx.doi.org/10.1002/JLB.4COVA1020-703R>

18. Berenguer J, Ryan P, Rodriguez-Bano J, et al. Characteristics and predictors of death among 4035 consecutively hospitalized patients with COVID-19 in Spain. Observational Study. *Clin Microbiol Infect*. Nov 2020;26(11):1525-1536. doi:<https://dx.doi.org/10.1016/j.cmi.2020.07.024>

19. Bertsimas D, Lukin G, Mingardi L, et al. COVID-19 mortality risk assessment: An international multi-center study. Clinical Trial

Multicenter Study

Research Support, Non-U.S. Gov't

Research Support, U.S. Gov't, Non-P.H.S. *PLoS ONE*. 2020;15(12):e0243262. doi:<https://dx.doi.org/10.1371/journal.pone.0243262>

20. Bonetti G, Manelli F, Patroni A, et al. Laboratory predictors of death from coronavirus disease 2019 (COVID-19) in the area of Valcamonica, Italy. *Clin Chem Lab Med*. 06 25 2020;58(7):1100-1105. doi:<https://dx.doi.org/10.1515/cclm-2020-0459>

21. Neto RAB, Marchini JF, Marino LO, et al. Mortality and other outcomes of patients with coronavirus disease pneumonia admitted to the emergency department: A prospective observational Brazilian study. *PLoS ONE*. Jan 2021;16(1)doi:10.1371/journal.pone.0244532

22. Brill SE, Jarvis HC, Ozcan E, et al. COVID-19: A retrospective cohort study with focus on the over-80s and hospital-onset disease. *BMC Medicine*. 2020;18(1)194. doi:<http://dx.doi.org/10.1186/s12916-020-01665-z>

23. Chang MC, Hwang JM, Jeon JH, Kwak SG, Park D, Moon JS. Fasting Plasma Glucose Level Independently Predicts the Mortality of Patients with Coronavirus Disease 2019 Infection: A Multicenter, Retrospective Cohort Study. Evaluation Study Multicenter Study

Research Support, Non-U.S. Gov't. *Endocrinol Metab (Seoul)*. 09 2020;35(3):595-601. doi:<https://dx.doi.org/10.3803/EnM.2020.719>

24. Chen T, Wu D, Chen H, et al. Clinical characteristics of 113 deceased patients with coronavirus disease 2019: retrospective study. *Bmj*. Mar 26 2020;368:m1091. doi:<https://dx.doi.org/10.1136/bmj.m1091>

25. Chen L, Yu J, He W, et al. Risk factors for death in 1859 subjects with COVID-19. Research Support, Non-U.S. Gov't. *Leukemia*. 08 2020;34(8):2173-2183. doi:<https://dx.doi.org/10.1038/s41375-020-0911-0>

26. Ciceri F, Castagna A, Rovere-Querini P, et al. Early predictors of clinical outcomes of COVID-19 outbreak in Milan, Italy. *Clin Immunol*. August 2020;217 (no pagination)108509. doi:<http://dx.doi.org/10.1016/j.clim.2020.108509>

27. Cortes-Telles A, Lopez-Romero S, Mancilla-Ceballos R, Ortiz-Farias DL, Nunez-Caamal N, Figueroa-Hurtado E. Risk factors for mortality in hospitalized patients with COVID-19: An overview in a Mexican population. *Tuberculosis and Respiratory Diseases*. 20 Oct 2020;83:S46-S54. doi:<http://dx.doi.org/10.4046/TRD.2020.0095>

28. Network C-IGobotR, the C-ICUI. Clinical characteristics and day-90 outcomes of 4244 critically ill adults with COVID-19: a prospective cohort study. Multicenter Study. *Intensive Care Med*. 01 2021;47(1):60-73. doi:<https://dx.doi.org/10.1007/s00134-020-06294-x>

29. Cuvelier P, Roux H, Couedel-Courteille A, et al. Protective reactive thymus hyperplasia in COVID-19 acute respiratory distress syndrome. Observational Study

Research Support, Non-U.S. Gov't. *Crit Care*. 01 04 2021;25(1):4. doi:<https://dx.doi.org/10.1186/s13054-020-03440-1>

30. Deng Y, Liu W, Liu K, et al. Clinical characteristics of fatal and recovered cases of coronavirus disease 2019 (COVID-19) in Wuhan, China: a retrospective study. *Chin Med J*. Mar 20 2020;20:20. doi:<https://dx.doi.org/10.1097/CM9.0000000000000824>

31. De Michieli L, Babuin L, Vigolo S, et al. Using high sensitivity cardiac troponin values in patients with SARS-CoV-2 infection (COVID-19): The Padova experience. *Clinical Biochemistry*. 2021;doi:<http://dx.doi.org/10.1016/j.clinbiochem.2021.01.006>

32. De Smet R, Mellaerts B, Vandewinckele H, et al. Frailty and Mortality in Hospitalized Older Adults With COVID-19: Retrospective Observational Study. Observational Study. *J Am Med Dir Assoc*. Jul 2020;21(7):928-932.e1. doi:<https://dx.doi.org/10.1016/j.jamda.2020.06.008>

33. Du RH, Liang LR, Yang CQ, et al. Predictors of mortality for patients with COVID-19 pneumonia caused by SARSCoV- 2: A prospective cohort study. *Eur Respir J*. 2020;56(3)doi:<http://dx.doi.org/10.1183/13993003.50524-2020>

34. Gadotti AC, de Castro Deus M, Telles JP, et al. IFN-gamma is an independent risk factor associated with mortality in patients with moderate and severe COVID-19 infection. *Virus Res*. 11 2020;289:198171. doi:<https://dx.doi.org/10.1016/j.virusres.2020.198171>

35. Garcia de Guadiana-Romualdo L, Morell-Garcia D, Morales-Indiano C, et al. Characteristics and laboratory findings on admission to the emergency department among 2873 hospitalized patients with COVID-19: the impact of adjusted laboratory tests in multicenter studies. A multicenter study in Spain (BIOCOVID-Spain study). *Scandinavian Journal of Clinical and Laboratory Investigation*. 2021;doi:<http://dx.doi.org/10.1080/00365513.2021.1881997>

36. Gayam V, Chobufo MD, Merghani MA, Lamichhane S, Garlapati PR, Adler MK. Clinical characteristics and predictors of mortality in African-Americans with COVID-19 from an inner-city community teaching hospital in New York. *J Med Virol*. 02 2021;93(2):812-819. doi:<https://dx.doi.org/10.1002/jmv.26306>

37. Genet B, Vidal JS, Cohen A, et al. COVID-19 In-Hospital Mortality and Use of Renin-Angiotensin System Blockers in Geriatrics Patients. Observational Study. *J Am Med Dir Assoc*. 11 2020;21(11):1539-1545. doi:<https://dx.doi.org/10.1016/j.jamda.2020.09.004>

38. Guo T, Fan Y, Chen M, et al. Cardiovascular Implications of Fatal Outcomes of Patients With Coronavirus Disease 2019 (COVID-19). *JAMA Cardiol*. Mar 27 2020;27:27. doi:<https://dx.doi.org/10.1001/jamacardio.2020.1017>

39. Gupta N, Ish P, Kumar R, et al. Evaluation of the clinical profile, laboratory parameters and outcome of two hundred COVID-19 patients from a tertiary centre in India. Observational Study. *Monaldi Arch Chest Dis*. Nov 09 2020;90(4):09. doi:<https://dx.doi.org/10.4081/monaldi.2020.1507>

40. Gurtoo A, Agrawal A, Prakash A, et al. The Syndromic Spectrum of COVID-19 and Correlates of Admission Parameters with Severity-outcome Gradients: A Retrospective Study. *J Assoc Physicians India*. Dec 2020;68(12):43-48.

41. Huang J, Cheng A, Kumar R, et al. Hypoalbuminemia predicts the outcome of COVID-19 independent of age and co-morbidity. *J Med Virol*. 01 Oct 2020;92(10):2152-2158. doi:<http://dx.doi.org/10.1002/jmv.26003>

42. Kayina CA, Haritha D, Soni L, et al. Epidemiological & clinical characteristics & early outcome of COVID-19 patients in a tertiary care teaching hospital in India: A preliminary analysis. *Indian J Med Res*. Jul & Aug 2020;152(1 & 2):100-104. doi:<https://dx.doi.org/10.4103/ijmr.IJMR_2890_20>

43. Khoshnood RJ, Ommi D, Zali A, et al. Epidemiological Characteristics, Clinical Features, and Outcome of COVID-19 Patients in Northern Tehran, Iran; a Cross-Sectional Study. *Advanced Journal of Emergency Medicine*. Feb 2021;5(1)doi:10.22114/ajem.v0i0.547

44. Kim SW, Kim SM, Kim YK, et al. Clinical Characteristics and Outcomes of COVID-19 Cohort Patients in Daegu Metropolitan City Outbreak in 2020. Multicenter Study. *J Korean Med Sci*. Jan 04 2021;36(1):e12. doi:<https://dx.doi.org/10.3346/jkms.2021.36.e12>

45. Kokoszka-Bargiel I, Cyprys P, Rutkowska K, Madowicz J, Knapik P. Intensive care unit admissions during the first 3 months of the COVID-19 pandemic in Poland: A single-center, cross-sectional study. *Med Sci Monit*. 2020;26 (no pagination)e926974. doi:<http://dx.doi.org/10.12659/MSM.926974>

46. Kunal S, Sharma SM, Sharma SK, et al. Cardiovascular complications and its impact on outcomes in COVID-19. Observational Study. *Indian Heart J*. Nov-Dec 2020;72(6):593-598. doi:<https://dx.doi.org/10.1016/j.ihj.2020.10.005>

47. Lee JY, Kim HA, Huh K, et al. Risk Factors for Mortality and Respiratory Support in Elderly Patients Hospitalized with COVID-19 in Korea. *J Korean Med Sci*. Jun 15 2020;35(23):e223. doi:<https://dx.doi.org/10.3346/jkms.2020.35.e223>

48. Li H, Xiang X, Ren H, et al. Serum Amyloid A is a biomarker of severe Coronavirus Disease and poor prognosis. *J Infect*. 2020;80(6):646-655.

49. Li Q, Cao Y, Chen L, et al. Hematological features of persons with COVID-19. Research Support, Non-U.S. Gov't. *Leukemia*. 08 2020;34(8):2163-2172. doi:<https://dx.doi.org/10.1038/s41375-020-0910-1>

50. Mandel M, Harari G, Gurevich M, Achiron A. Cytokine prediction of mortality in COVID19 patients. *Cytokine*. 10 2020;134:155190. doi:<https://dx.doi.org/10.1016/j.cyto.2020.155190>

51. Marcolino MS, Ziegelmann PK, Souza-Silva MVR, et al. Clinical characteristics and outcomes of patients hospitalized with COVID-19 in Brazil: results from the Brazilian COVID-19 Registry. *International journal of infectious diseases : IJID : official publication of the International Society for Infectious Diseases*. 2021;11doi:<http://dx.doi.org/10.1016/j.ijid.2021.01.019>

52. Mendes A, Serratrice C, Herrmann FR, et al. Predictors of In-Hospital Mortality in Older Patients With COVID-19: The COVIDAge Study. *J Am Med Dir Assoc*. 11 2020;21(11):1546-1554.e3. doi:<https://dx.doi.org/10.1016/j.jamda.2020.09.014>

53. Mikami T, Miyashita H, Yamada T, et al. Risk Factors for Mortality in Patients with COVID-19 in New York City. *J Gen Intern Med*. 01 2021;36(1):17-26. doi:<https://dx.doi.org/10.1007/s11606-020-05983-z>

54. Namendys-Silva SA, Alvarado-Avila PE, Dominguez-Cherit G, et al. Outcomes of patients with COVID-19 in the intensive care unit in Mexico: A multicenter observational study. Multicenter Study

Observational Study. *Heart Lung*. Jan - Feb 2021;50(1):28-32. doi:<https://dx.doi.org/10.1016/j.hrtlng.2020.10.013>

55. Neumann-Podczaska A, Chojnicki M, Karbowski LM, et al. Clinical Characteristics and Survival Analysis in a Small Sample of Older COVID-19 Patients with Defined 60-Day Outcome. *Int J Environ Res Public Health*. Nov 12 2020;17(22):12. doi:<https://dx.doi.org/10.3390/ijerph17228362>

56. Nicholson CJ, Wooster L, Sigurslid HH, et al. Estimating risk of mechanical ventilation and in-hospital mortality among adult COVID-19 patients admitted to Mass General Brigham: The VICE and DICE scores. *EClinicalMedicine*. March 2021;33 (no pagination)100765. doi:<http://dx.doi.org/10.1016/j.eclinm.2021.100765>

57. Omar SM, Musa IR, Salah SE, Elnur MM, Al-Wutayd O, Adam I. High mortality rate in adult covid-19 inpatients in Eastern Sudan: A retrospective study. *Journal of Multidisciplinary Healthcare*. 2020;13:1887-1893. doi:<http://dx.doi.org/10.2147/JMDH.S283900>

58. Oliynyk OV, Rorat M, Barg W. Oxygen metabolism markers as predictors of mortality in severe COVID-19. *Int J Infect Dis*. February 2021;103:452-456. doi:<http://dx.doi.org/10.1016/j.ijid.2020.12.012>

59. Park JG, Kang MK, Lee YR, et al. Fibrosis-4 index as a predictor for mortality in hospitalised patients with COVID-19: a retrospective multicentre cohort study. Multicenter Study

Research Support, Non-U.S. Gov't. *BMJ Open*. 11 12 2020;10(11):e041989. doi:<https://dx.doi.org/10.1136/bmjopen-2020-041989>

60. Parker A, Koegelenberg CFN, Moolla MS, et al. High HIV prevalence in an early cohort of hospital admissions with COVID-19 in Cape Town, South Africa. Observational Study. *S Afr Med J*. 08 21 2020;110(10):982-987. doi:<https://dx.doi.org/10.7196/SAMJ.2020.v110i10.15067>

61. Ponsford MJ, Burton RJ, Smith L, et al. Examining the utility of extended laboratory panel testing in the emergency department for risk stratification of patients with COVID-19: A single-centre retrospective service evaluation. *J Clin Pathol*. 2021;(no pagination)2020207157. doi:<http://dx.doi.org/10.1136/jclinpath-2020-207157>

62. Rath D, Petersen-Uribe A, Avdiu A, et al. Impaired cardiac function is associated with mortality in patients with acute COVID-19 infection. *Clinical Research in Cardiology*. December 2020;109(12):1491-1499. doi:<http://dx.doi.org/10.1007/s00392-020-01683-0>

63. Rizo-Tellez SA, Mendez-Garcia LA, Flores-Rebollo C, et al. The Neutrophil-to-Monocyte Ratio and Lymphocyte-to-Neutrophil Ratio at Admission Predict In-Hospital Mortality in Mexican Patients with Severe SARS-CoV-2 Infection (Covid-19). *Microorganisms*. Oct 2020;8(10)doi:10.3390/microorganisms8101560

64. Rodriguez-Gonzalez CG, Chamorro-de-Vega E, Valerio M, et al. COVID-19 in hospitalised patients in Spain: a cohort study in Madrid. *Int J Antimicrob Agents*. Feb 2021;57(2):106249. doi:<https://dx.doi.org/10.1016/j.ijantimicag.2020.106249>

65. Rokni M, Ahmadikia K, Asghari S, Mashaei S, Hassanali F. Comparison of clinical, para-clinical and laboratory findings in survived and deceased patients with COVID-19: diagnostic role of inflammatory indications in determining the severity of illness. Comparative Study. *BMC Infect Dis*. Nov 23 2020;20(1):869. doi:<https://dx.doi.org/10.1186/s12879-020-05540-3>

66. Sun H, Ning R, Tao Y, et al. Risk Factors for Mortality in 244 Older Adults With COVID-19 in Wuhan, China: A Retrospective Study. *J Am Geriatr Soc*. 01 Jun 2020;68(6):E19-E23. doi:<http://dx.doi.org/10.1111/jgs.16533>

67. Schwarz Y, Percik R, Oberman B, Yaffe D, Zimlichman E, Tirosh A. Sick Euthyroid Syndrome on Presentation of Patients With COVID-19: A Potential Marker for Disease Severity. *Endocr Pract*. Feb 2021;27(2):101-109. doi:<https://dx.doi.org/10.1016/j.eprac.2021.01.001>

68. Sensusiati AD, Amin M, Nasronudin N, et al. Age, neutrophil lymphocyte ratio, and radiographic assessment of the quantity of lung edema (RALE) score to predict in-hospital mortality in COVID-19 patients: a retrospective study. *F1000Res*. 2020;9:1286. doi:<https://dx.doi.org/10.12688/f1000research.26723.2>

69. Serin I, Sari ND, Dogu MH, et al. A new parameter in COVID-19 pandemic: initial lactate dehydrogenase (LDH)/Lymphocyte ratio for diagnosis and mortality. *J Infect Public Health*. Nov 2020;13(11):1664-1670. doi:<https://dx.doi.org/10.1016/j.jiph.2020.09.009>

70. Shi Q, Jiang F, Zhang X, et al. Clinical Characteristics and Risk Factors for Mortality of COVID-19 Patients with Diabetes in Wuhan, China: A Two-Center, Retrospective Study. *Diabetes Care*. 01 Jul 2020;43(7):1382-1391. doi:<http://dx.doi.org/10.2337/dc20-0598>

71. Sovik S, Badstolokken PM, Sorensen V, et al. A single-centre, prospective cohort study of COVID-19 patients admitted to ICU for mechanical ventilatory support. Observational Study. *Acta Anaesthesiol Scand*. 03 2021;65(3):351-359. doi:<https://dx.doi.org/10.1111/aas.13726>

72. Sulejmani A, Galimberti E, Giacobone C, et al. Baseline characteristics of COVID-19 Italian patients admitted to Desio Hospital, Lombardy: a retrospective study. *Scand J Clin Lab Invest*. 02 2021;81(1):18-23. doi:<https://dx.doi.org/10.1080/00365513.2020.1846211>

73. Sweeney TE, Liesenfeld O, Wacker J, et al. Validation of Inflammopathic, Adaptive, and Coagulopathic Sepsis Endotypes in Coronavirus Disease 2019. *Crit Care Med*. 02 01 2021;49(2):e170-e178. doi:<https://dx.doi.org/10.1097/CCM.0000000000004786>

74. Tahtasakal CA, Oncul A, Sevgi DY, et al. Could we predict the prognosis of the COVID-19 disease? *J Med Virol*. April 2021;93(4):2420-2430. doi:<http://dx.doi.org/10.1002/jmv.26751>

75. Tang N, Bai H, Chen X, Gong J, Li D, Sun Z. Anticoagulant treatment is associated with decreased mortality in severe coronavirus disease 2019 patients with coagulopathy. *J Thromb Haemost*. Mar 27 2020;27:27. doi:<https://dx.doi.org/10.1111/jth.14817>

76. Tang N, Li D, Wang X, Sun Z. Abnormal coagulation parameters are associated with poor prognosis in patients with novel coronavirus pneumonia. *J Thromb Haemost*. Feb 19 2020;19:19. doi:<https://dx.doi.org/10.1111/jth.14768>

77. Thompson JV, Meghani NJ, Powell BM, et al. Patient characteristics and predictors of mortality in 470 adults admitted to a district general hospital in England with Covid-19. *Epidemiol Infect*. 11 24 2020;148:e285. doi:<https://dx.doi.org/10.1017/S0950268820002873>

78. Torres-Macho J, Ryan P, Valencia J, et al. The pandemyc score. An easily applicable and interpretable model for predicting mortality associated with COVID-19. *J*. October 2020;9(10):1-10. doi:<http://dx.doi.org/10.3390/jcm9103066>

79. Pourabdollah Toutkaboni M, Askari E, Khalili N, et al. Demographics, laboratory parameters and outcomes of 1061 patients with coronavirus disease 2019: a report from Tehran, Iran. *New Microbes and New Infections*. November 2020;38 (no pagination)100777. doi:<http://dx.doi.org/10.1016/j.nmni.2020.100777>

80. Trabulus S, Karaca C, Balkan II, et al. Kidney function on admission predicts in-hospital mortality in COVID-19. *PLoS ONE*. 2020;15(9 september)e0238680. doi:<http://dx.doi.org/10.1371/journal.pone.0238680>

81. van Halem K, Bruyndonckx R, van der Hilst J, et al. Risk factors for mortality in hospitalized patients with COVID-19 at the start of the pandemic in Belgium: a retrospective cohort study. *BMC Infect Dis*. Nov 27 2020;20(1):897. doi:<https://dx.doi.org/10.1186/s12879-020-05605-3>

82. Vassiliou AG, Keskinidou C, Jahaj E, et al. ICU admission levels of endothelial biomarkers as predictors of mortality in critically ill COVID-19 patients. *Cells*. January 2021;10(1):1-13. doi:<http://dx.doi.org/10.3390/cells10010186>

83. von Meijenfeldt FA, Havervall S, Adelmeijer J, et al. Prothrombotic changes in patients with COVID-19 are associated with disease severity and mortality. *Research and Practice in Thrombosis and Haemostasis*. January 2021;5(1):132-141. doi:<http://dx.doi.org/10.1002/rth2.12462>

84. Vrillon A, Hourregue C, Azuar J, et al. COVID-19 in Older Adults: A Series of 76 Patients Aged 85 Years and Older with COVID-19. *J Am Geriatr Soc*. 12 2020;68(12):2735-2743. doi:<https://dx.doi.org/10.1111/jgs.16894>

85. Wang L, He WB, Yu XM, et al. Coronavirus disease 2019 in elderly patients: Characteristics and prognostic factors based on 4-week follow-up. *J Infect*. Jun 2020;80(6):639-645. doi:10.1016/j.jinf.2020.03.019

86. Wendel Garcia PD, Fumeaux T, Guerci P, et al. Prognostic factors associated with mortality risk and disease progression in 639 critically ill patients with COVID-19 in Europe: Initial report of the international RISC-19-ICU prospective observational cohort. *EClinicalMedicine*. August 2020;25 (no pagination)100449. doi:<http://dx.doi.org/10.1016/j.eclinm.2020.100449>

87. Xu B, Fan CY, Wang AL, et al. Suppressed T cell-mediated immunity in patients with COVID-19: A clinical retrospective study in Wuhan, China. *J Infect*. July 2020;81(1):e51-e60. doi:<http://dx.doi.org/10.1016/j.jinf.2020.04.012>

88. Yan YL, Yang Y, Wang F, et al. Clinical characteristics and outcomes of patients with severe covid-19 with diabetes. *Bmj Open Diabetes Research & Care*. Jan 2020;8(1)doi:10.1136/bmjdrc-2020-001343

89. Yang X, Yu Y, Xu J, et al. Clinical course and outcomes of critically ill patients with SARS-CoV-2 pneumonia in Wuhan, China: a single-centered, retrospective, observational study. *Lancet Respir Med*. Feb 24 2020;24:24. doi:<https://dx.doi.org/10.1016/S2213-2600(20)30079-5>

90. Yang X, Yang Q, Wang Y, et al. Thrombocytopenia and its association with mortality in patients with COVID-19. *J Thromb Haemost*. Jun 2020;18(6):1469-1472. doi:10.1111/jth.14848

91. Yao Q, Wang P, Wang X, et al. Retrospective study of risk factors for severe SARS-Cov-2 infections in hospitalized adult patients. *Pol Arch Intern Med*. 2020;

92. Zhao X, Wang K, Zuo P, et al. Early decrease in blood platelet count is associated with poor prognosis in COVID-19 patients-indications for predictive, preventive, and personalized medical approach. *EPMA Journal*. 01 Jun 2020;11(2):139-145. doi:<http://dx.doi.org/10.1007/s13167-020-00208-z>

93. Zhang JP, Liu P, Wang MR, et al. The clinical data from 19 critically ill patients with coronavirus disease 2019: a single-centered, retrospective, observational study. *Journal of Public Health-Heidelberg*. doi:10.1007/s10389-020-01291-2

94. Zhou F, Yu T, Du R, et al. Clinical course and risk factors for mortality of adult inpatients with COVID-19 in Wuhan, China: a retrospective cohort study. *Lancet*. Mar 28 2020;395(10229):1054-1062. doi:<https://dx.doi.org/10.1016/S0140-6736(20)30566-3>
